# Sero-Surveillance to Monitor the Trend of SARS-CoV-2 Infection Transmission in India: Study Protocol for a Multi Site, Community Based Longitudinal Cohort Study

**DOI:** 10.3389/fpubh.2022.810353

**Published:** 2022-03-24

**Authors:** Divya Nair, Reshma Raju, Sudipto Roy, Shailendra Dandge, Girish Kumar Chethrapilly Purushothaman, Yuvaraj Jayaraman, Boopathi Kangusamy, Rahul Shrivastava, Narendra Kumar Arora, Winsley Rose, Sanjay Juvekar, Guru Rajesh Jammy, Kavita Singh, Sanjay Mehendale, Prabu Rajkumar, Shikha Taneja Malik

**Affiliations:** ^1^Department of Research, The International Clinical Epidemiology Network (INCLEN) Trust International, New Delhi, India; ^2^Department of Pediatrics, Christian Medical College, Vellore, India; ^3^Division of Epidemiology and Communicable Diseases, Indian Council of Medical Research, New Delhi, India; ^4^Society for Health Allied Research and Education India, Hyderabad, India; ^5^Indian Council of Medical Research (ICMR) - National Institute of Epidemiology, Chennai, India; ^6^Biotechnology Industry Research Assistance Council, New Delhi, India; ^7^Vadu Rural Health Program, King Edward Memorial (KEM) Hospital Research Center, Pune, India; ^8^Parmanand Deepchand (PD) Hinduja Hospital and Medical Research Center, Mumbai, India

**Keywords:** SARS-CoV-2, sero-surveillance, epidemiological studies, cohort, COVID-19, acute febrile illness surveillance

## Abstract

**Introduction:**

Large-scale sero-prevalence studies with representation from all age groups are required to estimate the true burden of severe acute respiratory syndrome coronavirus 2 (SARS-CoV-2) infections in the community. Serial serological surveys in fixed cohorts enable study of dynamics of viral transmission and correlates of immune response over time in the context of gradual introduction of COVID-19 vaccines and repeated upsurge of cases during the pandemic.

**Methods:**

This longitudinal study will involve follow-up of a cohort of 25,000 individuals (5,000 per site) aged 2 years and above recruited from five existing demographic surveillance sites in India. The cohort will be tested for the presence of IgG antibodies against S1/S2 spike protein subunits of SARS-CoV-2 in four rounds; once at baseline and subsequently, at intervals of 4 months for a year between January 2021 and January 2022. Neutralization assays will be carried out in a subset of seropositive samples in each round to quantify the antibody response and to estimate the durability of antibody response. Serial serological surveys will be complemented by fortnightly phone based syndromic surveillance to assess the burden of symptomatic acute febrile illness/ influenza like illness in the same cohort. A bio-repository will also be established to store the serum samples collected in all rounds of serological surveys.

**Discussion:**

The population based sero-epidemiological studies will help to determine the burden of COVID-19 at the community level in urban and rural Indian populations and guide in monitoring the trends in the transmission of SARS-CoV-2 infection. Risk factors for infection will be identified to inform future control strategies. The serial serological surveys in the same set of participants will help determine the viral transmission dynamics and durability of neutralizing immune response in participants with or without symptomatic COVID infection.

## Introduction

Coronavirus disease (COVID-19) caused by the novel severe acute respiratory syndrome coronavirus 2 (SARS-CoV-2) that emerged in Wuhan, China, at the end of 2019, has become a pandemic of unprecedented proportions across the world. Globally, 200 million confirmed cases of COVID-19, including about 4 million deaths have been reported (1). Since February 2021, India has been in the midst of the second wave of the pandemic, characterized by an exponential surge accounting for more than 60% of cases and deaths out of the cumulative 31 million cases and 400 thousand deaths in the country (2).

The spectrum of manifestations in COVID-19 varies widely and 80–90% of infections have been found to be asymptomatic in studies from Asia (3, 4). The global research map for COVID-19 developed by the World Health Organization (WHO) recommended population-level serological surveillance to provide robust estimates of infection and fatality rates (5, 6). This is particularly important in resource-constrained settings where viral diagnostic testing is inadequate and/or inaccessible and documentation of the diverse trajectory of SARS- CoV-2 infections is limited (7).

Rapid serosurveys in large cross-sections of the population have been conducted globally and in India, which have reported the changing burden of COVID-19 with the progress of the pandemic. (8–12). Nationwide seroprevalence rates in India increased from 0.7% in June 2020 to 67% in July 2021 (10, 13). While these studies have yielded some early and actionable evidence, they are limited in providing a comprehensive picture of viral transmission dynamics and host response in the population. Longitudinal cohort studies can overcome this limitation and help characterize the natural history of SARS-CoV-2 infection across different populations and geographical locations. An important correlate of protective immunity is the neutralizing capacity and durability of antibody response measured in longitudinal samples using plaque reduction neutralization test (PRNT) (14). These assays provide a quantitative measurement of the level of neutralizing antibodies and are considered the gold standard for measuring protective antibody levels (15). There is a paucity of information regarding the presence of neutralizing antibodies or duration of protection conferred by asymptomatic SARS-CoV-2 infections in the existing literature from population-based seroepidemiological studies in India.

Another gap in seroepidemiological studies from the Indian subcontinent is the under-representation of younger age groups. Pediatric infections are reported to be typically mild or asymptomatic, but dynamics of transmission, immune response and its neutralizing capacity in children is not clearly understood (16, 17).

Cohort studies in well-defined populations also offer an opportunity to assess the severity of the pandemic by enumerating the number of illnesses, hospitalizations, and deaths attributable to COVID-19 (18). A systematically designed and executed surveillance system can provide reliable information regarding these events in the study cohorts and complement the data obtained from serological surveys and epidemiological surveillance programmes.

The study protocol for a multi-site population-based cohort study to establish serial sero-surveillance and syndromic surveillance to monitor the trend of SARS-CoV-2 infection transmission in India is presented in this article. The study aims to estimate the cumulative annual incidence of SARS-CoV-2 infection based on four-monthly serial serosurveys, determine the risk factors for SARS-CoV-2 infection, hospitalization and deaths rates; and estimate the duration of persistence of antibodies in individuals with evidence of prior SARS-CoV-2 infection.

## Methods and Analysis

### Study Design

**Figure 1:**
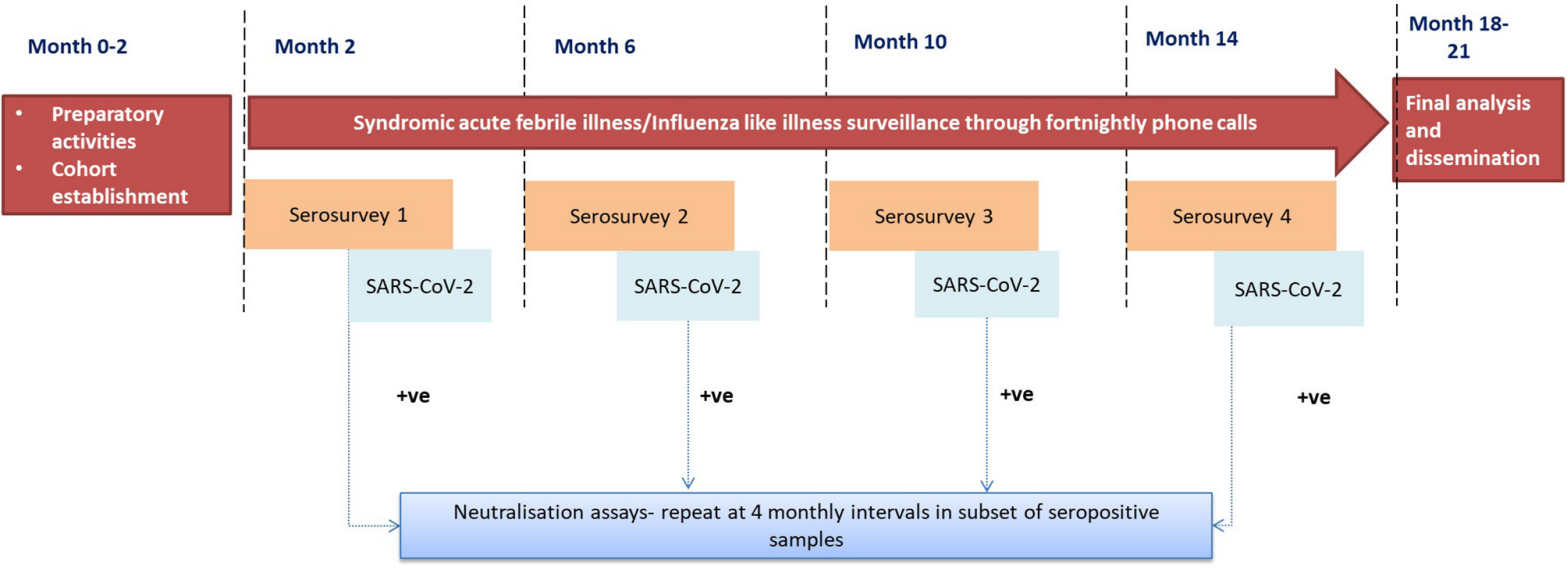
Study design.

This longitudinal study involves the follow-up of a cohort of individuals within specified geographical locations. The key activities (Figure 1) entailed in follow up would be:

A. Serial serosurveys at four-monthly intervals for SARS-CoV2.
B. Syndromic acute febrile illness (AFI)/ Influenza-like illness (ILI) surveillance through fortnightly phone calls.

### Study Sites

**Figure 2:**
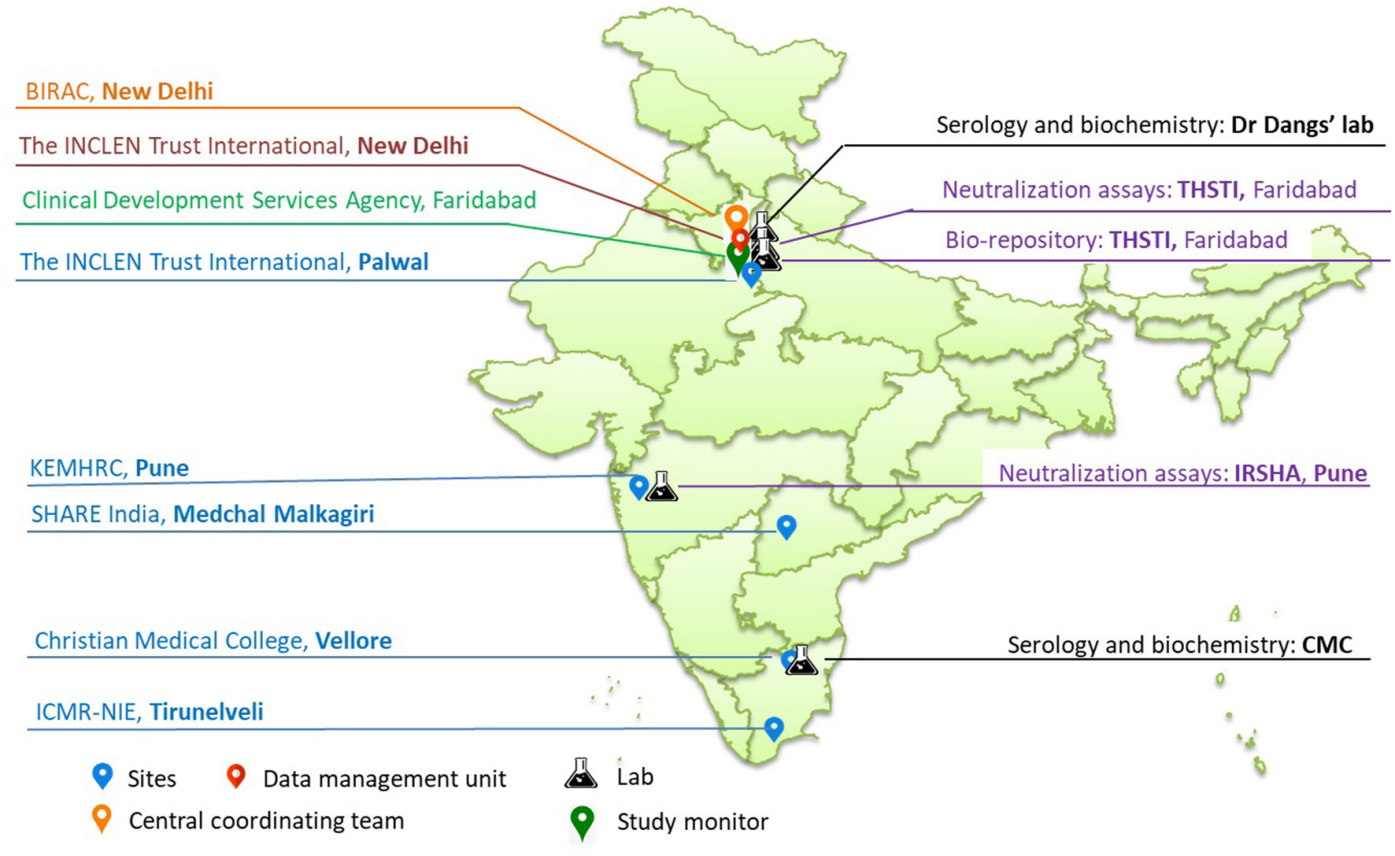
Study sites and laboratory network.

The study sites include five existing demographic surveillance sites from northern, western and southern states (also referred to as Demographic Health Surveillance sites-DHS)/Health and Demographic Surveillance Sites-HDSS)/Demographic, Developmental and Environmental Surveillance Sites-DDESS) of India (Figure 2), which are part of the clinical trial network (CTN) set up by the National Biopharma Mission (NBM)- Biotechnology Industry Research Assistance Council (BIRAC), Department of Biotechnology (DBT), Government of India (19). Of the five sites, four represent rural and one represents urban population. The socio-demographics at these sites are comparable to the larger population at the regional and national levels. As part of the CTN, these sites have been funded for developing infrastructure and capacity to enable them to undertake community-based trials for vaccines and therapeutics in compliance with good clinical practices (GCP). This CTN is referred to as DBT's Resource of Indian Vaccine Epidemiology Network (DRIVEN). The sites are supported by a central data management team from The INCLEN Trust International and laboratories are accredited by the National Accreditation Board for Testing and Calibration Laboratories (NABL), India (Figure 2). The study sites are:

A. Christian Medical College- The Vellore HDSS established in 2001 includes a population of around 150,000, covering 43 areas (16 urban wards and 27 rural areas) in the district of Vellore, Tamil Nadu.
B. Model Rural Health Research Unit (MRHRU) Tirunelveli is a flagship project of the Department of Health Research, Government of India, implemented through the Indian Council of Medical Research (ICMR). The MRHRU, Tirunelveli is linked to Tirunelveli Medical College and mentored by ICMR-National Institute of Epidemiology. MRHRU has established a rural DSS site comprising of 36,000 population from twelve villages in Mannur Block of Tirunelveli district, Tamil Nadu.
C. The INCLEN Trust International- The Trust established the SOMAARTH-DDESS in 2009, covering 200,000 individuals residing in 51 villages of district Palwal in Haryana.
D. KEM Hospital Research Centre (KEMHRC): KEMHRC established the Vadu HDSS in 2002, covering 160,000 individuals residing in 22 villages of Pune district in Maharashtra.
E. Society for Health Allied Research Education India (SHARE INDIA): The organization has established a geocoded cohort of 45,000 individuals residing in 40 villages as part of its Rural Effective Affordable Comprehensive Health (REACH) project in Medchal Malkagiri district of Telangana (20). The participants for this study will be recruited from clusters selected from the REACH project database.

#### Laboratory Network

The following laboratory networks support the cohort sites in the study:

1) Dr. Dang's Lab, situated in New Delhi will be responsible for estimation of glycosylated hemoglobin (HbA1C) and IgG antibody testing for SARS-Cov−2 for all the sites except Vellore.
2) Clinical Biochemistry and Clinical Virology laboratories of CMC Vellore will perform the estimation of HbA1C and IgG antibodies against SARS-CoV-2, respectively, for samples collected from Vellore.
3) Interactive Research School for Health Affairs (IRSHA), Pune, which has a BSL-3 facility, will be responsible for performing the PRNT assays for the samples received from Pune, Tirunelveli and Vellore.
4) Translational Health Science and Technology Institute (THSTI), Faridabad, with a BSL-3 facility, will perform the PRNT assays for samples received from Medchal and Palwal. The institute shall also host the centralized bio-repository for all leftover serum samples collected from all sites across all four rounds.

### Sample Size

The sample size for seroprevalence per site was calculated to be 5,000 per site, assuming 1.5% seropositivity, absolute precision of ±0.5%, confidence level of 95 and 10% loss to follow up (21). Based on the most recent site census, we expect the sample population to have the following age distribution (Table 1). A subset of seropositive samples from each round of serological survey will be selected for neutralization assays for SARS-CoV-2. Sample size for neutralization assay was calculated by considering the prevalence of neutralizing antibodies among the IgG seropositive participants as 85 with 10% absolute precision and 12.5% loss to follow-up in each round, serum samples of 75 SARS-CoV-2 IgG positive individuals from each round will be selected for PRNT assay from each site. In subsequent rounds, 75 new positives as well as follow-up samples of individuals who underwent PRNT in earlier rounds will be tested (Table 2).

**Table 1:** Site-wise age stratified recruitment completed at baseline.

**Age group**	**Palwal (*N* = 5,000)**	**Vellore (*N* = 5,100)**	**Medchal (*N* = 5,000)**	**Tirunelveli (*N* = 5,019)**	**Pune (Vadu) (*N* = 5,003)**	**Total (*N* = 25,211)**
2–8 years	996 (20%)	530 (10.4%)	522 (10.3%)	406 (8%)	483 (9.6%)	2,937
9–17 years	1,122 (22%)	981 (19.2%)	873 (17.2%)	816 (16%)	746 (14.9%)	4,538
18–59 years	2,467 (49%)	3,104 (60.9%)	3,186(62.6%)	3,150 (63%)	3,154 (63.0%)	15,061
≥60 years	416 (8%)	485 (9.5%)	507(9.9%)	647 (13%)	620 (12.3%)	2,675

**Table 2:** Key data points and investigations for sero-surveys.

	**Baseline (Round 1)**	**Month 4 (Round 2)**	**Month 8 (Round 3)**	**Month 12 (Round 4)**
Socio-demographic information	X			
Medical history (comorbidities, medications)	X	X	X	X
Immunization history (including COVID-19 vaccination)	X	X (only COVID-19 vaccination)	X (only COVID-19 vaccination)	X
Anthropometry (height, weight, body mass index)	X	X	X	X
Vitals (blood pressure, temperature, oxygen saturation)	X	X	X	X
**Basic biochemical investigations**				
Hemoglobin	X	X	X	X
Glycosylated hemoglobin	X (≥18 years)			
Random blood sugar		X (≥18 years)	X (≥18 years)	X (≥18 years)
**Serological assays**				
Estimation of IgG antibodies against SARS-CoV 2	X	X	X	X
SARS-CoV-2 Neutralization assays	X subset of SP^a^ samples (*n* = 75)	X subset of SP^a^ samples of R1 (*n* = 75) and R2 (*n* = 75)	X subset of SP^a^ samples of R1, R2 (*n* = 150) and R3 (*n* = 75)	X subset of SP^a^ samples of R1, R2, R3 (*n* = 225) and R4 (*n* = 300)

a *SP, Seropositive per site*.

### Sampling Method and Selection of Participants

A cluster sampling method will be followed wherein a village or ward will be considered a cluster in rural and urban sites, respectively. Clusters at the Vellore study site are urban, while those at the other four study sites are rural.

Out of all clusters listed at the site, five clusters will be chosen purposively so that the entire site area is well-represented. In each cluster, contiguous households will be approached. All individuals above 2 years of age and currently residing and are likely to stay till the end of study (1 year) in the study area will be considered eligible for inclusion. Since individuals who are unlikely to be available for 1 year will not be recruited, delays in the timely completion of serosurvey due to non-availability of participants is expected to be minimal. Individuals with diagnosed psychiatric illness and intellectual disability will be excluded. A household will be enrolled only if 50% or more of its eligible members consent to participate in the study.

### Serial Serosurveys

A baseline round of serosurvey (Round 1) will be conducted among all asymptomatic participants. Serum samples collected from serosurveys will be tested for IgG antibodies against SARS-CoV-2 (details in subsequent sections). After completing the baseline serosurvey, enrolled members of the cohort will undergo three more rounds of serosurveys at 4, 8, and 12 months. A brief questionnaire and temperature monitoring will be used to identify the suspected cases among the study participants. Data collection from a household with confirmed and suspected cases will be deferred for 21 days, and efforts will be made to ensure that such suspected cases are mobilized to the nearest COVID-19 care center.

#### Clinical and Socio-Demographic Data

Basic socio-demographic data, medical history and vaccination history including COVID-19 will be recorded. In addition, height, weight, mid-upper arm circumference, oxygen saturation level for all the participants, and blood pressure for adults (18 years and above) during each round of the serosurvey will be measured.

#### Blood Sample Collection and Processing

Blood samples will be collected in serum separator tubes for the serological tests. For adults, in the first round, an additional 2 ml of blood will be collected in EDTA tubes for measuring HbA1C levels. Blood volumes required to be collected for serology are age-dependent, i.e., 4 ml in children and 6 ml in adults.

Serum will be separated at the nearest health facility/ field site lab within 24 h of collection by centrifuging the samples at 3,000 RPM for 10 min. For each sample collected, three aliquots of serum will be prepared in the order of priority. The first aliquot will have 400 microliters for SARS-CoV-2 IgG testing within 72 h of sample collection. The second aliquot will have 800 microliters for performing PRNT assays in a selected subset of samples. The third aliquot will have the leftover serum for archival at −80°C in the biorepository.

#### Basic Investigations

A few investigations will be carried out for the benefit of the participants such as HbA1C for adults in round one of serosurvey using the high-performance liquid chromatography (HPLC) method. The result of HbA1C test will be provided to the participants within a week of blood sample collection. In the subsequent rounds Random Blood Sugar will be tested using a digital glucometer. Hemoglobin will be tested in all participants, using a point of care digital hemoglobinometer (Hemacue 201^®^). The results of these investigations will be given to the participants immediately.

#### Serological Assays

IgG antibodies against SARS CoV-2 will be detected using Diasorin Liaison^®^ SARS-CoV-2 S1/S2 IgG, a chemiluminescence immunoassay (CLIA)- which detects antibodies against the S1/S2 subunits of viral spike protein (22).

#### Neutralization Assay

For PRNT assay, 75 samples will be proportionally selected based on the percentage of seropositivity among the four age categories (2–8, 9–17, 18–59, and ≥60 years). Neutralizing antibody titers will be defined as the highest serum dilution that results in ≥90% (PRNT90) reduction in the number of virus plaques (23, 24).

### Syndromic Acute Febrile Illness/Influenza Like Illness Surveillance Through Fortnightly Phone Calls

During recruitment, all households in each site will be provided with a digital thermometer to record and monitor temperature in case any family member feels unwell, and also a helpline contact number for reporting any such febrile episodes to the study team members. Trained tele-callers shall make fortnightly phone calls to all the participating households to collect details about any symptoms suggestive of acute febrile illness, especially COVID-19, in the previous 2 weeks among the enrolled members. The details of symptoms, including the date of onset, date of RT-PCR test for SARS-CoV-2 with the result, treatment and outcome, will be recorded on standard case report forms (CRF) during the fortnightly calls.

If any participant reports a history of fever of more than 2 days, the entire household will be followed up over the phone at a weekly interval for 21 days. The field team will be informed regarding the case to facilitate their referral to the nearest COVID-19 diagnostic center. Treatment records (if available) will be accessed to record the diagnosis assigned to the AFI event. Any hospitalization event reported during the calls will be recorded, and attempts will be made to extract the details of the diagnosis, investigations including RT-PCR, course of hospitalization and treatment received from hospital records or discharge summaries. If required, the study teams shall make hospital and home visits to confirm this information. Prior permission will be sought from the hospitals in the catchment area of the sites for accessing hospital data in case of any febrile hospitalization. Teams at the study sites are also involved in supporting the delivery of public health services at their respective locations. This enduring rapport between the sites and the community will be leveraged to seek and secure relevant medical records as per the study protocol.

### Verbal Autopsy for Deaths in the Cohort

All deaths in the cohort will be recorded, irrespective of the cause. The site team will attempt to retrieve information on the cause of death. Wherever a medical certificate for the cause of death is available, the cause will be recorded as per the certificate. Additionally, the team will carry out a verbal autopsy (VA) based on the 2016 WHO Verbal Autopsy Instrument (25). A cause of death will be assigned with the data collected using the VA questionnaires and any other available information by a trained physician. At least two physicians will be trained in VA coding and will independently assess individual questionnaire data, and the assignment of cause of death will be done by a consensus review or by a third physician in case of a dispute. These causes will be mapped to the International Statistical Classification of Diseases and Related Health Problems, for standardization.

### Data Management

**Figure 3:**
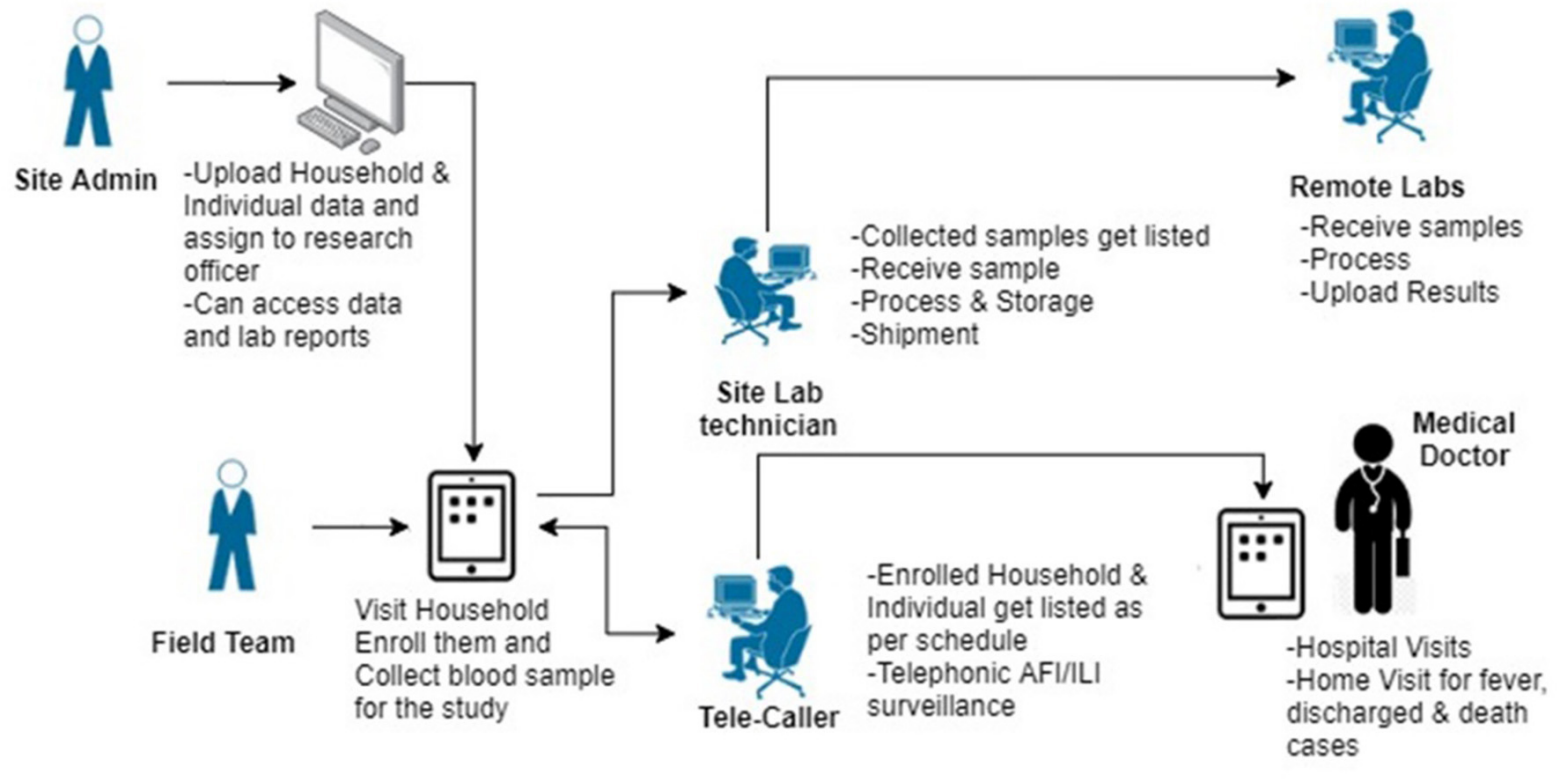
Overview of the SOMAARTH-3 electronic data capture platform.

Data collection will be done on a paperless data management platform (SOMAARTH-3) developed by the central data management team. The web and android based platforms have been designed to enable automation of study processes with in-built logic checks and role-based access control (Figure 3). Notable features of the platform that are specific to this study include

1. Harmonized interface for importing the sampling frame from the sites' existing population enumeration database.
2. Household based enrolment and nesting of individuals within a household for all study processes.
3. Web-based tele caller module with auto-generation of call listings based on pre-defined follow-up intervals.
4. Web-based lab module for sample management and provision of reports for the site as well as central labs.
5. Separate platform (Jira^®^) for reporting, tracking and resolution of any issues that develop during the course of data collection for the project.

The algorithms describing study processes and CRFs used in this study are provided in Supplementary Files 1, 2, respectively.

### Analysis Plan

The seroprevalence of SARS-CoV-2 infection will be estimated from the baseline survey. The proportion of positives among the seronegatives tested and the seroconversion rates will be used for estimating the incidence of infection at 4, 8, and 12 months. Cumulative incidence/ incidence density will be calculated at the end of 1 year. All estimates will be calculated with 95% confidence interval. Household and individual-level risk factors associated with COVID-19 infection/ disease will be identified using regression models. Sequential neutralization antibody titres among sero- positives will help to analyze the duration of persistence of antibodies.

### Expected Outcomes

Key expected outcomes of the study include:

- Age-specific attack rate: The proportion of individuals per age strata who show seroconversion between consecutive two rounds.
- Age-specific cumulative incidence of asymptomatic infections: The proportion of individuals per age strata who show seroconversion over 1 year but do not report any symptoms suggestive of the SARS-CoV-2 infection during AFI/ILI surveillance.
- Age-specific cumulative incidence of symptomatic events: The proportion of individuals per age strata identified as suspected cases during AFI/ILI surveillance.
- Symptomatic proportion of cases (symptomatic fraction): The proportion of individuals who show symptoms or signs of SARS-CoV-2 infection during AFI/ILI surveillance.

### Quality Assurance

The study will be implemented as a collaborative partnership with investigators of all five sites and the central data management and coordination teams. The study protocol, standard operating procedures and tools have been developed based on feedback and consensus generated via a consultative process, with participation of site investigators and subject experts. Each site has also conducted on-field pilot testing of the study CRFs and the EDC, which has enabled iterative refinement of these tools based on scientific reasoning and operational feasibility.

Since restrictions imposed due to the pandemic precluded in-person large group training, a centralized 4-day training of trainers (ToT) was conducted. The ToT was attended by 4–5 key study personnel from each site, which included one study investigator, site coordinator, data manager and laboratory coordinator. The ToT had hands-on training on using the EDC and demonstration of laboratory procedures. The trainers from each site subsequently trained the on-site research teams according to the training plan implemented in the ToT. Each site has access to a standardized set of resources, which include; CRF instructions, operational manuals, training presentations and videos.

The network partners will meet once a month over virtual platforms to discuss study progress, key findings and operational challenges. The progress of the study will be monitored regularly by an independent scientific advisory group of experts designated by the funding agency. The Clinical Development Services Agency (CDSA) has been identified as an external study monitor and ensures adherence to GCP in study implementation through regular virtual and on-site visits. The central data management team will conduct a periodic review of the data and resolves data discrepancies in consultation with the site data management team. A lab expert committee has been formed, which was responsible for harmonizing lab processes across sites (Supplementary File 3) and will be conducting real-time QA for these processes starting from sample collection till the reporting of results, following a set of pre-determined indicators.

## Discussion

The long-standing community presence and experience of maintaining population cohorts in the selected study sites are being leveraged to fill knowledge gaps related to the actual burden of COVID-19 in the community and its outcomes. The sites represent a mix of rural and urban populations and present an opportunity to estimate the COVID-19 disease burden in both settings. The design of the survey will enable study of viral dynamics at the community, household and individual level, due to the inclusion of entire households located in contiguity within a cluster. The survey will include children above 2 years of age and thus provide representative and adequate data for children. In most sites, round 1 of the serosurvey would coincide with the peak of the second wave and the launch of mass COVID-19 vaccination drives. Subsequent rounds in the same cohort will help improve our understanding of community transmission and immune response to vaccination. Serial serosurveys at four-monthly intervals will also prove to be valuable for capturing any seasonal variations and trends in the infection due to variations in the circulating virus; determined through ongoing genomic surveillance in the country. The use of validated assays for antibody detection and neutralization testing add to the internal validity of the study.

The telephonic AFI/ILI surveillance model established in this study has the potential for being scaled up for use in surveillance of adverse events following immunization as part of clinical trials or vaccination drives and also for the surveillance of other infectious diseases. Well-characterized cohorts and the associated bio-repository can lend themselves to exploration of other infectious disease etiologies without much incremental cost.

Operational challenges unique to the COVID-19 pandemic, in the form of interruptions due to lockdowns, restrictions in field mobility in containment zones and outbreaks in the study teams are anticipated in the study. Hesitance on the part of potential participants toward home based data collection and a guarded attitude toward disclosing symptoms of febrile illnesses due to COVID associated stigma is expected in some communities (26). Each site has mitigation strategies in place which include factoring in buffer time between serosurveys, training and adherence to COVID-19 appropriate behavior, vaccination of research staff, availability of reserve field teams and structured stakeholder engagement strategies.

There are a few limitations to this study. Accurate estimates of the incidence of COVID-19 “disease” will not be available within this study design because of the challenges of conducting COVID-19 confirmatory tests (RT-PCR on naso/oro-pharyngeal swabs) in symptomatic individuals identified during syndromic surveillance in community settings. For the same reason, intrahousehold transmission dynamics in terms of secondary attack rates, will not be available. We expect that functional linkages with the local health system will be established at each study site so that confirmed cases in the cohort are intimated to the research team, for reasonable estimation of the incidence of symptomatic infections.

Antibody cross-reactivity in SARS-CoV-2 with dengue has been reported (27, 28). Most of these reports are based on dengue and/ SARS-CoV-2 rapid tests that are qualitative and lack a high level of specificity. Diasorin Liaison^®^ SARS-CoV-2 S1/S2 IgG assay used in the present study is a quantitative CLIA with high specificity (>97%) (29). However, false-positive results may occur at lower levels and possibly due to cross-reactivity from pre-existing antibodies or other causes (30). The neutralization assay to be used in this study was developed using the Wuhan strain. Neutralization against other strains will not be possible in view of budgetary constraints associated with testing for multiple variants that have and may emerge over time. The samples archived in the bio-repository could be analyzed using a meso-scale discovery platform allowing testing against all the available variants using ACE-receptor-based assays, if additional funding is secured.

In conclusion, this population-based seroepidemiological study will help determine the burden of COVID-19 at the community level in urban and rural Indian populations and guide in monitoring the trends in the transmission of SARS-CoV-2 infection.

## Ethics Statement

This study has been approved by the institutional Ethics Committee of all participating institutions and will be conducted as per the National Ethical Guidelines for Biomedical and Health Research involving Human Participants issued by the Indian Council of Medical Research in 2017 and the more recent National Guidelines for Ethics Committees Reviewing Biomedical & Health Research During COVID-19 Pandemic issued in 2020 (31, 32). All participants read and sign the informed consent or assent form (as applicable), according to the National Ethical Guidelines for Biomedical and Health Research involving Human Participant issued by the Indian Council of Medical Research in 2017. A copy of the signed consent form is given to the participant.

### Report Distribution to Participants

The serological tests proposed in this study are not diagnostic tests and do not differentiate between recent or past infection. The results will be disclosed to the participants as and when they are available. It will be explained very clearly to the participant at the time of consent, testing and reporting that these are not diagnostic tests to avoid creating panic and stigma.

### Referral of Participants

Any participant found to have symptoms suggestive of COVID-19 will be advised to visit the designated local health facilities and the research team will immediately facilitate this process. Any participant found to have abnormal blood pressure, hemoglobin or HbA1C will be provided by a referral slip and advised to consult a physician for further management.

### Safety of Study Team During Field Work

Research staff will be provided appropriate personal protective equipment (N-95 and 3-ply masks, faceshield, gloves, and apron) and infection control modalities (alcohol-based hand sanitizers) to be used at the time of blood sample collection.

### Dissemination

Key results of the study shall be made available in the form of pre-prints prior to peer-review so as to inform local and global epidemiological studies. The final report will be submitted to national agencies involved in the management of the pandemic (Ministry of Health & Family Welfare, and Department of Biotechnology, Ministry of Science and Technology, Government of India). The findings of the study will be published in peer-reviewed journals and presented in scientific meetings and conferences.

## Ethics Statement

This study was approved by the Institutional Ethics Committee of all participating institutions - Christian Medical College, Vellore (EC reference number: 13274), ICMR-National Institute of Epidemiology, Chennai (EC reference number: NIE/IHEC/202009-01), The INCLEN Trust International, New Delhi (EC reference number: IIEC068), KEM Hospital Research Center, Pune (EC reference number: KEMHRC/RVM/EC/2183), and Society for Health Allied Research and Education, India (EC reference number: EC/28/VIII/2K20(3/7). The study was conducted following National Ethical Guidelines for Biomedical and Health Research involving Human Participants, 2017 and National Guidelines for Ethics Committees Reviewing Biomedical & Health Research During COVID-19 Pandemic issued in 2020 (31, 32). All participants read and signed the informed consent or assent form (as applicable). A copy of the signed consent form was given to the study participants.

## DRIVEN Team

Kalyani Thakur, Samridhi Ranjan, Neeraj Kashyap, Abhishek Agarwal, Shikha Dixit, Meenakshi Bakshi, M Ravi, K Sunitha, M Santhosh Kumar, K Shantaraman, Jacob John, Venkata Raghava Mohan, Kulandaipalayam Natarajan Sindhu, Asha Abraham, Mahesh Moorthy, Anuradha Rose, Joseph Jovin Stanley, Pamela Christudoss, Shikha Dhawan, Vidya Arankalle, Dhiraj Agarwal, Harshpreet Kaur, Rutuja Patil, Ashish Bavdekar.

## Author Contributions

DN, RR, SR, PR, and SMM conceptualized the study and drafted the study protocol. DN, PR, RR, SD, and BK developed the study tools. RS, SMM, and KS established the DRIVEN study network. GC, NA, WR, SJ, GJ, YJ, KS, and SMe revised the tools and protocol. The DRIVEN team as a whole was involved in developing study algorithms and processes for the electronic data collection platform. All authors contributed to manuscript revision, read, and approved the submitted version.

## Funding

This study was funded by National Biopharma Mission and Grand Challenges India, Biotechnology Industry Research Assistance Council (BIRAC), a non-profit public sector enterprise set up by the Department of Biotechnology (DBT), Ministry of Science and Technology, Government of India - Christian Medical College, Vellore (BIRAC/BT/NBM0205/05/19BCX1XD), ICMR-National Institute of Epidemiology, Chennai (BIRAC/BT/NBM0206/05/19BCX1XD), The INCLEN Trust International, New Delhi (BIRAC/BT/NBM0253/05/19BCX1XD), KEM Hospital Research Center, Pune (BIRAC/GCI/2020/COVID-19/Serosurveillance/KEMVadu), and Society for Health Allied Research and Education, India (BIRAC/BT/NBM0203/05/19/BCX1XD).

## Conflict of Interest

The authors declare that the research was conducted in the absence of any commercial or financial relationships that could be construed as a potential conflict of interest.

## Publisher's Note

All claims expressed in this article are solely those of the authors and do not necessarily represent those of their affiliated organizations, or those of the publisher, the editors and the reviewers. Any product that may be evaluated in this article, or claim that may be made by its manufacturer, is not guaranteed or endorsed by the publisher.

## Abbreviations

AFI: Acute Febrile Illness
BIRAC: Biotechnology Industry Research Assistance Council
BSL-3: Bio-Safety Level-3
CDSA: Clinical Development Services Agency
CLIA: Chemiluminescence Immunoassay
CMC: Christian Medical College
COVID-19: Coronavirus Disease-2019
CRF: Case Report Forms
CTN: Clinical Trial Network
CTRI: Clinical Trial Registry of India
DBT: Department of Biotechnology
DDESS: Demographic, Environment and Developmental Surveillance Site
DHS: Demographic and Health Surveillance Site
DRIVEN: DBT's Resource of Indian Vaccine Epidemiology Network
DSS: Demographic Surveillance Site
EDC: Electronic Data Capture
EDTA: Ethylenediaminetetraacetic Acid
GCP: Good Clnical Practice
HDSS: Health and Demographic Surveillance System
HPLC: High-performance Liquid Chromatography
ICMR: Indian Council of Medical Research
ILI: Influenza like illness
IRSHA: Interactive Research School for Health Affairs
KEMHRC: KEM Hospital Research Centre
MRHRU: Model Rural Health Research Unit
NABL: National Accreditation Board for Testing and Calibration Laboratories
NBM: National Biopharma Mission
NIE: National Institute of Epidemiology
PRNT: Plaque Reduction Neutralisation Tests
RBS: Random Blood Sugar
REACH: Rural Effective Affordable Comprehensive Health
RT-PCR: Reverse Transcription Polymerase Chain Reaction
SARS CoV-2: Severe Acute Respiratory Syndrome Coronavirus 2
SHARE: Society for Health Allied Research Education
THSTI: Translational Health Science And Technology Institute
VA: Verbal autopsy
WHO: World Health Organization.
