# Supplementary material for: Sero-Surveillance to Monitor the Trend of SARS-CoV-2 Infection Transmission in India: Study Protocol for a Multi Site, Community Based Longitudinal Cohort Study
Source: Front Public Health. 2022 Mar 24;10:810353. doi: 10.3389/fpubh.2022.810353 (PMC8987192; doi:10.3389/fpubh.2022.810353)

Fig S1a: Household level processes for assessing eligibility

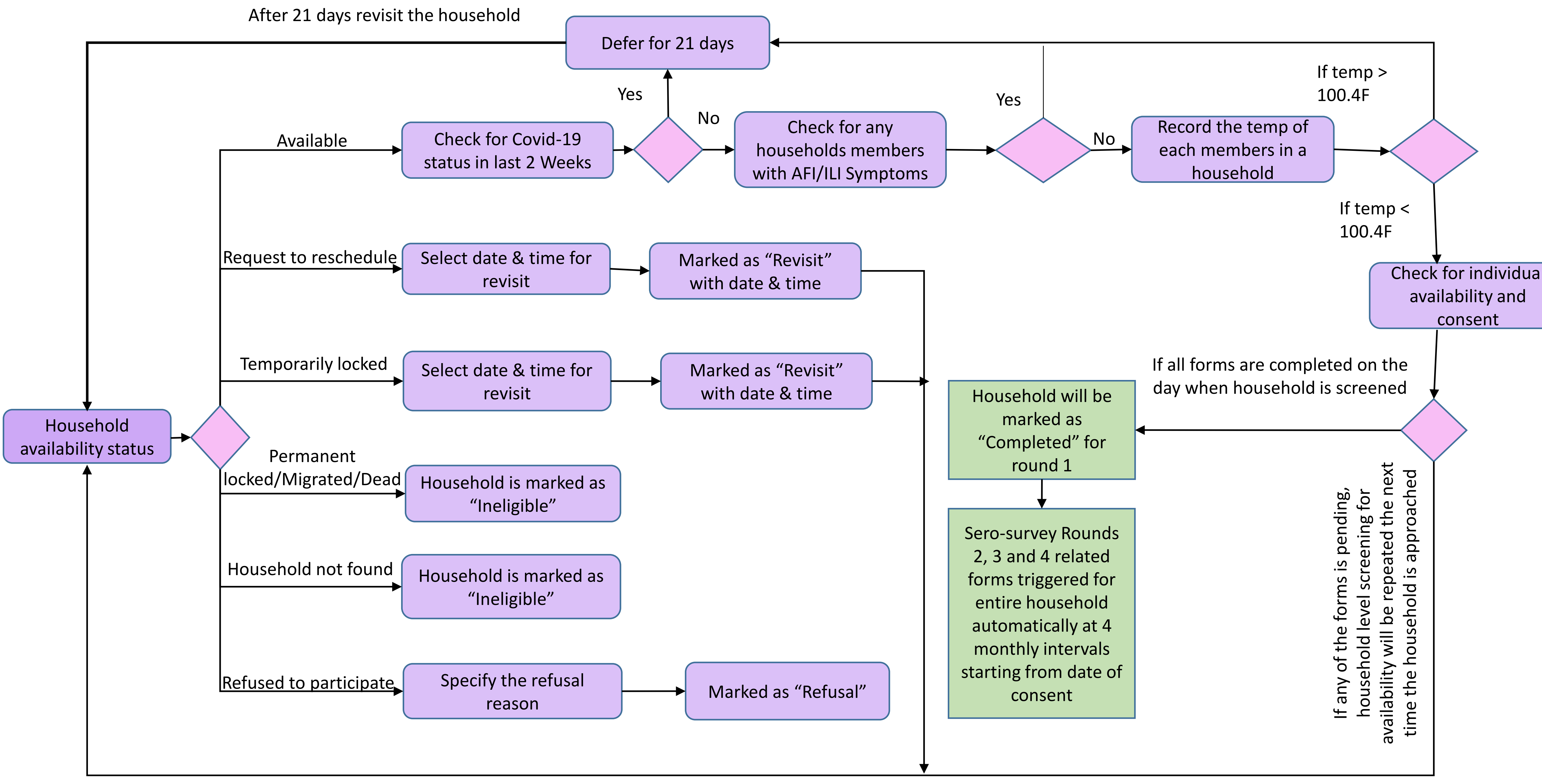

Fig S1b: Individual level processes for assessing eligibility

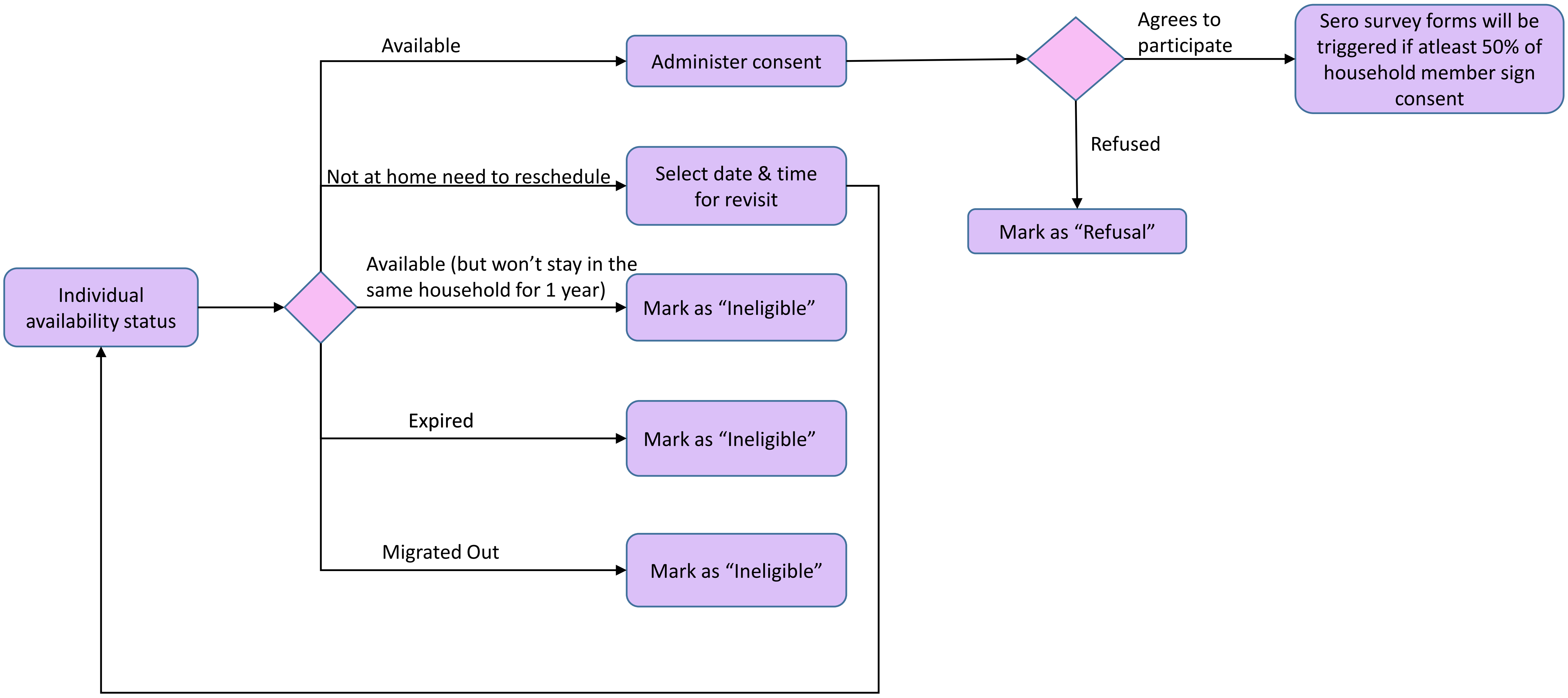

Fig S1c: Phone call based AFI/ILI surveillance process

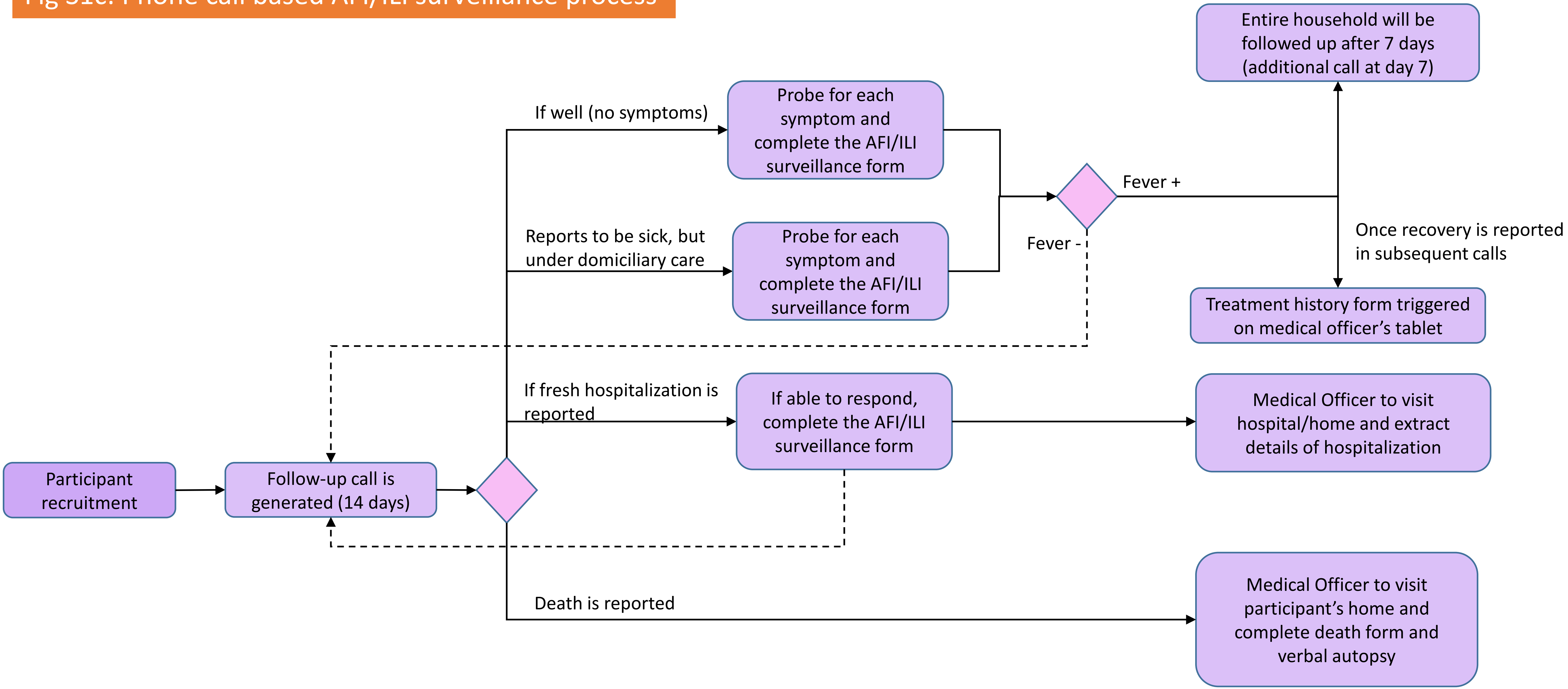

Fig S1d: Follow up of hospitalised cases

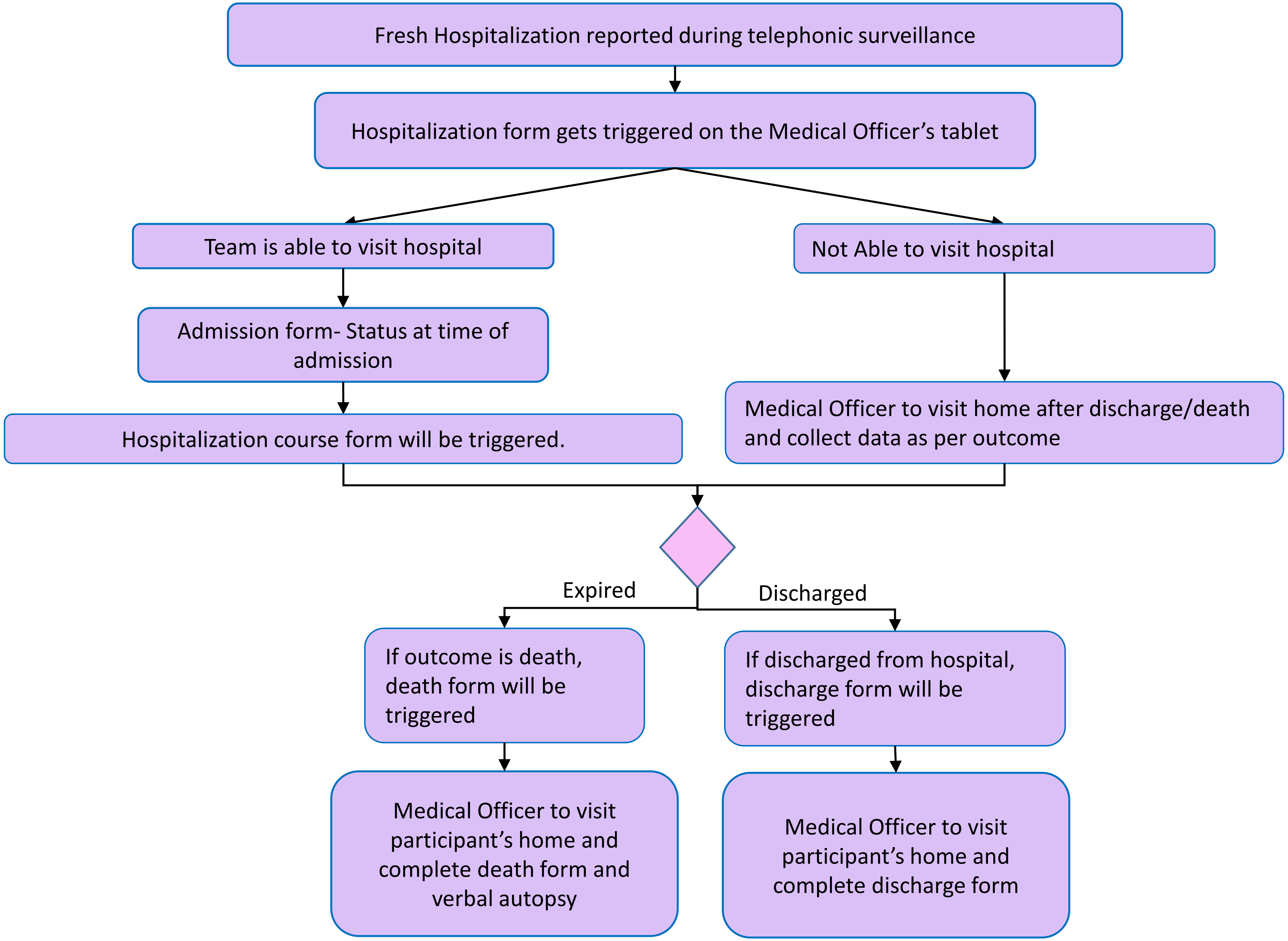

Supplement: Supplementary file 1 [file Data_Sheet_1.PDF]
