# Supplementary material for: Sero-Surveillance to Monitor the Trend of SARS-CoV-2 Infection Transmission in India: Study Protocol for a Multi Site, Community Based Longitudinal Cohort Study
Source: Front Public Health. 2022 Mar 24;10:810353. doi: 10.3389/fpubh.2022.810353 (PMC8987192; doi:10.3389/fpubh.2022.810353)
Supplement: Supplementary file 2 [file Data_Sheet_2.pdf]

Table S2a: List of CRFs

CRF	CRF No:	CRF Name	Description
1	CRF0A	House hold listing	Key sociodemographic variables to be filled for potential households for recruitment grouped according to cluster, loaded on to SOMAARTH 3 prior to initiation of study. Section A is household level information and section B is individual level information
2	CRF0B	Individual listing	Key sociodemographic variables to be filled for potential households for recruitment grouped according to cluster, loaded on to SOMAARTH 3 prior to initiation of study. Section A is household level information and section B is individual level information
3	CRF1A	Household consent form and Individual consent form	Record status of consent at HH level and in section B list of all household members who have given consent for the study
4	CRF1B	Household socioeconomic scale	Variables as for Wealth Index calculation as per NFHS 5 questionnaire
5	CRF3B	AFI/ILI surveillance form or follow up call	Symptoms of AFI/ILI, contact, travel and social history to be enquired every follow up<br>-Whole HH is asymptomatic (during regular fortnightly calls)<br>-Index participant is symptomatic or somebody in the HH is symptomatic (frequent follow ups of HH contacts (7,14,28) in case on symptoms in any HH member)<br>Subsequent CRFs 4A,4B and 4C will be triggered based on this.
6	CRF4A	Treatment History	To be filled for individuals who report to have recovered from symptoms since last contact. Information may be collected by home/hospital visit or over phone call. Records the treatment history and health seeking behaviour
7	CRF4B.1	Hospitalization history at admission	To be filled for a patient who is hospitalized (admission related information)
8	CRF4B.2	Hospitalization course and investigation	Follow up of hospitalized participants- clinical course and investigations
9	CRF4B.3	Hospital discharge	To be filled for a patient who are discharged from the hospital
10	CRF4C	Death Form	To be filled in for any death in the cohort (in addition to verbal autopsy

			instrument)
11	CRF5A	Serosurvey sociodemographic information	To be filled for the participant where it includes information related to socio-demography
12	CRF5B	Immunization history	To be filled for the participant where it includes the immunization history details for the past 1 year.
13	CRF5C	Serosurvey history and examination	This form is in two sections where section 1 includes the details related to the history of any condition diagnosed and section 2 includes the vital measurement of the participant measured on the day of visit
14	CRF5D.1	Sample Collection in Serum Separator Tube	Sample collection details of the participants
15	CRF5D.2	Sample Collection in EDTA tube	Sample collection details of the participants
16	CRF5D.3	Point of Care Tests	Sample collection details of the participants

Form	Q No	Questions	Response options	Instruction to Data collector	Built in software logics
**Household listing (extracted from site's demographic surveillance database)**					
CRF0A	1	Household ID	NA	Enter the ID that uniquely identifies a household in your site's database. The format may be text/ numeric/ alphanumeric/ with or without special characters	None
CRF0A	2	Land Parcel ID (Local Census ID)	NA	Enter the ID from your site's database that uniquely identifies the plot of land/location in which the household resides.	None
CRF0A	3	Latitude	NA	Enter the latitude of land/location in which the household is situated. Eg 28.6448	None
CRF0A	4	Longitude	NA	Enter the longitude of land/location in which the household is situated. Eg. 77.217	None
CRF0A	5	Household Head Name	NA	Enter the full name of the head of the household. No restrictions on length of name of characters.	None
CRF0A	6	Age Household Head	1|Male<br>2|Female<br>3|Third Gender	Enter the numeric age in completed years.	None
CRF0A	7	Gender Household Head	NA	Enter the gender of the head of the household	None
CRF0A	8	Household Head Father Name	NA	Enter the head's father's full name	None
CRF0A	9	Household Head Mother Name	NA	Enter the head's mother's full name	None

CRF0A	10	Household Head Spouse Name	NA	Enter the head's spouse's full name	None
CRF0A	11	Address/ Name of building/ House number/ Street/ Mohalla/ Nearest landmark	NA	Enter the detailed address of the household which may be useful for the field team to reach the house easily.	None
CRF0A	12	Caste	1|General<br>2|Scheduled Caste<br>3|Scheduled Tribe<br>4|Other Backward class<br>99|Don't know	Enter the caste which the household belongs to from one of the response options. Enter Don't know if this detail is not available.	None
CRF0A	13	Religion	1|Hindu<br>2|Muslim<br>3|Christian<br>4|Sikh<br>5|Buddhist/Neo- Buddhist<br>6|Jain<br>7|Jewish<br>8|Parsi/Zorastrian<br>9|No religion<br>99|Don't Know	Enter the caste which the household belongs to from one of the response options. Enter Don't know if this detail is not available.	None
CRF0A	14	Date on which this Household observation/ update was last updated in the sites' database	NA	Enter the date on which this data was last updated. Date should be entered in dd/mm/yyyy format	Existing Logic: Date should not be future date
CRF0A	15	Contact Number	NA	Enter one contact number for the household. Enter only numbers without any space or special characters	None

Individual listing (extracted from site's demographic surveillance database)					
CRF0B	1	Individual ID	NA	Enter the ID that uniquely identifies an individual in site's	None
CRF0B	2	Household ID	NA	Enter the ID that uniquely identifies a household in your site's database.	None
CRF0B	3	Full name	NA	Enter the full name of the individual. No restrictions on length of name of characters.	None
CRF0B	4	Date of birth	NA	If DOB is available in database, please provide DOB in dd/mm/yyyy format. If DOB is not available:<br>1. Calculate DOB based on age as existing site-specific guidelines<br>2. If site specific guidelines are not present, calculate year of birth based on age and enter DOB as 1st July of that year.	Existing Logics: should not be a future date
CRF0B	5	Is the DOB as reported by participant or had to be derived (back calculated) from age?	1|As Reported<br>2|Derived	Enter "As reported" if DOB was available in database. Enter "Derived" if DOB was calculated as per age. Enter "Not Available" if you are not sure if the DOB in your database is as reported or was derived from age	None
CRF0B	6	Gender	1|Male<br>2|Female<br>3|Third Gender	Enter the gender of the individual	None

CRF0B	7	Father's Name	NA	Enter the individual's full father's name	None
CRF0B	8	Mother's Name	NA	Enter the individual's full mother's name	None
CRF0B	9	Marital status	1|Currently Married<br>2|Married but gauna not performed<br>3|Widowed<br>4|Divorced<br>5|Separated<br>6|Deserted<br>7|Never married<br>99|Unknown	Enter marital status form one of the options provided here	Existing logic:<br>if response is "never married, then 10 will be skipped
CRF0B	10	Spouse Name	NA	Enter the individual's spouse's full name	

CRF0B	11	Relationship with Head of Household	1|Self 2|Husband<br>3|Wife 4|Father<br>5|Mother 6|Brother<br>7|Sister 8|Son<br>9|Daughter 10|Step father<br>11|Step Mother<br>12|Step Brother<br>13|Step Sister<br>14|Son-in-Law<br>15|Daughter-in-Law<br>16|Grandson<br>17|Granddaughter<br>18|Great Grandson<br>19|Great Granddaughter<br>20|Father-in-Law<br>21|Mother-in-Law<br>22|Brother-in-Law<br>23|Sister-in-Law<br>24|Nephew 25|Niece<br>26|Uncle 27|Aunt<br>28|Adopted Child<br>29|Grandfather<br>30|Grandmother<br>31|Cousin<br>32|Domestic Servant<br>97|No relation<br>98|Other Relatives<br>99|Dont Know	Choose and enter the relationship of the individual with head of household from one of the response options given here.	

CRF0B	12	Last date on which this information was last updated	NA	Enter the date on which this data was last updated. Date should be entered in dd/mm/yyyy format only. Make sure the cells are formatted to date fields.	Date should not be a future date

Form	Q No	Questions	Response options	Instruction to Data collector	Built in software logics
**House Hold Registration and Enrollment**					
CRF1A	*1*	*Status of Household*	1|Available<br>2|Request to reschedule<br>3|Temporary Locked (Reschedule)<br>4|Permanent Locked<br>5|Migrated/Dead<br>6|Household not found<br>7|HH planned to migrate out in next 1 year<br>8|Refused to participate	check the availability status of the household	Existing logic:<br>"If response is 1, it asks the next question.<br>If response is 2 and 3, requested rescheduled date and time of visit should enter.<br>If response is 4,5,6, Form will end here.<br>If response is 8, write the reason for refusal.
CRF1A	*2*	*Was anyone in the household diagnosed with Covid-19 in the two weeks preceding the date of survey?*	1|Yes<br>2|No		Existing Logic: If "Yes", Deferred to 21 days.
CRF1A	*3*	*Does any member of the household have fever on the day of contact?*	1|Yes<br>2|No		Existing Logic: If "Yes", Deferred to 21 days.
CRF1A	*4*	*Record the temperature of each member of the household using infrared thermometer. Did anu member have a temperature above 100.4 F by thermometer reading*	1|Yes<br>2|No	Measure the temperature of each member of the household	Existing Logic: If "Yes", Deferred to 21 days.

Form	Q No	Questions	Response options	Instruction to Data collector	Built in software logics
**CRF 1A_Section B: Individual availability status (List of all household members who have given consent for the study)**					
CRF1A	*1*	*Status of individual at a time of visit*	1|Available<br>2|Not at home need to reschedule<br>3|Expired<br>4|Migrated out<br>5|No contact even after 3 attempts		If response is 1, go to next question<br>If response 2, then enter the date and time of revisit<br>If response is 3,4,5 close and exit and exclude from individual list
CRF1A	*2*	*Is individual eligible for study*	1|Yes<br>2|No		If response is No, fill reason in Q3, move to ineligible and exit
CRF1A	*2.1*	*If No. Mention reason for ineligibility*	1|May not stay in same HH for 1year<br>2|Diagnosed psychiatric illness or mental retardation		NA
CRF1A	*2.2*	*If Eligible, was consent obtained from the individual*	1|Signed<br>2|Refused		If response is "signed" go to 4.<br>if response is "Refused "go to 3
CRF1A	*3*	*If refused, specify reasons*	1|Not interested in study<br>2|Not willing to undergo blood sampling<br>3|Not willing to undergo phone-based surveillance<br>4|Others		NA
CRF1A	*4*	*IF signed, then date of consent*	NA		Should not be a future date

Form	Q No	Questions	Response options	Instruction to Data collector	Built in software logics
**Household Socio Economic Scale**					
CRF1B	1	Record the main material used in the construction of floor	1|Mud/clay/earth<br>2|Sand<br>3|Dung<br>4|Raw wood planks<br>5|Palm/bamboo<br>6|Brick<br>7|Stone<br>8|Parquet or polished wood<br>9|Vinyl or asphalt<br>10|Ceramic tiles<br>11|Cement<br>12|Carpet<br>13|Polished stone/ marble/ Granite	This is an observation not a question since you will usually be able to see for yourself what kind of floor the house has. However, ask if you are not sure. If there is more than one kind of flooring material,<br>record the main type of material (the material that covers the largest amount of floor space)	None

CRF1B	2	Record the main material used in the construction of roof	1|No roof<br>2|Thatch/ palm leaf/ Reed/ grass<br>3|Mud<br>4|Sod/mud and grass mixture<br>5|Plastic/polythene sheeting<br>6|Rustic mat<br>7|Palm/bamboo<br>8|Raw wood planks/timber<br>9|Unburnt brick<br>10|Loosely packed stone<br>11|Metal/GI<br>12|Wood<br>13|Calamine/cement fiber<br>14|Asbestos sheets<br>15|RCC/RBC/cement/concrete<br>16|Roofing shingles<br>17|Tiles<br>18|Slate	This is an observation not a question since you will usually be able to see for yourself what kind of floor the house has. However, ask if you are not sure. If there is more than one kind of flooring material, record the main type of material (the material that covers the largest amount of floor space)	None
CRF1B	3	Record the main material used in the construction of exterior walls	1|No Walls<br>2|Cane/Palm/Trunks/Bamboo<br>3|Mud<br>4|Grass/Reeds/Thatch<br>5|Bamboo with Mud<br>6|Stone with Mud<br>7|Plywood<br>8|Cardboard<br>9|Unburnt Brick<br>10|Raw Wood/Reused Wood<br>11|Cement/Concrete<br>12|Stone with Lime/Cement<br>13|Burnt Bricks<br>14|Cement Blocks<br>15|Wood Planks/Shingles<br>16|GI/Metal/Asbestos Sheets	This is an observation not a question since you will usually be able to see for yourself what kind of floor the house has. However, ask if you are not sure. If there is more than one kind of flooring material, record the main type of material (the material that covers the largest amount of floor space)	None

CRF1B	4	What is the main source of drinking water for members of your household?	1|Piped to dwelling<br>2|Piped to yard / Plot<br>3|Piped to neighbor<br>4|Public Taps / Standpipe<br>5|Tube Well or Borehole<br>6|Protected Well<br>7|Unprotected Well<br>8|Protected Spring<br>9|Unprotected Spring<br>10|Rain Water<br>11|Tanker Truck<br>12|Cart with small tank<br>13|Surface Water (River / Dam/ Lake / Pond / Stream / Canal / Irrigation channel)<br>14|Bottled Water<br>15|Community RO Plant	**1|Piped to dwelling**: Pipe connected with in-house plumbing to one or more taps, e.g., in the kitchen and bathroom. Sometimes called a house connection.<br>**2|Piped to yard / Plot**: Pipe connected to a tap outside the house in the yard or plot. Sometimes called a yard connection.<br>**4|Public Taps / Standpipe**: Public water point from which community members may collect water. A standpipe may also be known as a public fountain or public tap. Public standpipes can have one or more taps and are typically made of brickwork, masonry or concrete.<br>**5|Tube Well or Borehole:** A deep hole that has been driven, bored or drilled with the purpose of reaching ground water supplies. Water is delivered from a tube well or borehole through a pump which may be human, animal, wind, electric, diesel or solar powered.<br>**6|Protected Well**: A dug well that is<br>(1) protected from runoff water through a well lining or casing that is raised above ground level and a platform that diverts spilled water away from the well and<br>(2) covered so that bird droppings and animals cannot fall down the hole. Both conditions must be observed for a dug well to be considered as protected.<br>**7|Unprotected Well:** A dug well which is<br>(1) unprotected from runoff water;<br>(2) unprotected from bird droppings and animals; or<br>(3) both.	None

				**8|Protected Spring**: A spring protected from runoff, bird droppings, and animals by a "spring box" which is typically constructed of brick, masonry, or concrete and is built around the spring so that water flows directly out of the box into a pipe without being exposed to outside pollution.<br>**9|Unprotected Spring**: A spring that is subject to runoff and/or bird droppings or animals. Unprotected springs typically do not have a "spring box".<br>**10|Rain Water:** Rain that is collected or harvested from surfaces by roof or ground catchment and stored in a container, tank or cistern.<br>**11|Tanker Truck:** Water is obtained from a provider who uses a truck to transport water into the community. Typically, the provider sells the water to households<br>**12|Cart with small tank:** Water is obtained from a provider who transports water into a community using a cart and then sells the water. The means for pulling the cart may be motorized or non-motorized (e.g., a bullock).<br>**13|Surface Water (River / Dam/ Lake / Pond / Stream / Canal / Irrigation channel):** Water located above ground and includes rivers, dams, lakes, ponds, streams, canals, and irrigation channels.<br>**14|Bottled Water:** Water that is bottled and sold to the household in bottles<br>**15|Community RO Plant:** Reverse Osmosis (RO) is a process for creating safe drinking water by forcing water under high pressure through a filter.	

CRF1B	5	What type of fuel does your household mainly use for cooking?	1|Electricity<br>2|LPG / Natural Gas 3|Bio Gas<br>4|Kerosene<br>5|Coal / Lignite<br>6|Charcoal 7|Wood<br>8|Straws / Shrubs / Grass<br>9|Agriculture Crop Waste<br>10|Dung Cakes<br>11|No food Cooked in Household	The use of some cooking fuels can also have adverse health consequences.<br>Remember that this question asks about fuel for cooking, not fuel for heating or lighting.<br>The category 'biogas' includes gases produced by fermenting manure in an enclosed pit.	None
CRF1B	6	What kind of toilet facility do the members of your household usually use?	1|Flush to Piped Sewer System<br>2|Flush to Septic Tank<br>3|Flush to a Pit Latrine<br>4|Flush to somewhere else<br>5|Flush to don't know where<br>6|Ventilated Improved Single PIT (VIP) Bio Gas latrine<br>7|Single Pit Latrine with slab<br>8|Single Pit Latrine without Slab / Open Pit<br>9|No facility/uses open space or field	**1|Flush to Piped Sewer System:** A system of sewer pipes (also called sewerage) that is designed to collect human excreta (faeces and urine) and wastewater and remove them from the household environment.<br>Sewerage systems consist of facilities for collection, pumping, treating and disposing of human excreta and wastewater.<br>**2|Flush to Septic Tank:** An excreta collection device consisting of a water-tight settling tank normally located underground, away from the house or toilet.<br>**3|Flush to a Pit Latrine:** A system that flushes excreta to a hole in the ground.<br>**4| Flush to somewhere else**: A system in which the excreta is deposited in or nearby the household environment in a location other than a sewer, septic tank, or pit, e.g., excreta may be flushed to the street, yard/plot, drainage ditch or other location.	None

				**6|Ventilated Improved Single PIT (VIP) Bio Gas latrine**: A latrine ventilated by a pipe extending above the latrine roof. The open end of the vent pipe is covered with gauze mesh or fly-proof netting and the inside of the super structure is kept dark.<br>**7|Single Pit Latrine with slab:** A latrine with a squatting slab, platform or seat firmly supported on all sides which is raised above the surrounding ground level to prevent surface water from entering the pit and for ease of cleaning.<br>**8|Single Pit Latrine without Slab / Open Pit:** A latrine without a squatting slab, platform or seat. An open pit is a rudimentary hole in the ground where excreta is collected.	
CRF1B	7	Do you share the toilet facility with any other household	0|No<br>1|Yes	Asks about whether the toilet facilities are shared with other households. we want to find out how many households, including the respondent's household, use the same facility. The number of households that share toilet facilities is an important measure of the level of hygiene in the household.	None
CRF1B	8	Does any member of this household own the following			None
CRF1B	8.1	Bank account or a post office account?	0|No<br>1|Yes	Ask if any member in the household has an account with a bank, post office, credit association or other similar organization in which they can deposit and withdraw funds. Record the appropriate answer	None

CRF1B	8.2	Health scheme or health insurance?	0|No<br>1|Yes	we want to know if any usual household member (excluding visitors who are not usual residents) has health insurance coverage of any kind, whether it be through the government (central or state health schemes), through other employers, or individually bought.	None
CRF1B	8.3	Own this house or any other house?	0|No<br>1|Yes	This question is asked to know if any household member owns any house irrespective of whether they are currently living in it or not.	None
CRF1B	8.4	Owns any agricultural land	0|No<br>1|Yes	Asks to find out whether any member of the household owns any land that can be used for agriculture. The land does not have to be near where the household lives. . 'Agricultural land' here includes only land which is being used or can be used for agricultural purposes.<br>Land used for grazing, brick kilns, etc. and other waste or barren land that cannot be cultivated should not be considered as 'agricultural land'.	None
CRF1B	9	How many rooms in this household are used for sleeping?	NA	Simply ask about the number of rooms that the household uses for sleeping. Include all the rooms where persons in the household sleep, even if those rooms are used for other purposes in the daytime (that is, they are not exclusively used for sleeping). If the household members usually sleep outside the house, you would record '00' for the number of rooms used for sleeping.	None

CRF1B	10	Number of members in the household	NA	A household may be one person or a group of persons who usually live and eat together. This is not the same as a family. A family includes only people who are related, but a household includes any people who live together, whether or not they are related.	None
CRF1B	11	Number of children below 5 years of age in the household	NA	Number of children less than 5 year of age.	Existing logic in software Validation _Number of children below 5 years of age should be less than number of members in Household
CRF1B	12	Number of domestic staff listed in household	NA		None
CRF1B	13	Does the household have the following items listed		The answers to these questions on ownership of certain items will be used as a rough measure of the socioeconomic status of the household. Read out each item and record the answer given after each item.<br>Do not leave any item(s) blank.	None
CRF1B	13.1	Electricity	0|No<br>1|Yes		None
CRF1B	13.2	Mattress	0|No<br>1|Yes		None
CRF1B	13.3	Pressure Cooker	0|No<br>1|Yes		None

CRF1B	13.5	Cot / Bed	0|No<br>1|Yes		None
CRF1B	13.6	Table	0|No<br>1|Yes		None
CRF1B	13.7	Eclectic Fan	0|No<br>1|Yes		None
CRF1B	13.8	Radio / Transistor	0|No<br>1|Yes		None
CRF1B	13.9	B / W Television	0|No<br>1|Yes		None
CRF1B	14	Colour television	0|No<br>1|Yes		None
CRF1B	14.1	Sewing Machine	0|No<br>1|Yes		None
CRF1B	14.2	Mobile Telephone	0|No<br>1|Yes		None
CRF1B	14.3	Landline Telephone	0|No<br>1|Yes		None
CRF1B	14.4	Internet	0|No<br>1|Yes		None
CRF1B	14.5	Computer	0|No<br>1|Yes		None
CRF1B	14.6	Refrigerator	0|No<br>1|Yes		None
CRF1B	14.7	AC / Cooler	0|No<br>1|Yes		None
CRF1B	14.8	Washing Machine	0|No<br>1|Yes		None

CRF1B	14.9	Watch or Clock	0|No<br>1|Yes		None
CRF1B	15	Water Pump	0|No<br>1|Yes		None
CRF1B	15.1	Chair	0|No<br>1|Yes		None
CRF1B	15.2	Thresher	0|No<br>1|Yes		None
CRF1B	16	Vehicle own by household	0|No<br>1|Yes	The answers to these questions on ownership of VEHICLES will be used as a rough measure of the socioeconomic status of the household. Read out each item and record the answer given after each item.<br>Do not leave any item(s) blank.	None
CRF1B	16.1	Bi Cycle	0|No<br>1|Yes		None
CRF1B	16.2	Motorcycle / Scooter	0|No<br>1|Yes		None
CRF1B	16.3	Animal Drawn Cart	0|No<br>1|Yes		None
CRF1B	16.4	Car / 4-Wheeler	0|No<br>1|Yes		None
CRF1B	16.5	Tractor	0|No<br>1|Yes		None

Form	Q No	Questions	Response options	Instruction to Data collector	Built in software logics
**AFI/ILI surveillance form or Follow-up call**					
CRF3B	*1*	Household ID			None
CRF3B	*2*	Individual ID			None
CRF3B	*3*	Name			None
CRF3B	*4*	Present Condition of the participant on the day of contact	1|Well<br>2|Sick at home<br>3|Continued hospitalisation from last contact<br>4|Fresh hospitalisation<br>5|Expired	Ask the participant for the present health condition	Existing Logic:<br>If response is "1", go to 5<br>If response is "2" go to 5<br>If response is "5", form should end<br>If response is "4", trigger hospitalization form in MO tab<br>If option"3" will be visible from Second call,
CRF3B	*4.1*	If fresh hospitalisation, is the respondent able to answer regarding condition of participant	0|No<br>1|Yes		Existing Logic:<br>If response is "No", end the form
CRF3B	*4.2*	If fresh hospitalisation, is diagnosis known to respondent?	0|No<br>1|Yes		Existing Logic:<br>If response is "No" skip 4.3
CRF3B	*4.3*	If "Yes", specify diagnosis			
CRF3B	***5***	**Fever**			
CRF3B	*5.1*	Was this symptom present during the last call?	0|No<br>1|Yes	Ask the participant whether fever was present during the last contact	This question will be hidden for all symptoms in first call<br>Auto-filled from second call onwards based on answers of previous call
CRF3B	5.2	Does participant have this symptom today?	0|No<br>1|Yes	Ask the participant if he/she have fever today	Existing Logic:<br>If response "Yes", go to 5.3<br>If response is "No", Skip 5.3 and 5.4

CRF3B	5.3	Since how many days have you had this symptom		Enquire the days the participant had this symptom	Existing Logic:<br>Range 1 to 21
CRF3B	5.4	Date of onset			Existing Logic:<br>Cannot not be a future date
CRF3B	5.5	Did the participant have this symptom since the last time we contacted you?	0|No<br>1|Yes		Existing Logic:<br>If response is "Yes", go to 5.6<br>If response is "No", skip 5.6
CRF3B	5.6	For how many days did the participant have this symptom?			Existing Logic:<br>Range 5 to 21
CRF3B	**6**	**Cough**			
CRF3B	*6.1*	Was this symptom present during the last call?	0|No<br>1|Yes		This question will be hidden for all symptoms in first call<br>Auto-filled from second call onwards based on answers of previous call
CRF3B	6.2	Does participant have this symptom today?	0|No<br>1|Yes		If response "Yes", go to 6.3<br>If response is "No", Skip 6.3 and 6.4
CRF3B	6.3	Since how many days have you had this symptom			Existing Logic:<br>Range 1 to 21
CRF3B	6.4	Date of onset			Existing Logic:<br>Cannot not be a future date
CRF3B	6.5	Did the participant have this symptom since the last time we contacted you?	0|No<br>1|Yes		If response is "Yes", go to 6.6<br>If response is No, skip 6.6.
CRF3B	6.6	For how many days did the participant have this symptom?			Existing Logic:<br>Range 5 to 21
CRF3B	***7***	**Difficulty in breathing:**			
CRF3B	*7.1*	Was this symptom present during the last call?	0|No<br>1|Yes		This question will be hidden for all symptoms in first call<br>Auto-filled from second call onwards based on answers of previous call

CRF3B	7.2	Does participant have this symptom today?	0|No<br>1|Yes		If response "Yes", go to 7.3<br>If response is "No", Skip 7.3 and 7.4
CRF3B	7.3	Since how many days have you had this symptom			Existing Logic:<br>Range 1 to 21
CRF3B	7.4	Date of onset			Existing Logic:<br>Cannot not be a future date
CRF3B	7.5	Did the participant have this symptom since the last time we contacted you?	0|No<br>1|Yes		
CRF3B	7.6	For how many days did the participant have this symptom?			Existing Logic:<br>Range 5 to 21
CRF3B	***8***	**Nasal congestion**			
CRF3B	*8.1*	Was this symptom present during the last call?	0|No<br>1|Yes		This question will be hidden for all symptoms in first call<br>Auto-filled from second call onwards based on answers of previous call
CRF3B	8.2	Does participant have this symptom today?	0|No<br>1|Yes		If response "Yes", go to 8.3<br>If response is "No", Skip 8.3 and 8.4
CRF3B	8.3	Since how many days have you had this symptom			Existing Logic:<br>Range 1 to 21
CRF3B	8.4	Date of onset			Existing Logic:<br>Cannot not be a future date
CRF3B	8.5	Did the participant have this symptom since the last time we contacted you?	0|No<br>1|Yes		If response is "Yes", go to 8.6<br>If response is "No", skip 8.6
CRF3B	8.6	For how many days did the participant have this symptom?			Existing Logic:<br>Range 5 to 21

CRF3B	**9**	**Sore throat:**			
CRF3B	*9.1*	Was this symptom present during the last call?	0|No<br>1|Yes		This question will be hidden for all symptoms in first call<br>Auto-filled from second call onwards based on answers of previous call
CRF3B	9.2	Does participant have this symptom today?	0|No<br>1|Yes		If response "Yes", go to 9.3<br>If response is “No”, Skip 9.3 and 9.4
CRF3B	9.3	Since how many days have you had this symptom			Existing Logic:<br>Range 1 to 21
CRF3B	9.4	Date of onset			Existing Logic:<br>Cannot not be a future date
CRF3B	9.5	Did the participant have this symptom since the last time we contacted you?	0|No<br>1|Yes		If response is "Yes", go to 9.6<br>If response is “No”, skip 9.6
CRF3B	9.6	For how many days did the participant have this symptom?			Existing Logic:<br>Range 5 to 21
CRF3B	**10**	**Blood in sputum:**			
CRF3B	*10.1*	Was this symptom present during the last call?	0|No<br>1|Yes		This question will be hidden for all symptoms in first call<br>Auto-filled from second call onwards based on answers of previous call
CRF3B	10.2	Does participant have this symptom today?	0|No<br>1|Yes		If response "Yes", go to 10.3<br>If response is “No”, Skip 10.3 and 10.4
CRF3B	10.3	Since how many days have you had this symptom			Existing Logic:<br>Range 1 to 21
CRF3B	10.4	Date of onset			Existing Logic:<br>Cannot not be a future date
CRF3B	10.5	Did the participant have this symptom since the last time we contacted you?	0|No<br>1|Yes		If response is "Yes", go to 10.6<br>If response is “No”, skip 10.6
CRF3B	10.6	For how many days did the participant have this symptom?			Existing Logic:<br>Range 5 to 21

CRF3B	***11***	**Blue spots/bleeding spots on the skin:**			
CRF3B	*11.1*	Was this symptom present during the last call?	0|No<br>1|Yes		This question will be hidden for all symptoms in first call<br>Auto-filled from second call onwards based on answers of previous call
CRF3B	11.2	Does participant have this symptom today?	0|No<br>1|Yes		If response "Yes", go to 11.3<br>If response is “No”, Skip 11.3 and 11.4
CRF3B	11.3	Since how many days have you had this symptom			Existing Logic:<br>Range 1 to 21
CRF3B	11.4	Date of onset			Existing Logic:<br>Cannot not be a future date
CRF3B	11.5	Did the participant have this symptom since the last time we contacted you?	0|No<br>1|Yes		If response is “Yes”, go to 11.6<br>If response is “No”, skip 11.6
CRF3B	11.6	For how many days did the participant have this symptom?			Existing Logic:<br>Range 5 to 21
CRF3B	***12***	**Blood in vomitus:**			
CRF3B	*12.1*	Was this symptom present during the last call?	0|No<br>1|Yes		This question will be hidden for all symptoms in first call<br>Auto-filled from second call onwards based on answers of previous call
CRF3B	12.2	Does participant have this symptom today?	0|No<br>1|Yes		If response "Yes", go to 12.3<br>If response is “No”, Skip 12.3 and 12.4
CRF3B	12.3	Since how many days have you had this symptom			Existing Logic:<br>Range 1 to 21
CRF3B	12.4	Date of onset			Existing Logic:<br>Cannot not be a future date

CRF3B	12.5	Did the participant have this symptom since the last time we contacted you?	0|No<br>1|Yes		If response is “Yes”, go to 12.6<br>If response is “No”, skip 12.6
CRF3B	12.6	For how many days did the participant have this symptom?			Existing Logic:<br>Range 5 to 21
CRF3B	***13***	**Bleeding from the nose:**			
CRF3B	*13.1*	Was this symptom present during the last call?	0|No<br>1|Yes		This question will be hidden for all symptoms in first call<br>Auto-filled from second call onwards based on answers of previous call
CRF3B	13.2	Does participant have this symptom today?	0|No<br>1|Yes		If response "Yes", go to 13.3<br>If response is “No”, Skip 13.3 and 13.4
CRF3B	*13.3*	Since how many days have you had this symptom			Existing Logic:<br>Range 1 to 21
CRF3B	13.4	Date of onset			Existing Logic:<br>Cannot not be a future date
CRF3B	*13.5*	Did the participant have this symptom since the last time we contacted you?	0|No<br>1|Yes		If response is “Yes”, go to 13.6<br>If response is “No”, skip 13.6
CRF3B	13.6	For how many days did the participant have this symptom?			Existing Logic:<br>Range 5 to 21
CRF3B	***14***	**Joint Pain:**			
CRF3B	*14.1*	Was this symptom present during the last call?	0|No<br>1|Yes		This question will be hidden for all symptoms in first call<br>Auto-filled from second call onwards based on answers of previous call
CRF3B	14.2	Does participant have this symptom today?	0|No<br>1|Yes		If response "Yes", go to 14.3<br>If response is “No”, Skip 14.3 and 14.4
CRF3B	*14.3*	Since how many days have you had this symptom			Existing Logic:<br>Range 1 to 21

CRF3B	14.4	Date of onset			Existing Logic:<br>Cannot not be a future date
CRF3B	*14.5*	Did the participant have this symptom since the last time we contacted you?	0|No<br>1|Yes		If response is “Yes”, go to 14.6<br>If response is “No”, skip 14.6
CRF3B	14.6	For how many days did the participant have this symptom?			Existing Logic:<br>Range 5 to 21
CRF3B	***15***	**Body ache:**			
CRF3B	*15.1*	Was this symptom present during the last call?	0|No<br>1|Yes		This question will be hidden for all symptoms in first call<br>Auto-filled from second call onwards based on answers of previous call
CRF3B	15.2	Does participant have this symptom today?	0|No<br>1|Yes		If response "Yes", go to 15.3<br>If response is “No”, Skip 15.3 and 15.4
CRF3B	*15.3*	Since how many days have you had this symptom			Existing Logic:<br>Range 1 to 21
CRF3B	15.4	Date of onset			Existing Logic:<br>Cannot not be a future date
CRF3B	*15.5*	Did the participant have this symptom since the last time we contacted you?	0|No<br>1|Yes		If response is “Yes”, go to 15.6<br>If response is “No”, skip 15.6
CRF3B	15.6	For how many days did the participant have this symptom?			Existing Logic:<br>Range 5 to 21
CRF3B	***16***	**Headache/pain behind eye ball:**			
CRF3B	*16.1*	Was this symptom present during the last call?	0|No<br>1|Yes		This question will be hidden for all symptoms in first call<br>Auto-filled from second call onwards based on answer of previous call
CRF3B	16.2	Does participant have this symptom today?	0|No<br>1|Yes		If response "Yes", go to 16.3<br>If response is “No”, Skip 16.3 and 16.4

CRF3B	16.3	Since how many days have you had this symptom			Existing Logic:<br>Range 1 to 21
CRF3B	16.4	Date of onset			Existing Logic:<br>Cannot not be a future date
CRF3B	16.5	Did the participant have this symptom since the last time we contacted you?	0|No<br>1|Yes		If response is “Yes”, go to 16.6<br>If response is “No”, skip 16.6
CRF3B	16.6	For how many days did the participant have this symptom?			Existing Logic:<br>Range 5 to 21
CRF3B	***17***	**Loss of appetite;**			
CRF3B	*17.1*	Was this symptom present during the last call?	0|No<br>1|Yes		This question will be hidden for all symptoms in first call<br>Auto-filled from second call onwards based on answers of previous call
CRF3B	17.2	Does participant have this symptom today?	0|No<br>1|Yes		If response "Yes", go to 17.3<br>If response is “No”, Skip 17.3 and 17.4
CRF3B	17.3	Since how many days have you had this symptom			Existing Logic:<br>Range 1 to 21
CRF3B	17.4	Date of onset			Existing Logic:<br>Cannot not be a future date
CRF3B	17.5	Did the participant have this symptom since the last time we contacted you?	0|No<br>1|Yes		If response is "Yes", go to 17,6<br>If response is “No”, skip 17.6
CRF3B	17.6	For how many days did the participant have this symptom?			Existing Logic:<br>Range 5 to 21
CRF3B	***18***	**Nausea/vomiting:**			
CRF3B	*18.1*	Was this symptom present during the last call?	0|No<br>1|Yes		This question will be hidden for all symptoms in first call<br>Auto-filled from second call onwards based on answers of previous call

CRF3B	18.2	Does participant have this symptom today?	0|No<br>1|Yes		If response "Yes", go to 18.3<br>If response is "No", Skip 18.3 and 18.4
CRF3B	18.3	Since how many days have you had this symptom			Existing Logic:<br>Range 1 to 21
CRF3B	18.4	Date of onset			Existing Logic:<br>Cannot be future date
CRF3B	18.5	Did the participant have this symptom since the last time we contacted you?	0|No<br>1|Yes		If response is "Yes", go to 18.6<br>If response is "No", skip 18.6
CRF3B	18.6	For how many days did the participant have this symptom?			Existing Logic:<br>Range 5 to 21
CRF3B	***19***	**Diarrhea:**			
CRF3B	*19.1*	Was this symptom present during the last call?	0|No<br>1|Yes		This question will be hidden for all symptoms in first call<br>Auto-filled from second call onwards based on answers of previous call
CRF3B	19.2	Does participant have this symptom today?	0|No<br>1|Yes		If response "Yes", go to 19.3<br>If response is "No", Skip 19.3 and 19.4
CRF3B	19.3	Since how many days have you had this symptom			Existing Logic:<br>Range 1 to 21
CRF3B	19.4	Date of onset			Existing Logic:<br>Cannot be a future date
CRF3B	19.5	Did the participant have this symptom since the last time we contacted you?	0|No<br>1|Yes		If response is "Yes", go to 19.6<br>If response is "No", skip 19.6
CRF3B	19.6	For how many days did the participant have this symptom?			Existing Logic:<br>Range 5 to 21

CRF3B	***20***	**Abdominal pain:**			
CRF3B	*20.1*	Was this symptom present during the last call?	0|No<br>1|Yes		This question will be hidden for all symptoms in first call<br>Auto-filled from second call onwards based on answers of previous call
CRF3B	20.2	Does participant have this symptom today?	0|No<br>1|Yes		If response "Yes", go to 20.3<br>If response is “No”, Skip 20.3 and 20.4
CRF3B	20.3	Since how many days have you had this symptom			Existing Logic:<br>Range 1 to 21
CRF3B	20.4	Date of onset			Existing Logic:<br>Date cannot be a future date
CRF3B	20.5	Did the participant have this symptom since the last time we contacted you?	0|No<br>1|Yes		If response is "Yes", go to 20.6<br>If response is "No", skip 20.6
CRF3B	20.6	For how many days did the participant have this symptom?			Existing Logic:<br>Range 5 to 21
CRF3B	***21***	**Loss of taste:**			
CRF3B	*21.1*	Was this symptom present during the last call?	0|No<br>1|Yes		This question will be hidden for all symptoms in first call<br>Auto-filled from second call onwards based on answers of previous call
CRF3B	21.2	Does participant have this symptom today?	0|No<br>1|Yes		If response "Yes", go to 21.3<br>If response is “No”, Skip 21.3 and 21.4
CRF3B	21.3	Since how many days have you had this symptom			Existing Logic:<br>Range 1 to 21
CRF3B	21.4	Date of onset			Existing Logic:<br>Date cannot be a future date

CRF3B	21.5	Did the participant have this symptom since the last time we contacted you?	0|No<br>1|Yes		If response is "Yes", go to 21.6<br>If response is "no", skip 21.6
CRF3B	21.6	For how many days did the participant have this symptom?			Existing Logic:<br>Range 5 to 21
CRF3B	***22***	**Loss of smell:**			
CRF3B	*22.1*	Was this symptom present during the last call?	0|No<br>1|Yes		This question will be hidden for all symptoms in first call<br>Auto-filled from second call onwards based on answers of previous call
CRF3B	22.2	Does participant have this symptom today?	0|No<br>1|Yes		If response "Yes", go to 22.3<br>If response is "No", Skip 22.3 and 22.4
CRF3B	22.3	Since how many days have you had this symptom			Existing Logic:<br>Range 1 to 21
CRF3B	22.4	Date of onset			Existing Logic:<br>Date cannot be a future date
CRF3B	22.5	Did the participant have this symptom since the last time we contacted you?	0|No<br>1|Yes		If response is "Yes", go to 22.6<br>If response is "No", skip 22.6
CRF3B	22.6	For how many days did the participant have this symptom?			Existing Logic:<br>Range 5 to 21
CRF3B	***23***	**Refusal to feed (in children below 5 years of age)**			
CRF3B	*23.1*	Was this symptom present during the last call?	0|No<br>1|Yes		This question will be hidden for all symptoms in first call<br>Auto-filled from second call onwards based on answers of previous call
CRF3B	23.2	Does participant have this symptom today?	0|No<br>1|Yes		If response "Yes", go to 23.3<br>If response is "No", Skip 23.3 and 23.4
CRF3B	23.3	Since how many days have you had this symptom			Existing Logic:<br>Range 1 to 21

CRF3B	23.4	Date of onset			Existing Logic:<br>Date cannot be a future date
CRF3B	23.5	Did the participant have this symptom since the last time we contacted you?	0|No<br>1|Yes		If response is “Yes”, go to 23.6<br>If response is “No”, skip 23.6
CRF3B	23.6	For how many days did the participant have this symptom?			Existing Logic:<br>Range 5 to 21
CRF3B	***24***	**COVID test**			
CRF3B	*24.1*	Did you (the participant) get any COVID test done since the last time we contacted you?	0|No<br>1|Yes		If response is “No”, go to 25
CRF3B	*24.2*	Date of test	Select date		Existing Logic:<br>Date cannot be a future date
CRF3B	*24.3*	Result of test	1|Positive<br>2|Negative		
CRF3B	*24.4*	Source of information	1|As reported<br>2|Lab report		
CRF3B	***25***	***Social History (In relation to COVID)***	NA		
CRF3B	*25.1*	*History of any contact with a person diagnosed with COVID in past 14 days?*	0|No<br>1|Yes<br>2|Don't Know		
CRF3B	*25.2*	*History of attending or organizing any social gathering in past 14 days? (Religious gathering, Community meetings, Wedding, Funeral etc. or any gathering of people other than members of the household who came together to celebrate or conduct a function)*	1|Yes (within household)<br>2|Yes (outside the household)<br>3|No		If response is “No”, go to 26

CRF3B	*25.3*	If "Yes", type of Gathering	1|Religious Gathering<br>2|Wedding<br>3|Funeral<br>4|Other family gatherings<br>5|Community Gatherings<br>6|Academic Gatherings<br>7|Sports Gatherings		
CRF3B	*25.4*	*Any history of COVID positive case reported from that gathering*	0|No<br>1|Yes<br>2|Don't Know		
CRF3B	***26***	***Travel History***			
CRF3B	*26.1*	*Do you have Travel History since last contact*	0|No<br>1|Yes		If response is "No", go to 26.4
CRF3B	*26.2*	*If "Yes", type of travel*	1|Within the district<br>2|Outside district but within the state<br>3|Outside state but within the country<br>4|International		
CRF3B	*26.3*	*If "Yes", what was the duration of the visit (travel and stay) in days*	NA		
CRF3B	*26.4*	*Did anyone who had travelled outside the district visit the household since last contact?*	0|No<br>1|Yes		If Response is "No", skip 26.5
CRF3B	*26.5*	*If "Yes", where did the visitor arrive from?*	1|Outside district but within the state<br>2|Outside state but within the country<br>3|International		

Form	Q No	Questions	Response options	Instruction to Data collector	Built in software logics
**Treatment History**<br>This form is to be filled for all participants who had reported fever during the telephonic AFI/ILI surveillance and recovered from the episode without requiring hospitalization					
CRF4A	1	Did you seek medical advice for symptoms?	1|Yes, from practitioner/health care facility/health care worker<br>2|No, managed at home without any remedies<br>3|No, managed at home with self medications or over the counter drugs<br>4|Don't know	Ask the participant whether he/she had medical advice for symptoms	If Response is 1, skip 1.1<br>If Response is 2 go to 12 and end form<br>If response is 3, go 1.1 and end form<br>If Response is 4 go to 12
CRF4A	1.1	What was the type of medication that you took?	1|Allopathic drugs<br>2|AYUSH drugs (Ayurvedic, Unani, Siddha medications)<br>3|Home remedies/ Traditional remedies<br>4|Don't know	Ask the participant for the Type of medication he had for the symptom	None
CRF4A	2	How many facilities/practitioners did you approach to seek treatment?	1|1<br>2|2<br>3|3	Ask the participant that how many practitioners they approach for medical advice, Maximum 3	If response is “1”, skip 6 to 11.39<br>If response is “2”, skip 9 to 11.39

CRF4A	3	For 1st Health Facility: Type of health facility/ practitioner accessed for treatment	1|Public facility<br>2|Private facility<br>3|Known physician (Family, social acquaintances, friends)<br>4|Health care worker (e.g.: Home visit by ASHAs, ANMs)<br>5|Traditional healers/Unqualified practitioners	Ask the participant for the kind of health facility he accessed for treatment, record the response	If response is “1”, skip 3.2<br>If response is “2”, skip 3.1<br>if response is “3 or 4 or 5”, skip 3.1 and 3.2
CRF4A	*3.1*	For 1st Health Facility: If Public facility, mention type	1|HWC / Sub Center<br>2|PHC / CHC<br>3|District Hospital<br>4|Medical College		None
CRF4A	*3.2*	For 1st Health Facility: If Private facility, mention type	1|Private Clinics<br>2|Private Nursing Home<br>3|NGO/Trust Hospital<br>4|Private hospital		None
CRF4A	*3.3*	For 1st Health Facility: Mention name and address of facility	NA		None
CRF4A	*4*	For 1st Health Facility: What type of treatment did you receive?	1| No treatment was advised<br>2|Treated as out-patient<br>3|Treated on day care basis (kept under observation for <24 hours and discharged)<br>4|Admitted for treatment (IP)	Ask the participant for the type of treatment they received, and record their response	If response is “1”, skip 4.1 to 4.6<br>IF response is “2 or 3”, skip 4.1 to 4.5
CRF4A	*4.1*	For 1st Health Facility: For those who were admitted and treated on an in-patient basis, record date of admission	NA	Ask the participant to show if any record of date of admission	Existing Logic:<br>Future date will not be allowed

CRF4A	*4.2*	For 1st Health Facility: For those who were admitted and treated on an in-patient basis, date of discharge	NA	Ask the participant to show if any record of date of discharge	Existing Logic:<br>Future date will not be allowed
CRF4A	*4.3*	For 1st Health Facility: For those who were admitted and treated on an in-patient basis, was the participant admitted to the ICU?	0|No<br>1|Yes	Ask the participant if they were admitted to the ICU during their admission at hospital	None
CRF4A	*4.4*	For 1st Health Facility: For those who were admitted and treated on an in-patient basis, was the participant put on ventilatory support?	0|No<br>1|Yes	Ask the participant if they were given any ventilator support during their admission at hospital	None
CRF4A	*4.5*	For 1st Health Facility: For those who were admitted and treated on an in-patient basis, did the participant require oxygen therapy?	0|No<br>1|Yes	Ask the participant if they were given any oxygen support during their admission at hospital	None
CRF4A	*4.6*	For 1st Health Facility: What was the type of medication that you were prescribed?	1|Allopathic drugs<br>2|AYUSH drugs (Ayurvedic, Unani, Siddha medications)<br>3|Home remedies/ Traditional remedies<br>4|Don't know	Ask the participant for the type of medication they received, and record their response	None
CRF4A	*5*	For 1st Health Facility: Was the participant diagnosed to have any of the acute febrile illnesses of interest for this study?	0|No<br>1|Yes	Ask the participant if they were diagnosed with any AFI like COVID 19, Dengue, Chikungunya, Typhoid, Malaria, Japanese Encephalitis, Scrub Typhus, [illegible]	If response is “No”, go to 5.37

CRF4A	*5.1*	Was a diagnosis of COVID-19 made? (For Health Facility 1)	0|Not Diagnosed<br>1|Only Clinical Diagnosis<br>2|Clinical and Laboratory Diagnosis<br>3|No details available	Ask the participant if they had any diagnosis for covid 19, record the response	If response is “0 or 1 or 3”, go to 5.8<br>If response is “2”, go to 5.2
CRF4A	*5.2*	For Covid-19 RTPCR: Result (For Health Facility 1)	0|Not performed<br>1|Positive 2|Negative<br>3|Inconclusive	Ask the participant whether he/she had RTPCR test, Record the response	If response is “No”, skip 5.3
CRF4A	*5.3*	COVID-19 RTPCR 1: Date of Testing (For Health Facility 1)	NA	Click unknow if the date is unavailable	Existing Logic:<br>Date cannot be future
CRF4A	*5.4*	Covid-19 Rapid Antigen Test: Result? (For Health Facility 1)	0|Not performed<br>1|Positive<br>2|Negative<br>3|Inconclusive	Ask the participant whether he/she had Rapid antigen test, Record the response	If response is “No”, skip 5.5
CRF4A	*5.5*	Covid-19 Rapid Antigen Test: Date of Testing (For Health Facility 1)	NA	Click unknow if the date is unavailable	Existing Logic:<br>Date cannot be future
CRF4A	*5.6*	Covid-19 ELISA: Result? (For Health Facility 1)	0|Not performed<br>1|Positive<br>2|Negative<br>3|Inconclusive	Ask the participant whether he/she had Elisa test, Record the response	If response is “No”, skip 5.7
CRF4A	*5.7*	Covid-19 ELISA: Date of Testing (For Health Facility 1)	NA	Click unknow if the date is unavailable	Existing Logic:<br>Date cannot be future
CRF4A	*5.8*	Was the diagnosis of dengue made? (For Health Facility 1)	0|Not diagnosed<br>1|Only Clinical Diagnosis<br>2|Clinical and Laboratory Diagnosis<br>3|No details available	Ask the participant if they had any diagnosis for Dengue, record the response	If response is “0 or 1 or3”, go to 5.21<br>If response is “2”, go to 5.9

CRF4A	*5.9*	Dengue Rapid Test for NS1 Ag/IgM: Result? (For Health Facility 1)	0|Not performed<br>1|Positive<br>2|Negative<br>3|Inconclusive	Ask the participant whether he/she Dengue Rapid Test for NS1 Ag/IgM, Record the response	If response is "No", skip 5.10
CRF4A	*5.10*	Dengue Rapid Test for NS1 Ag/IgM: Date of Testing (For Health Facility 1)	NA	Click unknow if the date is unavailable	Existing Logic:<br>Date cannot be future
CRF4A	*5.11*	Dengue Rapid test for IgG: Result (For Health Facility 1)	0|Not performed<br>1|Positive 2|Negative<br>3|Inconclusive	Ask the participant whether he/she Dengue Rapid test for IgG, Record the response	If response is "No", skip 5.12
CRF4A	*5.12*	Dengue Rapid test for IgG: Date of Testing (For Health Facility 1)	NA	Click unknow if the date is unavailable	Existing Logic:<br>Date cannot be future
CRF4A	*5.13*	Dengue ELISA for NS1: Result? (For Health Facility 1)	0|Not performed<br>1|Positive<br>2|Negative<br>3|Inconclusive	Ask the participant whether he/she had Dengue ELISA for NS1, Record the response	If response is "No", skip 5.14
CRF4A	*5.14*	Dengue ELISA for NS1: Date of Testing (For Health Facility 1)	NA	Click unknow if the date is unavailable	Existing Logic:<br>Date cannot be future
CRF4A	*5.15*	Dengue ELISA for IgM: Result? (For Health Facility 1)	0|Not performed<br>1|Positive<br>2|Negative<br>3|Inconclusive	Ask the participant whether he/she Dengue ELISA for IgM, Record the response	If response is "No", skip 5.16
CRF4A	*5.16*	For Dengue ELISA for IgM Date of Testing (For Health Facility 1)	NA	Click unknow if the date is unavailable	Existing Logic:<br>Date cannot be future
CRF4A	*5.17*	Dengue ELISA for IgG: Result? (For Health Facility 1)	0|Not performed<br>1|Positive<br>2|Negative<br>3|Inconclusive	Ask the participant whether he/she Dengue ELISA for IgG, Record the response	If response is "No", skip 5.18
CRF4A	*5.18*	Dengue ELISA for IgG: Date of Testing (For Health Facility 1)	NA	Click unknow if the date is unavailable	Existing Logic:<br>Date cannot be future

CRF4A	*5.19*	Dengue RTPCR: Result? (For Health Facility 1)	0|Not performed<br>1|Positive<br>2|Negative<br>3|Inconclusive	Ask the participant whether he/she Dengue RTPCR: Result? Record the response	If response is "No", skip 5.20
CRF4A	*5.20*	Dengue RTPCR: Date of Testing (For Health Facility 1)	NA	Click unknow if the date is unavailable	Existing Logic:<br>Date cannot be future
CRF4A	*5.21*	Was a diagnosis of chikungunya made? (For Health Facility 1)	0|Not diagnosed<br>1|Only Clinical Diagnosis<br>2|Clinical and Laboratory Diagnosis<br>3|No details available	Ask the participant if they had any diagnosis for chikungunya, record the response	If response is " 0 or 1 or 3", go to 5.32<br>If response is " 2", go to 5.22
CRF4A	*5.22*	Chikungunya Rapid Test for IgM: Result? (For Health Facility 1)	0|Not performed<br>1|Positive<br>2|Negative<br>3|Inconclusive	Ask the participant if they had any diagnosis for Chikungunya, record the response	If response is "No", skip 5.23
CRF4A	*5.23*	Chikungunya: Rapid Test IgM: Date of Testing (For Health Facility 1)	NA	Click unknow if the date is unavailable	Existing Logic:<br>Date cannot be future
CRF4A	*5.24*	Chikungunya Rapid test for IgG: Result? (For Health Facility 1)	0|Not performed<br>1|Positive<br>2|Negative<br>3|Inconclusive	Ask the participant whether he/she Chikungunya Rapid test for IgG, Record the response	If response is "No", skip 5.25
CRF4A	*5.25*	Chikungunya: Rapid test for IgG Date of Testing (For Health Facility 1)	NA	Click unknow if the date is unavailable	Existing Logic:<br>Date cannot be future
CRF4A	*5.26*	Chikungunya ELISA for IgM: Result? (For Health Facility 1)	0|Not performed<br>1|Positive 2|Negative<br>3|Inconclusive	Ask the participant whether he/she had Chikungunya ELISA for IgM, Record the response	If response is "No", skip 5.27
CRF4A	*5.27*	Chikungunya ELISA for IgM Date of Testing (For Health Facility 1)	NA	Click unknow if the date is unavailable	Existing Logic:<br>Date cannot be future

CRF4A	*5.28*	Chikungunya ELISA for IgG: Result (For Health Facility 1)	0|Not performed<br>1|Positive<br>2|Negative<br>3|Inconclusive	Ask the participant whether he/she had Chikungunya ELISA for IgG, Record the response	If response is "No", skip 5.29
CRF4A	*5.29*	Chikungunya ELISA for IgG: Date of Testing (For Health Facility 1)	NA	Click unknow if the date is unavailable	Existing Logic:<br>Should not be a future
CRF4A	*5.30*	For Chikungunya RTPCR. Result? (For Health Facility 1)	0|Not performed<br>1|Positive<br>2|Negative<br>3|Inconclusive	Ask the participant whether he/she had Chikungunya RTPCR, Record the response	If response is "No", skip 5.31
CRF4A	*5.31*	Chikungunya RTPCR Date of Testing (For Health Facility 1)	NA	Click unknow if the date is unavailable	Existing Logic:<br>Should not be a future
CRF4A	*5.32*	Was a diagnosis of Typhoid made? (For Health Facility 1)	0|Not diagnosed<br>1|Only Clinical Diagnosis<br>2|Clinical and Laboratory Diagnosis<br>3|No details available	Ask the participant if he/she had any diagnosis for typhoid	None
CRF4A	*5.33*	Was a diagnosis of Malaria made? (For Health Facility 1)	0|Not diagnosed<br>1|Only Clinical Diagnosis<br>2|Clinical and Laboratory Diagnosis<br>3|No details available	Ask the participant if he/she had any diagnosis for Malaria	None
CRF4A	*5.34*	Was a diagnosis of Japanese Encephalitis made? (For Health Facility 1)	0|Not diagnosed<br>1|Only Clinical Diagnosis<br>2|Clinical and Laboratory Diagnosis<br>3|No details available	Ask the participant if he/she had any diagnosis for JE	None

CRF4A	*5.35*	Was a diagnosis of Scrub Typhus made? (For Health Facility 1)	0|Not diagnosed<br>1|Only Clinical Diagnosis<br>2|Clinical and Laboratory Diagnosis<br>3|No details available	Ask the participant if he/she had any diagnosis for scrub typhus	None
CRF4A	*5.36*	Was a diagnosis of Leptospirosis made? (For Health Facility 1)	0|Not diagnosed<br>1|Only Clinical Diagnosis<br>2|Clinical and Laboratory Diagnosis<br>3|No details available	Ask the participant if he/she had any diagnosis for leptospirosis	None
CRF4A	*5.37*	Other's Specify for 1st Health Facility: Was the participant diagnosed to have any other AFI other than mentioned above	0|No<br>1|Yes	Ask the participant if he/she had diagnosed with any other illness and record the response	If response is "No", skip 5.38 and 5.39
CRF4A	*5.38*	If Yes, Mention the name of illness diagnosed			None
CRF4A	*5.39*	For Other illness mentioned, what was the basis of diagnosis (For Health Facility 1)	0|Not diagnosed<br>1|Only Clinical Diagnosis<br>2|Clinical and Laboratory Diagnosis<br>3|No details available		None
CRF4A	*6*	For 2nd Health Facility: Type of health facility/ practitioner accessed for treatment	1|Public facility<br>2|Private facility<br>3|Known physician (Family, social acquaintances, friends)<br>4|Health care worker (e.g., Home visit by ASHAs, ANMs)<br>5|Traditional healers/Unqualified practitioners	Ask the participant for the kind of health facility he accessed for treatment, record the response	If response is "1", skip 6.2<br>If response is "2", skip 6.1<br>if response is "3 or 4 or 5", skip 6.1 and 6.2

CRF4A	*6.1*	For 2ndHealth Facility: If Public facility, mention type	1|HWC / Sub Center<br>2|PHC / CHC<br>3|District Hospital<br>4|Medical College		None
CRF4A	*6.2*	For 2nd Health Facility: If Private facility, mention type	1|Private Clinics<br>2|Private Nursing Home<br>3|NGO/Trust Hospital<br>4|Private hospital		None
CRF4A	*6.3*	For 2nd Health Facility: Mention name and address of facility			None
CRF4A	*7*	For 2nd Health Facility: What type of treatment did you receive?	1| No treatment was advised<br>2|Treated as out-patient<br>3|Treated on day care basis (kept under observation for <24 hours and discharged)<br>4|Admitted for treatment (IP)	Ask the participant for the type of treatment they received, and record their response	If response is "1", skip 7.1 to 7.6<br>If response is "2 or 3", skip 7.1 to 7.5
CRF4A	*7.1*	For 2nd Health Facility: For those who were admitted and treated on an in-patient basis, record date of admission	NA	Ask the participant to show if any record of date of admission	
CRF4A	*7.2*	For 2nd Health Facility: For those who were admitted and treated on an in-patient basis, date of discharge	NA	Ask the participant to show if any record of date of discharge	
CRF4A	*7.3*	For 2nd Health Facility: For those who were admitted and treated on an in-patient basis, was the participant admitted to the ICU?	0|No<br>1|Yes	Ask the participant if they were admitted to the ICU during their admission at hospital	

CRF4A	*7.4*	For 2nd Health Facility: For those who were admitted and treated on an in-patient basis, was the participant put on ventilatory support?	0|No<br>1|Yes	Ask the participant if they were given any ventilator support during their admission at hospital	
CRF4A	*7.5*	For 2nd Health Facility: For those who were admitted and treated on an in-patient basis, did the participant require oxygen therapy?	0|No<br>1|Yes	Ask the participant if they were given any oxygen support during their admission at hospital	
CRF4A	*7.6*	For 2nd Health Facility: What was the type of medication that you were prescribed?	1|Allopathic drugs<br>2|AYUSH drugs (Ayurvedic, Unani, Siddha medications)<br>3|Home remedies/ Traditional remedies<br>4|Don't know	Ask the participant for the type of medication they received, and record their response	
CRF4A	*8*	For 2ndt Health Facility: Was the participant diagnosed to have any of the acute febrile illnesses of interest for this study? (COVID 19, Dengue, Chikungunya, Typhoid, Malaria, Japanese Encephalitis, Scrub Typhus, Leptospirosis)	0|No<br>1|Yes	Ask the participant if they were diagnosed with any AFI like COVID 19, Dengue, Chikungunya, Typhoid, Malaria, Japanese Encephalitis, Scrub Typhus, Leptospirosis	If response is "0", go to 8.37
CRF4A	*8.1*	Was a diagnosis of Covid-19 made? (For Health Facility 2)	1|Only Clinical Diagnosis<br>2|Clinical and Laboratory Diagnosis<br>3|No details available	Ask the participant if they had any diagnosis for covid 19, record the response	If response is "0 or 1 or 3", go to 8.8<br>If response is "2", go to 8.2
CRF4A	*8.2*	Covid-19 RTPCR: Result? (For Health Facility 2)	0|Not performed<br>1|Positive<br>2|Negative<br>3|Inconclusive	Ask the participant whether he/she had RTPCR test, Record the response	If response is "No", skip 8.3

CRF4A	*8.3*	Covid-19 RTPCR: Date of Testing (For Health Facility 2)	NA	Click unknown if the date is unavailable	Existing Logic:<br>Should not be a future date
CRF4A	*8.4*	Covid-19 Rapid Antigen Test: Result? (For Health Facility 2)	0|Not performed<br>1|Positive<br>2|Negative<br>3|Inconclusive	Ask the participant whether he/she had Rapid antigen test, Record the response	If response is “No”, skip 8.5
CRF4A	*8.5*	Covid-19 Rapid Antigen Test: Date of Testing (For Health Facility 2)	NA	Click unknow if the date is unavailable	Existing Logic:<br>Should not be a future date
CRF4A	*8.6*	Covid-19 ELISA: Result? (For Health Facility 2)	0|Not performed<br>1|Positive<br>2|Negative<br>3|Inconclusive	Ask the participant whether he/she had Elisa test, Record the response	If response is “No”, skip 8.7
CRF4A	*8.7*	Covid-19 ELISA: Date of Testing (For Health Facility 2)	NA	Click unknow if the date is unavailable	Existing Logic:<br>Should not be a future date
CRF4A	*8.8*	Was a diagnosis of dengue made? (For Health Facility 2)	0|Not Diagnosed<br>1|Only Clinical Diagnosis<br>2|Clinical and Laboratory Diagnosis<br>3|No details available	Ask the participant if they had any diagnosis for Dengue, record the response	If response is “0 or 1 or 3”, go to 8.21<br>If response is 2, go to 8.9
CRF4A	*8.9*	Dengue Rapid Test for NS1 Ag/IgM: Result? (For Health Facility 2)	0|Not performed<br>1|Positive<br>2|Negative<br>3|Inconclusive	Ask the participant if they had any diagnosis for Dengue, record the response	If response is “No”, skip 8.10
CRF4A	*8.10*	Dengue Rapid Test for NS1 Ag/IgM: Date of Testing (For Health Facility 2)	NA	Ask the participant whether he/she Dengue Rapid Test for NS1 [illegible]	
CRF4A	*8.11*	Dengue Rapid test for IgG: Result? (For Health Facility 2)	0|Not performed<br>1|Positive<br>2|Negative<br>3|Inconclusive	Click unknown if the date is unavailable	If response is “No”, skip 8.12

CRF4A	*8.12*	Dengue Rapid test for IgG: Date of Testing (For Health Facility 2)	NA	Ask the participant whether he/she Dengue Rapid test for IgG, Record the response	Existing Logic:<br>Should not be a future date
CRF4A	*8.13*	Dengue ELISA for NS1: Result? (For Health Facility 2)	0|Not performed<br>1|Positive<br>2|Negative<br>3|Inconclusive	Click unknown if the date is unavailable	If response is "No", skip 8.14
CRF4A	*8.14*	Dengue ELISA for NS1: Date of Testing (For Health Facility 2)	NA	Ask the participant whether he/she had Dengue ELISA for NS1, Record the response	Existing Logic:<br>Should not be a future date
CRF4A	*8.15*	For Dengue ELISA for IgM: Result? (For Health Facility 2)	0|Not performed<br>1|Positive<br>2|Negative<br>3|Inconclusive	Click unknown if the date is unavailable	If response is "No", skip 8.16
CRF4A	*8.16*	For Dengue ELISA for IgM Date of Testing (For Health Facility 2)	NA	Ask the participant whether he/she Dengue ELISA for IgM, Record the response	Existing Logic:<br>Should not be a future date
CRF4A	*8.17*	Dengue ELISA for IgG: Result? (For Health Facility 2)	0|Not performed<br>1|Positive<br>2|Negative<br>3|Inconclusive	Click unknow if the date is unavailable	If response is "No", skip 8.18
CRF4A	*8.18*	Dengue ELISA for IgG Date of Testing (For Health Facility 2)	NA	Ask the participant whether he/she Dengue ELISA for IgG, Record the response	Existing Logic:<br>Should not be a future date
CRF4A	*8.19*	Dengue RTPCR: Result? (For Health Facility 2)	0|Not performed<br>1|Positive<br>2|Negative<br>3|Inconclusive	Click unknow if the date is unavailable	If response is "No", skip 8.20

CRF4A	*8.20*	Dengue RTPCR Date of Testing (For Health Facility 2)	NA	Ask the participant whether he/she Dengue RTPCR: Result? Record the response	Existing Logic:<br>Should not be a future date
CRF4A	*8.21*	Was a diagnosis of chikungunya made? (For Health Facility 2)	0|Not diagnosed<br>1|Only Clinical Diagnosis<br>2|Clinical and Laboratory Diagnosis<br>3|No details available	Ask the participant if they had any diagnosis for chikungunya, record the response	If response is " 0 or 1 or 3", go to 8.32<br>If response is " 2", go to 8.22
CRF4A	*8.22*	Chikungunya Rapid Test for IgM: Result? (For Health Facility 2)	0|Not performed<br>1|Positive<br>2|Negative<br>3|Inconclusive	Ask the participant if they had any diagnosis for Chikungunya, record the response	If response is "No", skip 8.23
CRF4A	*8.23*	Chikungunya: Rapid Test IgM Date of Testing (For Health Facility 2)	NA	Click unknow if the date is unavailable	Existing Logic:<br>Should not be a future date
CRF4A	*8.24*	Chikungunya: Rapid test for IgG what was the result? (For Health Facility 2)	0|Not performed<br>1|Positive<br>2|Negative<br>3|Inconclusive	Ask the participant whether he/she Chikungunya Rapid test for IgG, Record the response	If response is "No", skip 8.25
CRF4A	*8.25*	Chikungunya: Rapid test for IgG Date of Testing (For Health Facility 2)	NA	Click unknow if the date is unavailable	Existing Logic:<br>Should not be a future date
CRF4A	*8.26*	For Chikungunya ELISA for IgM what was the result? (For Health Facility 2)	0|Not performed<br>1|Positive<br>2|Negative<br>3|Inconclusive	Ask the participant whether he/she had Chikungunya ELISA for IgM, Record the response	If response is "No", skip 8.27
CRF4A	*8.27*	Chikungunya ELISA for IgM Date of Testing (For Health Facility 2)	NA	Click unknow if the date is unavailable	Existing Logic:<br>Should not be a future date

CRF4A	*8.28*	Chikungunya ELISA for IgG what was the result? (For Health Facility 2)	0|Not performed<br>1|Positive<br>2|Negative<br>3|Inconclusive	Ask the participant whether he/she had Chikungunya ELISA for IgG, Record the response	If response is "No", skip 8.29
CRF4A	*8.29*	Chikungunya ELISA for IgG Date of Testing (For Health Facility 2)	NA	Click unknow if the date is unavailable	Existing Logic: Should not be a future date
CRF4A	*8.30*	Chikungunya RTPCR. Result? (For Health Facility 2)	0|Not performed<br>1|Positive<br>2|Negative<br>3|Inconclusive	Ask the participant whether he/she had Chikungunya RTPCR, Record the response	If response is "No", skip 8.31
CRF4A	*8.31*	Chikungunya RTPCR Date of Testing (For Health Facility 2)	NA	Click unknow if the date is unavailable	Existing Logic:<br>Should not be a future date
CRF4A	*8.32*	Was a diagnosis of Typhoid made? (For Health Facility 2)	0|Not diagnosed<br>1|Only Clinical Diagnosis<br>2|Clinical and Laboratory Diagnosis<br>3|No details available	Ask the participant if he/she had any diagnosis for typhoid	None
CRF4A	*8.33*	Was a diagnosis of Malaria made? (For Health Facility 2)	0|Not diagnosed<br>1|Only Clinical Diagnosis<br>2|Clinical and Laboratory Diagnosis<br>3|No details available	Ask the participant if he/she had any diagnosis for Malaria	None
CRF4A	*8.34*	was a diagnosis of Japanese Encephalitis made? (For Health Facility 2)	0|Not diagnosed<br>1|Only Clinical Diagnosis<br>2|Clinical and Laboratory Diagnosis<br>3|No details available	Ask the participant if he/she had any diagnosis for JE	None

CRF4A	*8.35*	was a diagnosis of Scrub Typhus made? (For Health Facility 2)	0|Not diagnosed<br>1|Only Clinical Diagnosis<br>2|Clinical and Laboratory Diagnosis<br>3|No details available	Ask the participant if he/she had any diagnosis for scrub typhus	None
CRF4A	*8.36*	Was a diagnosis of Leptospirosis made? (For Health Facility 2)	0|Not diagnosed<br>1|Only Clinical Diagnosis<br>2|Clinical and Laboratory Diagnosis<br>3|No details available	Ask the participant if he/she had any diagnosis for leptospirosis	None
CRF4A	*8.37*	Other's Specify for 2nd Health Facility: Was the participant diagnosed to have any other illnesses other than mentioned [illegible]	0|No<br>1|Yes	Ask the participant if he/she had diagnosed with any other illness and record the response	If response is "No", skip 8.38 and 8.39
CRF4A	*8.38*	If "Yes", Mention the name of illness diagnosed (Health Facility 2)			None
CRF4A	*8.39*	For Other illness mentioned in QS………: What was the basis of diagnosis (For Health Facility 2)	0|Not diagnosed<br>1|Only Clinical Diagnosis<br>2|Clinical and Laboratory Diagnosis<br>3|No details available		None
CRF4A	*9*	For 3rd Health Facility: Type of health facility/ practitioner accessed for treatment	1|Public facility<br>2|Private facility<br>3|Known physician (Family, social acquaintances, friends)<br>4|Health care worker (e.g., Home visit by ASHAs, ANMs)<br>5|Traditional healers/Unqualified practitioners	Ask the participant for the kind of health facility he accessed for treatment, record the response	If response is "1", skip 9.2<br>If response is "2", skip 9.1<br>If response is "3 or 4 or5", skip 9.1 and 9.2

CRF4A	*9.1*	For 3rd Health Facility: If Public facility, mention type	1|HWC / Sub Center<br>2|PHC / CHC<br>3|District Hospital<br>4|Medical College		
CRF4A	*9.2*	For 3rd Health Facility: If Private facility, mention type	1|Private Clinics<br>2|Private Nursing Home<br>3|NGO/Trust Hospital<br>4|Private hospital		
CRF4A	*9.3*	For 3rd Health Facility: Mention name and address of facility	NA		
CRF4A	*10*	For 3rd Health Facility: What type of treatment did you receive?	1|No treatment was advised<br>2| Treated as out-patient<br>3| Treated on day care basis (kept under observation for <24 hours and discharged)<br>4|Admitted for treatment (IP)	Ask the participant for the type of treatment they received, and record their response	If response is "1", skip 10.1 to 10.6<br>If response is "2 or 3", skip 10.1 to 10.5
CRF4A	*10.1*	For 3rd Health Facility: For those who were admitted and treated on an in-patient basis, record date of admission	Na	Ask the participant to show if any record of date of admission	None
CRF4A	*10.2*	For 3rd Health Facility: For those who were admitted and treated on an in-patient basis, date of discharge	Na	Ask the participant to show if any record of date of discharge	None
CRF4A	*10.3*	For 3rd Health Facility: For those who were admitted and treated on an in-patient basis, was the participant admitted to the ICU?	0|No<br>1|Yes	Ask the participant if they were admitted to the ICU during their admission at hospital	None

CRF4A	*10.4*	For 3rd Health Facility: For those who were admitted and treated on an in-patient basis, was the participant put on ventilatory support?	0|No<br>1|Yes	Ask the participant if they were given any ventilator support during their admission at hospital	None
CRF4A	*10.5*	For 3rd Health Facility: For those who were admitted and treated on an in-patient basis, did the participant require oxygen therapy?	0|No<br>1|Yes	Ask the participant if they were given any oxygen support during their admission at hospital	None
CRF4A	*10.6*	For 3rd Health Facility: What was the type of medication that you were prescribed?	1|Allopathic drugs<br>2|AYUSH drugs (Ayurvedic, Unani, Siddha medications)<br>3|Home remedies/ Traditional remedies<br>4|Don't know	Ask the participant for the type of medication they received, and record their response	None
CRF4A	*11*	For 3rd Health Facility: Was the participant diagnosed to have any of the acute febrile illnesses of interest for this study? (COVID 19, Dengue, Chikungunya, Typhoid, Malaria, Japanese Encephalitis, Scrub Typhus, Leptospirosis)	0|No<br>1|Yes	Ask the participant if they were diagnosed with any AFI like COVID 19, Dengue, Chikungunya, Typhoid, Malaria, Japanese Encephalitis, Scrub Typhus, Leptospirosis	If response is "No", go to 11.37
CRF4A	*11.1*	was a diagnosis of Covid-19 made? (For Health Facility 3)	0|Not diagnosed<br>1|Only Clinical Diagnosis<br>2|Clinical and Laboratory Diagnosis<br>3|No details available	Ask the participant if they had any diagnosis for covid 19, record the response	If response is "0 or 1 or 3", go to 11.8<br>If response is "2", go to Q11.2
CRF4A	*11.2*	Covid-19 RTPCR: Result? (For Health Facility 3)	0|Not performed<br>1|Positive<br>2|Negative<br>3|Inconclusive	Ask the participant whether he/she had RTPCR test, Record the response	If response is "No", skip 11.3

CRF4A	*11.3*	Covid-19 RTPCR: Date of Testing (For Health Facility 3)	NA	Click unknow if the date is unavailable	Existing Logic:<br>Should not be a future date
CRF4A	*11.4*	Covid-19 Rapid Antigen Test: Result (For Health Facility 3)	0|Not performed<br>1|Positive<br>2|Negative<br>3|Inconclusive	Ask the participant whether he/she had Rapid antigen test, Record the response	If response is "No", skip 11.5
CRF4A	*11.5*	Covid-19 Rapid Antigen Test: Date of Testing (For Health Facility 3)	NA	Click unknow if the date is unavailable	
CRF4A	*11.6*	Covid-19 ELISA: Result? (For Health Facility 3)	0|Not performed<br>1|Positive<br>2|Negative<br>3|Inconclusive	Ask the participant whether he/she had Elisa test, Record the response	If response is "No", skip 11.7
CRF4A	*11.7*	Covid-19 ELISA: Date of Testing (For Health Facility 3)	NA	Click unknow if the date is unavailable	Existing Logic:<br>Should not be a future date
CRF4A	*11.8*	Was a diagnosis of dengue made? (For Health Facility 3)	0|Not diagnosed<br>1|Only Clinical Diagnosis<br>2|Clinical and Laboratory Diagnosis<br>3|No details available	Ask the participant if they had any diagnosis for Dengue, record the response	If response is "0 or 1 or 3", go to 11.21<br>If response is "2", go to 11.9
CRF4A	*11.9*	Dengue Rapid Test for NS1 Ag/IgM: Result (For Health Facility 3)	0|Not performed<br>1|Positive<br>2|Negative<br>3|Inconclusive	Ask the participant whether he/she Dengue Rapid Test for NS1 Ag/IgM, Record the response	If response is "No", skip 11.10
CRF4A	*11.10*	Dengue Rapid Test for NS1 Ag/IgM: Date of Testing (For Health Facility 3)	NA	Click unknown if the date is unavailable	Existing Logic:<br>Should not be a future date
CRF4A	*11.11*	Dengue Rapid test for IgG: Result (For Health Facility 3)	0|Not performed<br>1|Positive<br>2|Negative<br>3|Inconclusive	Ask the participant whether he/she Dengue Rapid test for IgG, Record the response	If response is "No", skip 11.12

CRF4A	*11.12*	Dengue Rapid test for IgG: Date of Testing (For Health Facility 3)	NA	Click unknown if the date is unavailable	Existing Logic:<br>Should not be a future date
CRF4A	*11.13*	Dengue ELISA for NS1: Result (For Health Facility 3)	0|Not performed<br>1|Positive 2|Negative<br>3|Inconclusive	Ask the participant whether he/she had Dengue ELISA for NS1, Record the response	If response is “No”, skip 11.14
CRF4A	*11.14*	Dengue ELISA for NS1: Date of Testing (For Health Facility 3)	NA	Click unknown if the date is unavailable	
CRF4A	*11.15*	Dengue ELISA for IgM: Result (For Health Facility 3)	0|Not performed<br>1|Positive 2|Negative<br>3|Inconclusive	Ask the participant whether he/she Dengue ELISA for IgM, Record the response	If response is “No”, skip 11.16
CRF4A	*11.16*	Dengue ELISA for IgM: Date of Testing (For Health Facility 3)	NA	Click unknown if the date is unavailable	Existing Logic:<br>Should not be a future date
CRF4A	*11.17*	Dengue ELISA for IgG: Result (For Health Facility 3)	0|Not performed<br>1|Positive<br>2|Negative<br>3|Inconclusive	Ask the participant whether he/she Dengue ELISA for IgG, Record the response	If response is “No”, skip 8.18
CRF4A	*11.18*	Dengue ELISA for IgG: Date of Testing (For Health Facility 3)	NA	Click unknown if the date is unavailable	Existing Logic:<br>Should not be a future date
CRF4A	*11.19*	Dengue RTPCR: Result (For Health Facility 3)	0|Not performed<br>1|Positive<br>2|Negative<br>3|Inconclusive	Ask the participant whether he/she Dengue RTPCR: Result Record the response	If response is “No”, skip 11.20
CRF4A	*11.20*	Dengue RTPCR: Date of Testing (For Health Facility 3)	NA	Click unknown if the date is unavailable	Existing Logic:<br>Should not be a future date
CRF4A	*11.21*	Was a diagnosis of Chikungunya made? (For Health Facility 3)	0|Not diagnosed<br>1|Only Clinical Diagnosis<br>2|Clinical and Laboratory Diagnosis<br>3|No details available	Ask the participant if they had any diagnosis for chikungunya, record the response	If response is “0 or 1 or 3” go to 11.32<br>If response is 2, go to 11.22

CRF4A	*11.22*	Chikungunya: Rapid Test for IgM: Result (For Health Facility 3)	0|Not performed<br>1|Positive<br>2|Negative<br>3|Inconclusive	Ask the participant if they had any diagnosis for Chikungunya, record the response	If response is “No”, skip 11.23
CRF4A	*11.23*	Chikungunya: Rapid Test IgM Date of Testing (For Health Facility 3)	NA	Click unknown if the date is unavailable	Existing Logic:<br>Should not be a future date
CRF4A	*11.24*	Chikungunya Rapid test for IgG: Result (For Health Facility 3)	0|Not performed<br>1|Positive<br>2|Negative<br>3|Inconclusive	Ask the participant whether he/she Chikungunya Rapid test for IgG, Record the response	If response is “No”, skip 11.25
CRF4A	*11.25*	Chikungunya: Rapid test for IgG Date of Testing (For Health Facility 3)	NA	Click unknown if the date is unavailable	Existing Logic:<br>Date cannot be future
CRF4A	*11.26*	Chikungunya ELISA for IgM: Result (For Health Facility 3)	0|Not performed<br>1|Positive<br>2|Negative<br>3|Inconclusive	Ask the participant whether he/she had Chikungunya ELISA for IgM, Record the response	If response is “No”, skip 11.27
CRF4A	*11.27*	Chikungunya ELISA for IgM Date of Testing (For Health Facility 3)	NA	Click unknown if the date is unavailable	Existing Logic:<br>Future date will not be allowed
CRF4A	*11.28*	Chikungunya ELISA for IgG: Result (For Health Facility 3)	0|Not performed<br>1|Positive<br>2|Negative<br>3|Inconclusive	Ask the participant whether he/she had Chikungunya ELISA for IgG, Record the response	If response is “No”, skip Q11.29
CRF4A	*11.29*	Chikungunya ELISA for IgG Date of Testing (For Health Facility 3)	NA	Click unknown if the date is unavailable	Existing Logic: Future date will not be allowed
CRF4A	*11.30*	Chikungunya RTPCR: Result (For Health Facility 3)	0|Not performed<br>1|Positive<br>2|Negative<br>3|Inconclusive	Ask the participant whether he/she had Chikungunya RTPCR, Record the response	If response is “No”, skip Q11.31

CRF4A	*11.31*	Chikungunya RTPCR Date of Testing (For Health Facility 3)	NA	Click unknown if the date is unavailable	Existing Logic:<br>Future date will not be allowed
CRF4A	*11.32*	Was a diagnosis of Typhoid made? (For Health Facility 3)	0|Not diagnosed<br>1|Only Clinical Diagnosis<br>2|Clinical and Laboratory Diagnosis<br>3|No details available	Ask the participant if he/she had any diagnosis for typhoid	None
CRF4A	*11.33*	Was a diagnosis of Malaria made? (For Health Facility 3)	0|Not diagnosed<br>1|Only Clinical Diagnosis<br>2|Clinical and Laboratory Diagnosis<br>3|No details available	Ask the participant if he/she had any diagnosis for Malaria	None
CRF4A	*11.34*	Was a diagnosis of Scrub Typhus made? (For Health Facility 3)	0|Not diagnosed<br>1|Only Clinical Diagnosis<br>2|Clinical and Laboratory Diagnosis<br>3|No details available	Ask the participant if he/she had any diagnosis for JE	None
CRF4A	*11.35*	Was a diagnosis of Scrub Typhus made? (For Health Facility 3)	0|Not diagnosed<br>1|Only Clinical Diagnosis<br>2|Clinical and Laboratory Diagnosis<br>3|No details available	Ask the participant if he/she had any diagnosis for scrub typhus	None
CRF4A	*11.36*	Was a diagnosis of Leptospirosis made? (For Health Facility 3)	0|Not diagnosed<br>1|Only Clinical Diagnosis<br>2|Clinical and Laboratory Diagnosis<br>3|No details available	Ask the participant if he/she had any diagnosis for leptospirosis	None
CRF4A	*11.37*	Other's Specify for 3rd Health Facility: Was the participant diagnosed to have any other illnesses other than mentioned above	0|No<br>1|Yes	Ask the participant if he/she had diagnosed with any other illness and record the response	If response is “No”, skip 11.38 and 11.39

CRF4A	*11.38*	If Yes, Mention the name of illness diagnosed (Health Facility 3)			None
CRF4A	*11.39*	For Other illness mentioned in QS………: What was the basis of diagnosis (For Health Facility 3)	0|Not diagnosed<br>1|Only Clinical Diagnosis<br>2|Clinical and Laboratory Diagnosis<br>3|No details available		None
CRF4A	12	Remarks	NA		None

Form	Q No	Questions	Response options	Instruction to Data collector	Built in software logics
**Hospitalisation History at Admission**					
CRF4B.1	1	Name and address of the hospital		Enter the full name and address of the hospital where the participant was [illegible]	None
CRF4B.1	2	Was the participant admitted in ward /ICU?			None
CRF4B.1	2.1	Ward / ICU No.			None
CRF4B.1	2.2	Bed No.			None
CRF4B.1	3	*Type of health facility*	1|Public facility<br>2|Private facility	Ask the participant for the type of health facility and record the response	If response is “1”, skip 3.2<br>If response is “2”, Skip 3.1
CRF4B.1	3.1	*If Public facility, mention type*	1|HWC/ PHC / CHC<br>2|Sub Divisional / Sub District Hospital<br>3|District Hospital<br>4|Medical College		None
CRF4B.1	3.2	*If Private facility, mention type*	1|Private Nursing Home<br>2|NGO/Trust Hospital<br>3|Private hospital		None
CRF4B.1	4	Is this facility a designated COVID treatment facility	0|No<br>1|Yes<br>2|Status not Known	Ask the participant whether the hospital he was admitted, had a facility designated to Covid treatment, record the response	None
CRF4B.1	5	Was the research team member able to visit hospital	0|No<br>1|Yes		if response is "No", go to 5.1<br>If response is "Yes", skip 5.1

CRF4B.1	5.1	If “No”, state reason	NA		None
CRF4B.1	6	If “Yes”, was the research team member able to access the patient's records?	0|No<br>1|Yes		If Response is "Yes", Skip 6.1
CRF4B.1	6.1	If access to patient's records was not possible, state reasons?	NA		None
CRF4B.1	7	Date of Hospitalization	NA		Existing Logic:<br>Future date will not be allowed
CRF4B.1	7.1	Time of Hospitalization			
CRF4B.1	*8*	Fever	0|No<br>1|Yes<br>2|Details not available		If response is “0 or 2”, skip 8.1
CRF4B.1	*8.1*	Duration of Fever	NA		Existing logic:<br>Range 0 to 20
CRF4B.1	9	Cough	0|No<br>1|Yes<br>2|Details not available		If response is “0 or 2”, skip 9.1
CRF4B.1	9.1	Cough: Duration of illness	NA		Existing logic:<br>Range 0 to 20
CRF4B.1	*10*	Difficulty of breathing	1|Yes<br>2|No<br>3|Details not available		If response is “0 or 2”, skip 10.1
CRF4B.1	*10.1*	Difficulty of breathing: Duration of illness	NA		Existing logic:<br>Range 0 to 20
CRF4B.1	*11*	Running Nose / Nasal Congestion	1|Yes<br>2|No<br>3|Details not available		If response is “0 or 2”, skip 11.1
CRF4B.1	*11.1*	Running Nose / Nasal Congestion: Duration of Illness	NA		Existing logic:<br>Range 0 to 20

CRF4B.1	*12*	Sore Throat	0|No<br>1|Yes<br>2|Details not available		If response is "0 or 2", skip 12.1
CRF4B.1	*12.1*	Sore Throat: Duration of Illness	NA		Existing logic:<br>Range 0 to 20
CRF4B.1	*13*	Bleeding from any site/ blue spots on the skin/bleeding spots	0|No<br>1|Yes<br>2|Details not available		If response is "0 or 2", skip 13.1
CRF4B.1	*13.1*	Bleeding from any site/ blue spots on the skin/bleeding spots: Duration of illness	NA		Existing logic:<br>Range 0 to 20
CRF4B.1	*14*	Blood in sputum	0|No<br>1|Yes<br>2|Details not available		If response is "0 or 2", skip 14.1
CRF4B.1	*14.1*	Blood in sputum: Duration of Illness	NA		Existing logic:<br>Range 0 to 20
CRF4B.1	*15*	Blood in vomitus	0|No<br>1|Yes<br>2|Details not available		If response is "0 or 2", skip 15.1
CRF4B.1	*15.1*	Blood in vomitus: Duration of Illness	NA		Existing logic:<br>Range 0 to 20
CRF4B.1	*16*	Bleeding from Nose (epistaxis)	0|No<br>1|Yes<br>2|Details not available		If response is "0 or 2", skip 16.1
CRF4B.1	*16.1*	Bleeding from Nose (epistaxis): Duration of Illness	NA		Existing logic:<br>Range 0 to 20
CRF4B.1	*17*	Rash (Macular / Papular)	0|No<br>1|Yes<br>2|Details not available		If response is "0 or 2", skip 17.1
CRF4B.1	*17.1*	Rash (Macular / Papular): Duration of Illness	NA		Existing logic:<br>Range 0 to 20

CRF4B.1	*18*	Joint Pain	0|No<br>1|Yes<br>2|Details not available		If response is “0 or 2”, skip 18.1
CRF4B.1	*18.1*	Joint Pain: Duration of illness	NA		Existing logic:<br>Range 0 to 20
CRF4B.1	*19*	Body ache/Malaise	0|No<br>1|Yes<br>2|Details not available		If response is “0 or 2”, skip 19.1
CRF4B.1	*19.1*	Body ache/Malaise: Duration of Illness	NA		Existing logic:<br>Range 0 to 20
CRF4B.1	*20*	Headache / Pain behind eye ball	0|No<br>1|Yes<br>2|Details not available		If response is “0 or 2”, skip 20.1
CRF4B.1	*20.1*	Headache / Pain behind eye ball: Duration of Illness	NA		Existing logic:<br>Range 0 to 20
CRF4B.1	*21*	Loss of appetite/Nausea / vomiting	0|No<br>1|Yes<br>2|Details not available		If response is “0 or 2”, skip 21.1
CRF4B.1	*21.1*	Loss of appetite/Nausea / vomiting: Duration of Illness	NA		Existing logic:<br>Range 0 to 20
CRF4B.1	*22*	Diarrhea	0|No<br>1|Yes<br>2|Details not available		If response is “0 or 2”, skip 22.1
CRF4B.1	*22.1*	Diarrhea: Duration of Illness	NA		Existing logic:<br>Range 0 to 20
CRF4B.1	*23*	Abdominal Pain	0|No<br>1|Yes<br>2|Details not available		If response is “0 or 2”, skip 23.1
CRF4B.1	*23.1*	Abdominal Pain: Duration of Illness	NA		Existing logic:<br>Range 0 to 20
CRF4B.1	*24*	Loss of taste (Ageusia)	0|No<br>1|Yes<br>2|Details not available		If response is “0 or 2”, skip 24.1

CRF4B.1	*24.1*	Loss of taste (Ageusia): Duration of Illness	NA		Existing logic:<br>Range 0 to 20
CRF4B.1	*25*	Loss of smell (Anosmia)	0|No<br>1|Yes<br>2|Details not available		If response is “0 or 2”, skip 25.1
CRF4B.1	*25.1*	Loss of smell (Anosmia): Duration of Illness	NA		Existing logic:<br>Range 0 to 20
CRF4B.1	*26*	Refusal to feed (In children 5 years of age)	0|No<br>1|Yes<br>2|Details not available		If response is “0 or 2”, skip 26.1
CRF4B.1	*26.1*	Refusal to feed: Duration			Existing logic:<br>Range 0 to 20
CRF4B.1	*27*	Any other symptom	0|No<br>1|Yes<br>2|Details not available		If response is “0 or 2”, skip 27.1 and 27.2
CRF4B.1	*27.1*	Name the symptom	NA		
CRF4B.1	*27.2*	Any other Symptom: Duration of Illness	NA		Existing logic:<br>Range 0 to 20
CRF4B.1	*28*	Consciousness	1|Conscious<br>2|Unconscious<br>3|Semi-conscious<br>4|Details not available		
CRF4B.1	*29.1*	Systolic Blood Pressure (in mm Hg)	*Systolic BP…………. mmHg*	Please check the admission record and note the SBP recorded AT THE TIME OF ADMISSION<br>Click unknown if measurement unavailable	Existing Logic:<br>Range 70-200 mm Hg

CRF4B.1	*29.2*	Diastolic Blood Pressure (in mm Hg)	*Diastolic BP ………… mm Hg*<br>*Unknown*	Please check the admission record and note the DBP recorded AT THE TIME OF ADMISSION<br>Click unknown if measurement unavailable	Existing logic:<br>Range50-130 mm Hg
CRF4B.1	*30*	Temperature (select scale)	1|Celsius<br>2|Farenheit<br>3|Details not available		If details not available skip 30.1
CRF4B.1	*30.1*	Record temperature		Please check the admission record and note the temperature recorded AT THE TIME OF ADMISSION<br>Click unknown if measurement unavailable	Existing logic:<br>Range 30-104
CRF4B.1	*31*	SpO2 (click unknown if details not available)	………………. % Saturation	Please check the admission record and enter the SpO2 recorded AT THE TIME OF ADMISSION<br>Click unknown if measurement unavailable	Existing logic:<br>Range 60-100
CRF4B.1	*32*	Was the patient shifted to ICU on day of admission?	0|No<br>1|Yes<br>2|Details not available	Ask the participant if they were admitted to the ICU on the day of admission, and record the response	None
CRF4B.1	*33*	Did the patient require ventilatory support on day of admission?	0|No<br>1|Yes<br>2|Details not available	Ask the participant if they were given any ventilator support on the day of admission, and record the response	None

CRF4B.1	*34*	Was the patient on Oxygen therapy on day of admission?	0|No<br>1|Yes<br>2|Details not available	Ask the participant if they were given any oxygen support on the day of admission, and record the response	None
CRF4B.1	*35*	Was the patient on IV Fluids on day of admission?	0|No<br>1|Yes<br>2|Details not available	Ask the participant if they were given IV fluids on the day of admission, and record the response	None
CRF4B.1	*36*	Were antimicrobials started on day of admission?	0|No<br>1|Yes<br>2|Details not available		None
CRF4B.1	36.1	If "Yes", select the class(es) of antimicrobials used.	1| Penicillin (Amoxicillin / Ampicillin / Amoxi Clav)<br>2| Macrolide (Erythromycin / Azithromycin)<br>3|Cephalosporins (Cefixime / Cefuroxime / Ceftriaxone)<br>4|Chloramphenicol<br>5|Tetracyclines<br>6|Aminoglycosides (Gentamicin)<br>7|Fluroquinolones (Levofloxacin / Ofloxacin)<br>8|Anti-Malarial (Chloroquine)<br>9|Antivirals (Remdesivir/ Faripiravir)<br>10|Others		None
CRF4B.1	37	Were steroids started on day of admission?	0|No<br>1|Yes<br>2|Details not available		None
CRF4B.1	38	Were anticoagulants started on day of admission?	0|No<br>1|Yes<br>2|Details not available		None
CRF4B.1	39	Remarks			None

Form	Q No	Questions	Response options	Instruction to Data collector	Built in software logics
**Hospitalisation course and investigation**					
CRF4B.2	1	What is the present condition of the participant?	1|Discharged<br>2|Continued hospitalisation<br>3|Referred<br>4|LAMA<br>5|Death<br>6|Unknown	Record the present condition of the participant	Existing Logic:<br>If response is "Referred", complete form and trigger Form 4B.1<br>If response is "Discharged", trigger outcome Form 4B.4 after filling this form<br>If response is "Death", fill Form 4C after completing this form"<br>If response is "Referred", go to 1.1 and 1.2<br>If response is other than "Referred" (1 or 2 or 4 or 5 or 6), go to 2
CRF4B.2	1.1	If referred to another hospital, Reason	1|Better medical facilities (Higher level health facility)<br>2|Step down care till recovery (lower-level health facility)<br>3|Health facility near home<br>4|Financial reasons<br>5|Unknown	Record the reason for referral	None
CRF4B.2	1.2	Name and address of the referral hospital	NA	mention the name and address of the hospital	None
CRF4B.2	2	Was the research team able to access the participant's records?	0|No<br>1|Yes		If response is "Yes", go to 3<br>If response is "No", go to 12, end Form
CRF4B.2	3	Between last date of Follow till date was patient given Oxygen	0|No<br>1|Yes<br>2|Details not available	Ask the participant if he/she had been given oxygen during in the days of hospital admission	None

CRF4B.2	4	Between last date of Follow till date was patient on IV Fluids	0|No<br>1|Yes<br>2|Details not available	Ask the participant if he/she had been given IV Fluids during in the days of hospital admission	None
CRF4B.2	5	Between last date of Follow till date was the patient admitted in ICU	0|No<br>1|Yes<br>2|Details not available	Ask the participant if he/she was admitted in ICU	If response is "0 or 2", skip 6
CRF4B.2	6	Between last date of Follow till date was patient put on Ventilator	0|No<br>1|Yes<br>2|Details not available	Ask the participant if he/she was admitted in ventilator	None
CRF4B.2	7	Between last date of Follow till date was patient on Inotropes	0|No<br>1|Yes<br>2|Details not available		None
	8	Is report of Complete Blood Count available?	0|No<br>1|Yes	Ask the participant if the CBC reports available,<br>if "Yes" record the readings	If response is "No", go to 9
CRF4B.2	8.1	Complete Blood Count			None
		Haemoglobin (in g/dL)	.... gm/dl		Existing Logic:<br>Range 3 to 15
		WBC Count	.... cells / mm3		Existing Logic:<br>Range 900-25000
		Differential blood count- % of Lymphocytes	.... %		Existing Logic:<br>Range 20-40
		Differential blood count- % of Neutrophils	.... %		Existing Logic:<br>Range 20-95
		Differential blood count- % of Basophils	.... %		Existing Logic:<br>Range 00-05
		Differential blood count- % of Eosinophils	.... %		Existing Logic:<br>Range 00-20

		Differential blood count- % of Macrophages	………………. %		Existing Logic:<br>Range 00-50
		Platelet count (mention absolute number)	……………….. X 10^9/l		Existing Logic:<br>Range 5000-600000
CRF4B.2	9	Is report of Liver function test available?	1|Yes 0|No		Existing Logic:<br>if response is" No", go to 10
CRF4B.2	9.1	Liver function test		If liver function test available, record the readings	
		Total Bilirubin	mg/dl		Existing Logic:<br>Range0.1-50
		Indirect Bilirubin	mg/dl		Existing Logic:<br>Range0-45
		Direct Bilirubin	mg/dl		Existing Logic:<br>Range 0-5
		SGOT (in mg/dl)	u/L		Existing Logic:<br>Range 2-100
		SGPT (in mg/dl)	u/L		Existing Logic:<br>Range 2-100
		Alkaline Phosphatase	u/L		Existing Logic:<br>Range10-250
		Total Protein	gm/dl		Existing Logic:<br>Range 1-20
		Serum Albumin	gm/dl		Existing Logic:<br>Range 1-10
CRF4B.2	10	Is report of Kidney function test available?	0|No<br>1|Yes	If liver function test available, record the readings	If response is “No” , go to 11
CRF4B.2	10.1	KFT			

		Blood Urea	mg/dl		Existing Logic:<br>Range 0-50
		Serum Creatinine	mg/dl		Existing Logic:<br>Range 1-10
CRF4B.2	11	Was the participant diagnosed to have any of the acute febrile illnesses of interest for this study?	0|No<br>1|Yes	Ask the participant if they were diagnosed with any AFI like COVID 19, Dengue, Chikungunya, Typhoid, Malaria, Japanese Encephalitis, Scrub Typhus, Leptospirosis	If response is "No", go to 11.9
CRF4B.2	11.1	Was a diagnosis of COVID-19 made?	0|Not Diagnosed<br>1|Only Clinical Diagnosis<br>2|Clinical and Laboratory Diagnosis<br>3|No details available	Ask the participant if they had any diagnosis for covid 19, record the response	If response is "2", go to 11.1.1<br>If response is "0 or 1 or 3", go to 11.2
CRF4A	11.1.1	For Covid-19 RTPCR: Result	0|Not performed<br>1|Positive<br>2|Negative<br>3|Inconclusive	Ask the participant whether he/she had RTPCR test, Record the response	If response is "1 or 2 or 3", go to 11.1.1.1<br>If response is "0", go to 11.1.2
CRF4A	11.1.1.1	COVID-19 RTPCR 1: Date of Testing	NA	Click unknown if the data is unavailable	Existing Logic:<br>Future date will not be allowed
CRF4A	11.1.2	Covid-19 Rapid Antigen Test: Result?	0|Not performed<br>1|Positive<br>2|Negative<br>3|Inconclusive	Ask the participant whether he/she had Rapid antigen test, Record the response	If response is "1 or 2 or 3", go to 11.1.2.1<br>If response is "0", go to 11.1.3
CRF4A	11.1.2.1	Covid-19 Rapid Antigen Test: Date of Testing	NA	Click unknown if the data is unavailable	Existing Logic:<br>Future date will not be allowed
CRF4A	11.1.3	Covid-19 ELISA: Result?	0|Not performed<br>1|Positive<br>2|Negative<br>3|Inconclusive	Ask the participant whether he/she had Elisa test, Record the response	If response is "1 or 2 or 3", go to 11.1.3.1<br>If response is "0", go to 11.1.4

CRF4A	11.1.3.1	Covid-19 ELISA: Date of Testing	NA	Click unknow if the data is unavailable	Existing Logic:<br>Future date will not be allowed
CRF4B.2	11.2	Was the diagnosis of dengue made?	0|Not diagnosed<br>1|Only Clinical Diagnosis<br>2|Clinical and Laboratory Diagnosis<br>3|No details available	Ask the participant if they had any diagnosis for Dengue, record the response	If response is “0 or 1 or 3”, , go to 11.3
CRF4B.2	11.2.1	Dengue Rapid Test for NS1 Ag/IgM: Result?	0|Not performed<br>1|Positive<br>2|Negative<br>3|Inconclusive	Ask the participant whether he/she Dengue Rapid Test for NS1 Ag/IgM, Record the response	If response is “0”, go to 11.2.2
CRF4B.2	11.2.1.1	Dengue Rapid Test for NS1 Ag/IgM: Date of Testing	NA	Click unknown if the data is unavailable	Existing Logic:<br>Future date will not be allowed
CRF4B.2	11.2.2	Dengue Rapid test for IgG: Result	0|Not performed<br>1|Positive<br>2|Negative<br>3|Inconclusive	Ask the participant whether he/she Dengue Rapid test for IgG, Record the response	If response is “0”, go to 11.2.3
CRF4B.2	11.2.2.1	Dengue Rapid test for IgG: Date of Testing	NA	Click unknown if the data is unavailable	Existing Logic:<br>Future date will not be allowed
CRF4B.2	11..2.3	Dengue ELISA for NS1: Result?	0|Not performed<br>1|Positive<br>2|Negative<br>3|Inconclusive	Ask the participant whether he/she had Dengue ELISA for NS1, Record the response	If response is “0”, go to 11.2.4
CRF4B.2	11.2.3.1	Dengue ELISA for NS1: Date of Testing	NA	Click unknown if the data is unavailable	
CRF4B.2	11.2.4	Dengue ELISA for IgM: Result?	0|Not performed<br>1|Positive<br>2|Negative<br>3|Inconclusive	Ask the participant whether he/she Dengue ELISA for IgM, Record the response	If response is “0”, go to 11.2.5
CRF4B.2	11.2.4.1	For Dengue ELISA for IgM Date of Testing	NA	Click unknown if the data is unavailable	

CRF4B.2	11.2.5	Dengue ELISA for IgG: Result?	0|Not performed<br>1|Positive<br>2|Negative<br>3|Inconclusive	Ask the participant whether he/she Dengue ELISA for IgG, Record the response	If response is "0", go to 11.2.6
CRF4B.2	11.2.5.1	Dengue ELISA for IgG: Date of Testing	NA	Click unknown if the data is unavailable	
CRF4B.2	11.2.6	Dengue RTPCR: Result?	0|Not performed<br>1|Positive<br>2|Negative<br>3|Inconclusive	Ask the participant whether he/she Dengue RTPCR: Result? Record the response	If response is "0", go to 11.3
CRF4B.2	11.2.6.1	Dengue RTPCR: Date of Testing	NA	Click unknown if the data is unavailable	
CRF4B.2	11.3	Was a diagnosis of chikungunya made?	0|Not diagnosed<br>1|Only Clinical Diagnosis<br>2|Clinical and Laboratory Diagnosis<br>3|No details available	Ask the participant if they had any diagnosis for chikungunya, record the response	If response is "0 or 1 or 2 or 3", go to 11.4
CRF4B.2	11.3.1	Chikungunya Rapid Test for IgM: Result? (For Health Facility 1)	0|Not performed<br>1|Positive<br>2|Negative<br>3|Inconclusive	Ask the participant if they had any diagnosis for Chikungunya, record the response	If response is "0", go to 11.3.2
CRF4B.2	11.3.1.1	Chikungunya: Rapid Test IgM: Date of Testing (For Health Facility 1)	NA	Click unknown if the data is unavailable	Existing Logic:<br>Future date will not be allowed
CRF4B.2	11.3.2	Chikungunya Rapid test for IgG: Result?	0|Not performed<br>1|Positive<br>2|Negative<br>3|Inconclusive	Ask the participant whether he/she Chikungunya Rapid test for IgG, Record the response	If response is "0", go to 11.3.3
CRF4B.2	11.3.2.1	Chikungunya: Rapid test for IgG Date of Testing	NA	Click unknown if the data is unavailable	Existing Logic:<br>Future date will not be allowed

CRF4B.2	11.3.3	Chikungunya ELISA for IgM: Result?	0|Not performed<br>1|Positive<br>2|Negative<br>3|Inconclusive	Ask the participant whether he/she had Chikungunya ELISA for IgM, Record the response	If response is “0”, go to 11.3.4
CRF4B.2	11.3.3.1	Chikungunya ELISA for IgM Date of Testing	NA	Click unknown if the data is unavailable	Existing Logic:<br>Future date will not be allowed
CRF4B.2	11.3.4	Chikungunya ELISA for IgG: Result	0|Not performed 1|Positive<br>2|Negative 3|Inconclusive	Ask the participant whether he/she had Chikungunya ELISA for IgG, Record the response	If response is “0”, go to 11.3.5
CRF4B.2	11.3.4.1	Chikungunya ELISA for IgG: Date of Testing	NA	Click unknown if the data is unavailable	Existing Logic:<br>Future date will not be allowed
CRF4B.2	11.3.5	For Chikungunya RTPCR. Result?	0|Not performed<br>1|Positive<br>2|Negative<br>3|Inconclusive	Ask the participant whether he/she had Chikungunya RTPCR, Record the response	If response is “0”, go to 11.3.6
CRF4B.2	11.3.5.1	Chikungunya RTPCR Date of Testing	NA	Click unknown if the data is unavailable	Existing Logic:<br>Future date will not be allowed
CRF4B.2	11.4	Was a diagnosis of Typhoid made?	0|Not diagnosed<br>1|Only Clinical Diagnosis<br>2|Clinical and Laboratory Diagnosis<br>3|No details available	Ask the participant if he/she had any diagnosis for typhoid	
CRF4B.2	11.5	Was a diagnosis of malaria made?	0|Not diagnosed<br>1|Only Clinical Diagnosis<br>2|Clinical and Laboratory Diagnosis<br>3|No details available	Ask the participant if he/she had any diagnosis for Malaria	
CRF4B.2	11.6	Was a diagnosis of Japanese Encephalitis made?	0|Not diagnosed<br>1|Only Clinical Diagnosis<br>2|Clinical and Laboratory Diagnosis<br>3|No details available	Ask the participant if he/she had any diagnosis for JE	

CRF4B.2	11.7	Was a diagnosis of Scrub Typhus made?	0|Not diagnosed<br>1|Only Clinical Diagnosis<br>2|Clinical and Laboratory Diagnosis<br>3|No details available	Ask the participant if he/she had any diagnosis for scrub typhus	
CRF4B.2	11.8	Was a diagnosis of Leptospirosis made?	0|Not diagnosed<br>1|Only Clinical Diagnosis<br>2|Clinical and Laboratory Diagnosis<br>3|No details available	Ask the participant if he/she had any diagnosis for leptospirosis	
CRF4B.2	11.9	Were you diagnosed with any other AFI	0|No<br>1|Yes	Ask the participant if he/she had diagnosed with any other illness and record the response	If response is "Yes" then skip 11.9.1 and 11.9.2
CRF4B.2	11.9.1	If any other AFI was diagnosed, please specify the name of the condition			
CRF4B.2	11.9.2	For Other illness mentioned in QS………: What was the basis of diagnosis	0|Not diagnosed<br>1|Only Clinical Diagnosis<br>2|Clinical and Laboratory Diagnosis<br>3|No details available		
CRF4B.2	12	Remarks			

Form	Q No	Questions	Response options	Instruction to Data collector	Built in software logics
**Hospital Discharge**					
CRF4B.3	*1*	*Date of Discharge*		Record the date of discharge from the records	None
CRF4B.3	*2*	*Time Discharge*		Record the time of discharge from the records	None
CRF4B.3	*3*	Is any medical record available to extract details of hospitalization	*1|Hospital files*<br>*2|Outpatient clinic chart*<br>*3|Autopsy Report*<br>*4|Death certificate*<br>*5|No record available*	Ask the participant if they have any medical records available to extract the hospitalization details	If response is “5”, go to 9
CRF4B.3	*3.1*	If “Yes”, Upload photo	*Photo*	If Hospitalization record available, click the pick and upload the reports	None
CRF4B.3	*4*	*Were hospital visits made by team prior to*	0|No<br>1|Yes		None
CRF4B.3	*5*	*Photo of discharge slip*	*Photo*	if available, Upload the photo of discharge slip	None
CRF4B.3	*6*	*Diagnosis as per discharge slip*	*1|COVID 19 2|Dengue*<br>*3|Chikungunya*<br>*4|Typhoid 5|Malaria*<br>*6|Japanese Encephalitis*<br>*7|Scrub Typhus*<br>*8|Leptospirosis*<br>*9|Others*	Check the reports and record	None
CRF4B.3	*6.1*	*If Others on Diagnosis as per discharge slip*			None

CRF4B.3	*7*	*Underlying Condition if any*	*1|Anemia 2|Malnutrition*<br>*3|Heart Disease,*<br>*4|Liver Disease*<br>*5|Lung Disease*<br>*6|Neurological or Neuromuscular Disease*<br>*7|Gastrointestinal Disease,*<br>*8|Autoimmune disorder*<br>*9|Cancer or Tumor*<br>*10|Diabeles mellitus*<br>*11|Renal failure*<br>*12|Other*		None
CRF4B.3	*7.1*	*Others for Underlying Condition*	*NA*		None
CRF4B.3	*8*	*Is the patient discharged on antimicrobials?*	0|No<br>1|Yes		If response is "0", go to 9
CRF4B.3	8.1	*If "Yes", select the class(es) of antimicrobials used.*	1|Penicillin (Amoxicillin / Ampicillin / Amoxi Clav)<br>2|Macrolide (Erythromycin / Azithromycin)<br>3|Cephalosporins (Cefixime / Cefuroxime / Ceftriaxone)<br>4|Chloramphenicol<br>5|Tetracyclines<br>6|Aminoglycosides (Gentamicin)<br>7|Fluroquinolones (Levofloxacin / Ofloxacin)<br>8|Anti-Malarial (Chloroquine)<br>9|Antivirals (Remdesivir/ Faripiravir)<br>10|Others		None
CRF4B.3	8.2	*Others for Antimicrobials [illegible]*	*NA*		
CRF4B.3	9	Remarks		None	None

Form	Q No	Questions	Response options	Instruction to Data collector	Built in software logics
**Death Form**					
CRF4C	*1*	Individual ID	*Auto filled*		
CRF4C	*2*	Date of death		Record the date of death from the records	None
CRF4C	*3*	Time of death		Record the time of death e from the records	None
CRF4C	*4*	Place of death	*1|Home*<br>*2|Health facility*<br>*3|During Transit*		None
CRF4C	*5*	Is death certificate or any medical record available to extract cause of death?	*1|Hospital files*<br>*2|Outpatient clinic chart*<br>*3|Autopsy Report*<br>*4|Death certificate*<br>*5|No record available*		If no record available, skip all and go to remarks
CRF4C	*6*	If "Yes", Upload photo	*Photo*		None
CRF4C	*7*	Immediate Cause of Death (as per death summary)	*1|COVID*<br>*2|Dengue*<br>*3|Chikungunya*<br>*4|Typhoid*<br>*5|Malaria*<br>*6|Japanese Encephalitis*<br>*7|Scrub typhus*<br>*8|Leptospirosis*<br>*9|Other, Specify*		None
CRF4C	*7.1*	Others for Immediate Cause of Death	*NA*		None

CRF4C	*8*	Underlying Cause of Death (as per death summary)	*1|Anemia*<br>*2|Malnutrition*<br>*3|Heart Disease*<br>*4|Liver Disease*<br>*5|Lung Disease,*<br>*6|Neurological or Neuromuscular Disease*<br>*7|Gastrointestinal Disease*<br>*8| Autoimmune disorder*<br>*9|Cancer / Tumor*<br>*10|Diabeles mellitus*<br>*11|Renal failure*<br>*12|Other*		None
CRF4C	8.1	Others for Underlying Cause of Death	*NA*		None
Go to Verbal Autopsy form for every death irrespective the death took place at home / during transit / hospital					

Form	Q No	Questions	Response options	Instruction to Data collector	Built in software logics
**Serosurvey Sociodemographic Information**					
CRF5A	*1*	Education (enter completed years of education in formal [illegible]	NA	If participant is less than 5 years, enter "0"	None
CRF5A	*2*	Occupation<br>(The Interviewer should ask this question in an open-ended manner: “What is your main occupation?”. The interviewer then selects the category that they think best applies,<br>and confirms it with the respondent.)	1|Student<br>2|Agriculture/ Fish breeding/ Poultry/ Farming/ Animal Rearing<br>3|Government Job excluding health providers<br>4|Private job excluding health providers<br>5|Professional (self-employed) excluding health providers<br>6|Business / enterprise/industry<br>7|Daily labour/ labour/ NREGA/ other contract work<br>8|Shop keeper/trader/property dealer/ vendor (small enterprise and mobile shops)<br>9|Domestic work<br>10|skilled labour (ex: sewing/ mechanic)<br>11|Retired<br>12|Unemployed (> 18 years and not working)<br>13|Housewife<br>14|community health workers (ANM/ASHA/AWW)<br>15|Health care providers (RMP/Doctor/Nurse)<br>16|Don't Know	if participant is less than 5 years, opt option "16"	None

CRF5A	*3*	Do you currently use smoke forms of tobacco on a daily basis, less than daily, or not at all?	1|Not at all<br>2|Less than once a week<br>3|Less than daily but more than once a week<br>4|On a daily basis	Ask the participant to think of any smoke form of tobacco products that he/she is using currently. Read out the options and record the response Try to ensure privacy of the respondent while asking this question. Please record the answer as reported by respondent.	This question will be skipped for less than 18 years of age
CRF5A	4	Do you currently use smokeless tobacco on a daily basis, less than daily, or not at all?	1|Not at all<br>2|Less than once a week<br>3|Less than daily but more than once a week<br>4|On a daily basis	(Ask the participant to think of any smokeless tobacco products that he/she is using currently. Read out the options and record the response)<br>Try to ensure privacy of the respondent while asking this question. Please record the answer as reported by respondent.	This question will be skipped for less than 18 years of age
CRF5A	5	Do you consume alcohol?<br>Ask the participant to think of any drink that contains alcohol, with the exception of alcohol-based medication that is taken due to health reasons or alcohol consumed for religious reasons. Read out the options and record the response) Try to ensure privacy of the respondent while asking this question. Please record the answer as reported by respondent.	1|Not at all<br>2|Less than once a week<br>3|Less than daily but more than once a week<br>4|On a daily basis	Ask the participant to think of any drink that contains alcohol, with the exception of alcohol-based medication that is taken due to health reasons or alcohol consumed for religious reasons. Read out the options and record the response)<br>Try to ensure privacy of the respondent while asking this question. Please record the answer as reported by respondent.	This question will be skipped for less than 18 years of age

CRF5A	6	Were you ever diagnosed to have covid19 in the past?	0|No<br>1|Yes	Ask the participant whether he had diagnosed with covid 19 in the past	IF response is "No", date of covid diagnosis will be skipped
CRF5A	6.1	If “Yes”, Diagnosed to have Covid in the past, please mention date of diagnosis			Existing Logic: Date should be 21 days before the day of visit
	7	Remarks			

Form	Q No	Questions	Response options	Instruction to Data collector	Built in software logics
**Immunization History**					
CRF5B		*Section 1 -Immunization history from children under 5 years of age (only at baseline and endline)*			Questions in Section 1 will be skipped if age is 5 or above.<br>Form opens only at baseline and endline
CRF5B	1	*Has the participant received any vaccination since birth*	0|No<br>1|Yes		If response is "No", skip all questions and go to Remarks.
CRF5B	*2*	*Source of information on vaccination*	1|Vaccination card<br>2|Caregiver report	The vaccinations would have been recorded on a health card given to the parents, which is the main source of information about childhood vaccination. If the respondent reports having a card for the child, probe "May I see it?" Or check with child's medical record.<br>Check and record the response)	If response is "2", Skip to 3<br>If response is "1", go to 2.1
CRF5B	*2.1*	*If vaccine card available, upload first photograph of the card*	NA	Instruction: Take Multiple pics if there are more than one page on the vaccine card	None
CRF5B	*2.2*	*If vaccine card available, upload second photograph of the card*	NA	Instruction: Take Multiple pics if there are more than one page on the vaccine card	None
CRF5B	*2.3*	*If vaccine card available, upload third photograph of the card*	NA	Instruction: Take Multiple pics if there are more than one page on the vaccine card	None
CRF5B	*2.4*	*If vaccine card available, upload fourth photograph of the card*	NA	Instruction: Take Multiple pics if there are more than one page on the vaccine card	None

CRF5B	***3***	***BCG birth dose***			
CRF5B	*3.1*	*BCG Birth dose Status*<br>*(Check in the report and record the response)*	0|No<br>1|Yes	Check in the vaccination card and record the response	If response is "No", go to 4<br>If response is "Yes", go to 3.3.
CRF5B	3.2	*BCG Birth dose Date of administration.*	NA	Click unknown if date is not known or illegible on the cards	Date should not be future<br>Date should not be before DOB
CRF5B	***4***	***OPV 0 dose***			
CRF5B	*4.1*	*OPV 0 dose: Status*<br>*(Check in the report and record the response)*	0|No<br>1|Yes	Check in the vaccination card and record the response	If response is "No", go to 5.<br>If response is "Yes", go to 4.2.
CRF5B	*4.2*	*OPV 0: Date of administration. Enter unknown if date is not known or illegible on the card (Check the report for the date)*	NA	Click unknown if date is not known or illegible on the cards	Date should not be future<br>Date should not be before DOB
CRF5B	***5***	***OPV 1st dose***			
CRF5B	*5.1*	*OPV 1st Dose Status*<br>*(Check in the report and record the response)*	0|No<br>1|Yes	Check in the vaccination card and record the response	If response is "No", go to 6<br>If response is "Yes", go to 5.2.
CRF5B	5.2	*OPV 1 Dose Date of administration. Enter unknown if date is not known or illegible on the card*<br>*(Check the report for the date)*	NA	Click unknown if date is not known or illegible on the cards	Date should not be future<br>Date should be later than OPV0 dose
CRF5B	***6***	***OPV 2nd dose***			
CRF5B	*6.1*	*OPV 2nd Dose Status*<br>*(Check in the report and record the response)*	0|No 1|Yes	Check in the vaccination card and record the response	If response is "No", go to 7.<br>If response is "Yes", go to 6.2.

CRF5B	*6.2*	*OPV 2nd Dose: Date of administration. Enter unknown if date is not known or illegible on the card (Check the report for the date)*	NA	Click unknown if date is not known or illegible on the cards	Date should not be a future date. Date should be later than OPV 1 dose.
CRF5B	***7***	***OPV 3rd dose***			
CRF5B	*7.1*	*OPV 3rd Dose Status (Check in the report and record the response)*	0|No<br>1|Yes	Check in the vaccination card and record the response	If response is "No", go to 8<br>If response is "Yes", go to 7.2.
CRF5B	7.2	*OPV 3rd Dose Date of administration. Enter unknown if date is not known or illegible on the card (Check the report for the date)*	NA	Click unknown if date is not known or illegible on the cards	Date should not be future<br>Date should be later than OPV 2 dose.
CRF5B	***8***	***OPV 1st booster***			
CRF5B	*8.1*	*OPV 1st Booster Status (Check in the report and record the response)*	0|No<br>1|Yes	Check in the vaccination card and record the response	If response is "No", go to 9<br>If response is "Yes", go to 8.2.
CRF5B	*8.2*	*OPV 1st Booster Date of administration. Enter unknown if date is not known or illegible on the card (Check the report for the date)*	NA	Click unknown if date is not known or illegible on the cards	Date should not be future.<br>Date should be later than OPV 3 dose.
CRF5B	***9***	***OPV last SIA round***			
CRF5B	*9.1*	*OPV last SIA Round Given?*	0|No<br>1|Yes	Check in the vaccination card and record the response	If response is "No", go to 10.<br>If response is "Yes", go to 9.2.
CRF5B	*9.2*	*OPV last SIA Date of administration. Enter unknown if date is not known or illegible on the card*	NA	Click unknown if date is not known or illegible on the cards	Date should not be future<br>Date should not be before DOB

CRF5B	***10***	**IPV (Injectable Polio Vaccine) dose 1**			
CRF5B	*10.1*	*IPV (Injectable Polio Vaccine) dose 1 Status (Check in the report and record the response)*	0|No<br>1|Yes	Check in the vaccination card and record the response	If response is "No", go to 11.<br>If response is "Yes", go to 10.2.
CRF5B	10.2	*IPV (Injectable Polio Vaccine) dose 1 Date of administration. Enter unknown if date is not known or illegible on the card (Check the report for the date)*	NA	Click unknown if date is not known or illegible on the cards	Date should not be future.<br>Date should not be before DOB
CRF5B	***11***	**IPV (Injectable Polio Vaccine) dose 2**			
CRF5B	*11.1*	*IPV (Injectable Polio Vaccine) dose 2 Status (Check in the report and record the response)*	0|No<br>1|Yes	Check in the vaccination card and record the response	If response is "No", go to 12.<br>If response is “Yes”, go to 11.2.
CRF5B	*11.2*	*IPV (Injectable Polio Vaccine) dose 2 Date of administration. Enter unknown if date is not known or illegible on the card (Check the report for the date)*	NA	lick unknown if date is not known or illegible on the cards	Date should not be a future date.<br>Date should be later than IPV 1
CRF5B	***12***	**Pentavalent Vaccine dose 1**			
CRF5B	12.1	*Pentavalent Vaccine dose 1 Status (Check in the report and record the response)*	0|No 1<br>|Yes	Check in the vaccination card and record the response	Existing Logic in software:<br>If response is “No”, go to 13.<br>If response is “Yes”, go to 12.2.
CRF5B	*12.2*	*Pentavalent Vaccine dose 1 Date of administration. Enter unknown if date is not known or illegible on the card (Check the report for the date)*	NA	Click unknown if date is not known or illegible on the cards	Date should not be a future date.<br>Date should not be before DOB

CRF5B	**13**	**Pentavalent Vaccine dose 2**			
CRF5B	*13.1*	*Pentavalent Vaccine dose 2 Status (Check in the report and record the response)*	0|No<br>1|Yes	Check in the vaccination card and record the response	If response is “No”, go to 14.<br>If response is “Yes”, go to 13.2.
CRF5B	*13.2*	*Pentavalent Vaccine dose 2 Date of administration. Enter unknown if date is not known or illegible on the card (Check the report for the date)*	NA	Click unknown if date is not known or illegible on the car ds	Date should not be future.<br>Date should be later than Pentavalent dose 1
CRF5B	**14**	***Pentavalent Vaccine dose 3***			
CRF5B	*14.1*	*Pentavalent Vaccine dose 3 Status (Check in the report and record the response)*	0|No<br>1|Yes	Check in the vaccination card and record the response	If response is “No”, go to 15.<br>If response is “Yes”, go to 14.2.
CRF5B	*14.2*	*Pentavalent Vaccine dose 3 Date of administration. Enter unknown if date is not known or illegible on the card (Check the report for the date)*	NA	Click unknown if date is not known or illegible on the cards	Date should not be future.<br>Date should be later than Pentavalent dose 3
CRF5B	***15***	**Rotavirus Vaccine dose 1**			
CRF5B	*15.1*	*Rotavirus Vaccine dose 1 Status (Check in the report and record the response)*	0|No<br>1|Yes	Check in the vaccination card and record the response	If response is “No”, go to 16<br>If response is “Yes”, go to 15.2.
CRF5B	15.2	*Rotavirus Vaccine dose 1 Date of administration. Enter unknown if date is not known or illegible on the card (Check the report for the date)*	NA	Click unknown if date is not known or illegible on the cards	Date should not be a future date.<br>Date should not be before DOB
**CRF5B**	***16***	***Rotavirus Vaccine dose 2***			
CRF5B	*16.1*	*Rotavirus Vaccine dose 2 Status (Check in the report and record the response)*	0|No<br>1|Yes	Check in the vaccination card and record the response	If response is “No”, go to 17.<br>If response is “Yes”, go to 16.2.

CRF5B	*16.2*	*Rotavirus Vaccine dose 2 Date of administration. Enter unknown if date is not known or illegible on the card (Check the report for the date)*	NA	Click unknown if date is not known or illegible on the cards	Date should not be a future date.<br>Date should not be before DOB
CRF5B	***17***	***Rotavirus Vaccine dose 3***			
CRF5B	17.1	*Rotavirus Vaccine dose 3 Status (Check in the report and record the response)*	0|No<br>1|Yes	Check in the vaccination card and record the response	If response is “No”, go to 18.<br>If response is “Yes”, go to 17.2.
CRF5B	*17.2*	*Rotavirus Vaccine dose 3 Date of administration. Enter unknown if date is not known or illegible on the card (Check the report for the date)*	NA	Click unknown if date is not known or illegible on the cards	Date should not be a future date.<br>Date should be later than Pentavalent dose 3
CRF5B	***18***	***JE Vaccine dose 1***			
CRF5B	*18.1*	*JE Vaccine dose 1 Status (Check in the report and record the response)*	0|No<br>1|Yes	Check in the vaccination card and record the response	If response is “No”, go to "19.<br>If response is “Yes”, go to 18.2.
CRF5B	*18.2*	*JE Vaccine dose 1 Date of administration. Enter unknown if date is not known or illegible on the card (Check the report for the date)*	NA	Click unknown if date is not known or illegible on the cards	Date should not be a future date.<br>Date should not be before DOB
CRF5B	**19**	***JE Vaccine dose 2***			
CRF5B	*19.2*	*JE Vaccine dose 2 Status (Check in the report and record the response)*	0|No<br>1|Yes	Check in the vaccination card and record the response	If response is “No”, go to 20.<br>If response is “Yes”, go to 19.2.
CRF5B	*19.2*	*JE Vaccine dose 2 Date of administration. Enter unknown if date is not known or illegible on the card (Check the report for the date)*	NA	Click unknown if date is not known or illegible on the cards	Existing Logic:<br>Date should not be a future date<br>Date should be later than Pentavalent dose 3

CRF5B	***20***	***DPT booster dose 1***			
CRF5B	*20.1*	*DPT booster dose 1 Status (Check in the report and record the response)*	0|No<br>1|Yes	Check in the vaccination card and record the response	If response is "No", go to 21.<br>If response is "Yes", go to 20.2.
CRF5B	20.2	*DPT booster dose 1 Date of administration.*	NA	Click unknown if date is not known or illegible on the cards	Date should not be a future date.<br>Date should not be before DOB
CRF5B	***21***	***DPT booster dose 2***			
CRF5B	*21.1*	*DPT booster dose 2 Status*	0|No<br>1|Yes	Check in the vaccination card and record the response	If response is "No", go to 22.<br>If response is "Yes", go to 21.2.
CRF5B	*21.2*	*DPT booster dose 2 Date of administration. E*	NA	Click unknown if date is not known or illegible on the cards	
CRF5B	***22***	***Measles Vaccine dose 1***			
CRF5B	22.1	*Measles Vaccine dose 1 Status*	0|No<br>1|Yes	Check in the vaccination card and record the response	If response is "No", go to 23.<br>If response is "Yes", go to 22.2.
CRF5B	*22.2*	*Measles Vaccine dose 1 Date of administration.*	NA	Click unknown if date is not known or illegible on the cards	Date should not be a future date.<br>Date should not be before DOB
CRF5B	***23***	***Measles Vaccine dose 2***			
CRF5B	*23.1*	*Measles Vaccine dose 2 Status (Check in the report and record the response)*	0|No<br>1|Yes	Check in the vaccination card and record the response	If response is "No", go to 24.<br>If response is "Yes", go to 23.2.
CRF5B	*23.2*	*Measles Vaccine dose 2 Date of administration.*	NA	Click unknown if date is not known or illegible on the cards	Date should not be a future date.<br>Date should not be before DOB
CRF5B	***24***	***MR Vaccine dose 1***			
CRF5B	24.1	*MR Vaccine dose 1 Status*	0|No<br>1|Yes	Check in the vaccination card and record the response	If response is "No", go to 25.<br>If response is "Yes", go to 24.2.
CRF5B	*24.2*	*MR Vaccine dose 1 Date of administration. E*	NA	Click unknown if date is not known or illegible on the cards	Date should not be a future date.<br>Date should not be before DOB

CRF5B	***25***	***MR Vaccine dose 2***			
CRF5B	*25.1*	*MR Vaccine dose 2 Status (Check in the report and record the response)*	0|No<br>1|Yes	Check in the vaccination card and record the response	If response is "No", go to 26.<br>If response is "Yes", go to 25.2.
CRF5B	*25.2*	*MR Vaccine dose 2 Date of administration.*	NA	Click unknown if date is not known or illegible on the cards	Date should not be a future date.<br>Date should not be before DOB
CRF5B	***26***	***PCV dose 1***			
CRF5B	26.1	*PCV dose 1 Status*	0|No<br>1|Yes	Check in the vaccination card and record the response	If response is "No", go to 27.<br>If response is "Yes", go to 26.2.
CRF5B	*26.2*	*PCV dose 1 Date of administration.*	NA	Click unknown if date is not known or illegible on the cards	Date should not be a future date.<br>Date should not be before DOB
CRF5B	***27***	***PCV dose 2***			
CRF5B	*27.1*	*PCV dose 2 Status*	0|No<br>1|Yes	Check in the vaccination card and record the response	If response is "No", go to 28.<br>If response is "Yes", go to 27.2.
CRF5B	*27.2*	*PCV dose 2 Date of administration.*	NA	Click unknown if date is not known or illegible on the cards	Date should not be a future date.<br>Date should be later than PCV dose 1
CRF5B	***28***	***PCV booster dose***			
CRF5B	*28.1*	*PCV booster dose Status*	0|No<br>1|Yes	Check in the vaccination card and record the response	If response is "No", go to 29.<br>If response is "Yes", go to 28.2.
CRF5B	*28.2*	*PCV booster dose Date of administration.*	NA	Click unknown if date is not known or illegible on the cards	Date should not be a future date.<br>Date should be later than PCV dose 2
CRF5B	**29**	***Viral influenza vaccine (non-COVID)***			
CRF5B	*29.1*	*Viral influenza vaccine (non-COVID) Status*	0|No<br>1|Yes	Check in the vaccination card and record the response	If response is "Yes", go to 29.2.

CRF5B	*29.2*	*Viral influenza vaccine (non-COVID) Date of administration*	NA	Click unknown if date is not known or illegible on the cards	Date should not be a future date.<br>Date should not be before DOB
CRF5B		***Section 2-Immunisation history in individuals aged 5 years and above (only at baseline and endline)***		**Questions in Section 2 will be skipped if age is below 5. Form opens only at baseline and endline**	
CRF5B	*30*	*Has the participant received any vaccination in the past 1 year*	0|No<br>1|Yes	Ask the participant if he/she has taken any vaccination in the past one year and If "Yes", record the	If "No" skip all questions and go to Remarks.
CRF5B	31	*Pneumococcal pneumonia (PCV) vaccine*	0|No<br>1|Yes	Check in the vaccination card and record the response	If response is "No", go to 32.<br>If response is "Yes", go to 31.1.
CRF5B	*31.1*	*If Pneumococcal pneumonia (pcv) Vaccine Date of administration.*		Click unknown if date is not known or illegible on the cards	Date should not be a future date.<br>Date should not be before DOB
CRF5B	*31.2*	*Source of information for Pneumococcal pneumonia (pcv) Vaccine*	1|Vaccination card/ Prescription<br>2|Self report	The vaccinations would have been recorded on a health card given to the patient, which is the main source of information about vaccination. If the respondent having a vaccination card for the child, probe "May I see it?" Or check with medical records.<br>Check and record the response	If response is "Self report" skip next 31.3
CRF5B	*31.3*	*Upload picture of prescription if available for Pneumococcal pneumonia (pcv) Vaccine*	NA	click the image if vaccination card available	None
CRF5B	*32*	*Viral influenza (non-COVID) vaccine*	0|No<br>1|Yes	Check in the report and record the response	If response is "No", go to 33.<br>If response is "Yes", go to 32.1.
CRF5B	*32.1*	*Viral influenza (non-COVID) vaccine Date of administration.*		Click unknown if date is not known or illegible on the cards	Date should not be a future date.<br>Date should not be before DOB

CRF5B	*32.2*	*Source of information for Viral influenza (non-COVID) vaccine*	1|Vaccination card/ Prescription<br>2|Self report	The vaccinations would have been recorded on a health card given to the patient, which is the main source of information about vaccination. If the respondent having a vaccination card for the child, probe “May I see it?” Or check with medical records.<br>[illegible]	If response is "Self report", skip next 32.3
CRF5B	*32.3*	*Upload picture of prescription if available for Viral influenza (non- COVID) vaccine*	NA	click the image if vaccination card available	None
CRF5B	*33*	*COVID related influenza vaccine*	0|No<br>1|Yes	Check in the report and record the response	If response is "No”, go to 34.<br>If response is "Yes", go to 33.1.
CRF5B	*33.1*	*COVID related influenza vaccine Date of administration.*		Click unknown if date is not known or illegible on the cards	Date should not be a future date.<br>Date should not be before DOB
CRF5B	*33.2*	*Source of information for COVID related influenza vaccine*	1|Vaccination card/ Prescription<br>2|Self report	The vaccinations would have been recorded on a health card given to the patient, which is the main source of information about vaccination. If the respondent having a vaccination card for the child, probe “May I see it?” Or check with medical records.<br>Check and record the response	If response is "Self report", skip next 33.3
CRF5B	*33.3*	*Upload picture of prescription if available for COVID related influenza vaccine*	NA	click the image if vaccination card available	None
CRF5B	*34*	BCG vaccine	0|No<br>1|Yes	Check in the report and record the response	If response is "No", go to 35.<br>if response is "Yes", go to 34.1.

CRF5B	*34.1*	*BCG vaccine Date of administration.*		Click unknown if date is not known or illegible on the cards	Date should not be a future date. Date should not be before DOB
CRF5B	*34.2*	*Source of information for BCG vaccine*	1|Vaccination card/ Prescription<br>2|Self report	The vaccinations should be recorded on a health card given to the patient, which is the main source of information about vaccination. If the respondent reports having a card for the child, probe “May I see it?” Or check with medical records. Check and record the response	If response is "Self report", skip next 34.3
CRF5B	*34.3*	*Upload picture of prescription if available for BCG vaccine*	NA	click the image if vaccination card available	None
CRF5B	*35*	Measles vaccine	0|No<br>1|Yes	Check in the report and record the response	If response is "No", go to q36.<br>If response is "Yes", go to35.1.
CRF5B	*35.1*	*Measles vaccine Date of administration.*		Click unknown if date is not known or illegible on the cards	Date should not be a future date. Date should not be before DOB
CRF5B	*35.2*	*Source of information for Measles vaccine*	1|Vaccination card/ Prescription<br>2|Self report	The vaccinations should be recorded on a health card given to the patient, which is the main source of information about vaccination. If the respondent reports having a card for the child, probe “May I see it?” Or check with medical records. Check and record the response	If response is "Self report", skip next 35.3
CRF5B	*35.3*	*Upload picture of prescription if available for Measles vaccine*	NA	click the image if vaccination card available	None
CRF5B	*36*	Measles rubella vaccine	0|No<br>1|Yes	Check in the report and record the response	If response is "No", go to 37.<br>if response is "Yes", go to 36.1.
CRF5B	*36.1*	*Measles rubella vaccine Date of administration.*		Click unknown if date is not known or illegible on the cards	Date should not be a future date. Date should not be before DOB

CRF5B	*36.2*	*Source of information for Measles rubella vaccine*	1|Vaccination card/ Prescription<br>2|Self report	The vaccinations should be recorded on a health card given to the patient, which is the main source of Information about vaccination. If the respondent reports having a card for the child, probe “May I see it?” Or check with medical records. Check and record the response	f response is "Self report", skip next 36.3
CRF5B	*36.3*	*Upload picture of prescription if available for Measles rubella*	NA	click the image if vaccination card available	None
CRF5B	*37*	Dengue vaccine	0|No<br>1|Yes	Check in the report and record the response	If response is "No", go to 38.<br>if response is "Yes", go to 37.1
CRF5B	*37.1*	*Dengue vaccine Date of administration.*		Click unknown if date is not known or illegible on the cards	Date should not be a future date.<br>Date should not be before DOB
CRF5B	*37.2*	*Source of information for Dengue vaccine*	1|Vaccination card/ Prescription<br>2|Self report	The vaccinations should be recorded on a health card given to the patient, which is the main source of information about vaccination. If the respondent reports having a card for the child, probe “May I see it?” Or check with medical records. Check and record the response	If response is "Self report" skip next 37.3.
CRF5B	*37.3*	*Upload picture of prescription if available for Dengue Vaccine*	NA	click the image if vaccination card available	None
CRF5B	*38*	Hepatitis B Vaccine	0|No<br>1|Yes	Check in the report and record the response	If response is "No", go to 39.<br>If response is "Yes", go to 38.1.
CRF5B	*38.1*	*Hepatitis B vaccine Date of administration.*		Click unknown if date is not known or illegible on the cards	Date should not be a future date.<br>Date should not be before DOB

CRF5B	*38.2*	*Source of information for Hepatitis B vaccine*	1|Vaccination card/ Prescription<br>2|Self report	The vaccinations would have been recorded on a health card given to the patient, which is the main source of information about vaccination. If the respondent having a vaccination card for the child, probe “May I see it?” Or check with medical records.<br>Check and record the response	If option "Self report" skip 38.3
CRF5B	*38.3*	*Upload picture of prescription if available for Hepatitis B Vaccine*		click the image if vaccination card available	None
CRF5B	*39*	HPV (Human Papilloma virus) Vaccine	0|No<br>1|Yes	Check in the report and record the response	If response is "No", go to 40.<br>if response is "Yes", go to 39.1.
CRF5B	*39.1*	*HPV (Human Papilloma virus) Vaccine Date of administration.*		Click unknown if date is not known or illegible on the cards	Date should not be a future date.<br>Date should not be before DOB
CRF5B	*39.2*	*Source of information for HPV (Human Papilloma Virus) Vaccine*	1|Vaccination card/ Prescription<br>2|Self report	The vaccinations would have been recorded on a health card given to the patient, which is the main source of information about vaccination. If the respondent having a vaccination card for the child, probe “May I see it?” Or check with medical records.<br>Check and record the response	If response is "Self report" skip next 39.3
CRF5B	*39.3*	*Upload picture of prescription if available for HPV Vaccine*		click the image if vaccination card available	None
CRF5B	*40*	Tetanus toxoid Vaccine	0|No<br>1|Yes	Check in the report and record the response	If response is "No", go to 41.<br>If response is "Yes", go to 40.1.
CRF5B	*40.1*	*Tetanus toxoid Vaccine: Date of administration.*		Click unknown if date is not known or illegible on the cards	Date should not be a future date.<br>Date should not be before DOB

CRF5B	*40.2*	*Source of information for Tetanus toxoid Vaccine*	1|Vaccination card/ Prescription<br>2|Self report	The vaccinations would have been recorded on a health card given to the patient, which is the main source of information about vaccination. If the respondent having a vaccination card for the child, probe "May I see it?" Or check with medical records. Check and record the response	If response is "Self report", skip next 40.3.
CRF5B	*40.3*	*Upload picture of prescription if available for Tetanus toxoid*		click the image if vaccination card available	None
CRF5B	*41*	Anti-rabies Vaccine	0|No<br>1|Yes	Check in the report and record the response	If response is "No", go to 42.<br>if response is "Yes", go to 41.1.
CRF5B	*41.1*	*Anti-rabies Vaccine: Date of administration.*		Click unknown if date is not known or illegible on the cards	Date should not be a future date.<br>Date should not be before DOB
CRF5B	*41.2*	*Source of information for Anti rabies Vaccine*	1|Vaccination card/ Prescription<br>2|Self report	The vaccinations would have been recorded on a health card given to the patient, which is the main source of information about vaccination. If the respondent having a vaccination card for the child, probe "May I see it?" Or check with medical records.<br>Check and record the response	If response is "Self report" skip next 41.3
CRF5B	*41.3*	*Upload picture of prescription if available for Anti rabies Vaccine*		click the image if vaccination card available	None
CRF5B	*42*	JE Vaccine	0|No<br>1|Yes	Check in the report and record the response	if response is "No", go to 43.<br>if response is "Yes", go to 42.1.
CRF5B	*42.1*	*JE Vaccine: Date of administration.*		Click unknown if date is not known or illegible on the cards	Date should not be a future date.<br>Date should not be before DOB

CRF5B	*42.2*	*Source of information for JE Vaccine*	1|Vaccination card/ Prescription<br>2|Self report	The vaccinations would have been recorded on a health card given to the patient, which is the main source of information about vaccination. If the respondent having a vaccination card for the child, probe "May I see it?" Or check with medical records.<br>Check and record the response	If response is "Self report" skip next 42.3
CRF5B	*42.3*	*Upload picture of prescription if available for JE Vaccine*		click the image if vaccination card available	None
CRF5B	*43*	*Others: Apart from the Vaccination mentioned above, has the participant been taking any others*	0|No<br>1|Yes	Check in the report and record the response	If response is "No" go to 44 Remarks<br>If response is "Yes", go to 43.1
CRF5B	*43.1*	*If "Yes", mention the name of the vaccine*		Ask the name of the vaccine	None
CRF5B	*43.2*	*Date of administration. Enter unknown if date is not known or illegible on the card*		Click unknown if date is not known or illegible on the cards	Date should not be a future date.<br>Date should not be before DOB
CRF5B	*43.3*	*Source of information*	1|Vaccination card/ Prescription<br>2|Self report	The vaccinations would have been recorded on a health card, which is the main source of information about vaccination.<br>Check and record the response	If response is "Self report" skip next 43.4
CRF5B	*43.4*	*Upload picture of prescription if available*		click the image if vaccination card available	
CRF5B	*44*	*Remarks for immunization History*			None

Form	Q No	Questions	Response options	Instruction to Data collector	Built in software logics
**Serosurvey History and Examination**					
CRF 5C	1	*Was the participant pregnant at the time of contact (applicable to females of 15-45 age group)*	0|No<br>1|Yes<br>2|Unknown	Ask and record the appropriate response	This question will be skipped for males and unmarried individuals and those above 50 years.<br>If response is "No or Unknown", skip 1.1<br>If response is "Yes" skip 1.2
CRF 5C	*1.1*	*If "Yes", which Trimester is she currently in?*	1|1st Trimester<br>2|2nd Trimester<br>3|3rd Trimester	Ask and record the appropriate response	This question will be skipped for males and unmarried individuals and those above 50 years
CRF 5C	*1.2*	*Was the participant in the post-partum period or lactation period (6 months post-delivery) at the time of contact (applicable to females of 15-45 age group)*	0|No<br>1|Yes<br>2|Unknown<br>8|Not Applicable		This question will be skipped for males and unmarried individuals and those above 50 years
CRF 5C	*2*	*Is the participant a known case of diabetes mellitus?*	0|No<br>1|Yes<br>2|Unknown	This question serves to identify individuals with a diagnosed case of diabetes or diabetes mellitus (sometimes called "high blood sugar"). Ask and record appropriate response	If response is "No or Unknown", skip 2.1 and 2.2<br>If response is "Yes", go to 2.1

CRF 5C	*2.1*	*Diabetes Mellitus: Is there any evidence to support the presence or absence of this condition?*	1| Medical record with diagnosis available<br>2| Medical prescription available<br>3|Confirmatory lab report available<br>4|Medications available with participant<br>5|No proof available	Ask the participant for any available medical records	If response is "No proof available", skip 2.2
CRF 5C	2.2	*For Diabetes Mellitus: Upload a picture of documentary proof to support the diagnosis.*	NA	Ask the participant for Doctor's report with diagnosis, prescription for medications for this condition or any confirmatory lab test. In case none of these documents are available but the participant shows medications for this condition, please upload picture of the medication with name of the drug visible. In case the same document serves as proof for multiple conditions, please upload the same image against each condition.	None
CRF 5C	*3*	*Is the participant a known case of hypertension?*	0|No<br>1|Yes<br>2|Unknown	This question serves to identify individuals with a diagnosed case of hypertension or (sometimes called "high pressure"). Ask and record appropriate response,	If response is "No or Unknown", skip 3.1 and 3.2<br>If response is "Yes", go to 3.1

CRF 5C	*3.1*	*Hypertension: Is there any evidence to support the presence or absence of this condition?*	1| Medical record with diagnosis available<br>2 |Medical prescription available<br>3|Confirmatory lab report available<br>4|Medications available with participant<br>5|No proof available	Ask the participant for any available medical records	If response is "No proof available" skip 3.2
CRF 5C	*3.2*	*For Hypertension: Upload a picture of documentary proof to support the diagnosis.*	NA	Ask the participant for Doctor's report with diagnosis, prescription for medications for this condition or any confirmatory lab test. In case none of these documents are available but the participant shows medications for this condition, please upload picture of the medication with name of the drug visible. In case the same document serves as proof for multiple conditions, please upload the same image against each condition.	None
CRF 5C	*4*	*Is the participant a known case of cardio vascular Disease (eg. Previous Heart attack, Valvular abnormalities, rhythm abnormalities or irregularities)*	0|No<br>1|Yes<br>2|Unknown		If response is "No or Unknown", skip 4.1 and 4.2<br>If response is "Yes", go to 4.1

CRF 5C	4.1	*Cardiovascular Disease: Is there any evidence to support the presence or absence of this condition?*	1| Medical record with diagnosis available<br>2| Medical prescription available<br>3|Confirmatory lab report available<br>4|Medications available with participant<br>5|No proof available	Ask the participant for any available medical records	If response is "No proof available", skip 4.2
CRF 5C	*4.2*	*For Cardiovascular diseases: Upload a picture of documentary proof to support the diagnosis.*	NA	Ask the participant for Doctor's report with diagnosis, prescription for medications for this condition or any confirmatory lab test. In case none of these documents are available but the participant shows medications for this condition, please upload picture of the medication with name of the drug visible. In case the same document serves as proof for multiple conditions, please upload the same image against each condition.	None
CRF 5C	*5*	*Is the participant a known case of Chronic Liver Disease (Jaundice, Hepatitis B/C infection, Cirrhosis, accumulation of water in the abdomen, damaged liver for more than 6 months)<br>(Ask and record appropriate response, If necessary, probe)*	0|No<br>1|Yes<br>2|Unknown		If response is "No or Unknown", skip 5.1 and 5.2<br>If response is "Yes", go to 5.1

CRF 5C	*5.1*	*Chronic Liver Disease: Is there any evidence to support the presence or absence of this condition?*<br>*(Ask the participant for any available medical records)*	1| Medical record with diagnosis available<br>2 |Medical prescription available<br>3|Confirmatory lab report available<br>4|Medications available with participant<br>5|No proof available	Ask the participant for any available medical records	If response is "No proof available", skip 5.2
CRF 5C	*5.2*	*For Chronic Liver Disease: Upload a picture of documentary proof to support the diagnosis. (Doctor's report with diagnosis, prescription for medications for this condition or any confirmatory lab test). In case none of these documents are available but the participant shows medications for this condition, please upload picture of the medication with name of the drug visible. In case the same document serves as proof for multiple conditions, please upload the same image against each condition.*	NA	Ask the participant for Doctor's report with diagnosis, prescription for medications for this condition or any confirmatory lab test. In case none of these documents are available but the participant shows medications for this condition, please upload picture of the medication with name of the drug visible. In case the same document serves as proof for multiple conditions, please upload the same image against each condition.	None

CRF 5C	*6*	*Is the participant a known case of Chronic Renal / Kidney Disease (blood in urine, kidney failure, need for dialysis)*<br>*(Ask and record appropriate response, If necessary, probe)*	0|No<br>1|Yes<br>2|Unknown		If response is "No or Unknown", skip 6.1 and 6.2.<br>If response is "Yes", go to 6.1
CRF 5C	*6.1*	*Chronic Renal / Kidney Disease: Is there any evidence to support the presence or absence of this condition?*<br>*(Ask the participant for any available medical records)*	1| Medical record with diagnosis available<br>2 |Medical prescription available<br>3|Confirmatory lab report available<br>4|Medications available with participant<br>5|No proof available	Ask the participant for any available medical records	If response is "No proof available" skip 6.2

CRF 5C	*6.2*	*For Chronic Renal / Kidney Disease: Upload a picture of documentary proof to support the diagnosis. (Doctor's report with diagnosis, prescription for medications for this condition or any confirmatory lab test). In case none of these documents are available but the participant shows medications for this condition, please upload picture of the medication with name of the drug visible. In case the same document serves as proof for multiple conditions, please upload the same image against each condition.*	NA	Ask the participant for Doctor's report with diagnosis, prescription for medications for this condition or any confirmatory lab test. In case none of these documents are available but the participant shows medications for this condition, please upload picture of the medication with name of the drug visible. In case the same document serves as proof for multiple conditions, please upload the same image against each condition.	None
CRF 5C	*7*	*Is the participant a known case of Chronic Neurological / Neuromuscular Disease (Dementia, loss of memory, recognition, brain haemmorhage, paralysis, inability to walk, seizures, paresthesia / numbness of the limbs)*<br>*(Ask and record appropriate response, If necessary, probe)*	0|No<br>1|Yes<br>2|Unknown		If response is "No or Unknown", skip 7.1 and 7.2<br>If response is "Yes", go to 7.1

CRF 5C	*7.1*	*Chronic Neurological / Neuromuscular Disease: Is there any evidence to support the presence or absence of this condition?*<br>*(Ask the participant for any available medical records)*	1| Medical record with diagnosis available<br>2 |Medical prescription available<br>3|Confirmatory lab report available<br>4|Medications available with participant<br>5|No proof available	Ask the participant for any available medical records	If response is "No proof available" skip 7.2
CRF 5C	*7.2*	*For Chronic Neurological / Neuromuscular Disease: Upload a picture of documentary proof to support the diagnosis. (Doctor's report with diagnosis, prescription for medications for this condition or any confirmatory lab test). In case none of these documents are available but the participant shows medications for this condition, please upload picture of the medication with name of the drug visible. In case the same document serves as proof for multiple conditions, please upload the same image against each condition.*	NA	Ask the participant for Doctor's report with diagnosis, prescription for medications for this condition or any confirmatory lab test. In case none of these documents are available but the participant shows medications for this condition, please upload picture of the medication with name of the drug visible. In case the same document serves as proof for multiple conditions, please upload the same image against each condition.	None

CRF 5C	*8*	*Is the participant a known case of Chronic Lung Disease? (Asthma, Bronchitis, chronic cough, breathing difficulty) (Ask and record appropriate response, If necessary, probe)*	0|No<br>1|Yes<br>2|Unknown		If response is "No or Unknown", skip 8.1 and 8.2<br>If response is "Yes", go to 8.1
CRF 5C	*8.1*	*Chronic Lung Disease: Is there any evidence to support the presence or absence of this condition? (Ask the participant for any available medical records)*	1| Medical record with diagnosis available<br>2 |Medical prescription available<br>3|Confirmatory lab report available<br>4|Medications available with participant<br>5|No proof available	Ask the participant for any available medical records	If response is "No proof available" skip 8.2

CRF 5C	*8.2*	*Chronic Lung Disease: Upload a picture of documentary proof to support the diagnosis. (Doctor's report with diagnosis, prescription for medications for this condition or any confirmatory lab test). In case none of these documents are available but the participant shows medications for this condition, please upload picture of the medication with name of the drug visible. In case the same document serves as proof for multiple conditions, please upload the same image against each condition.*	NA	Ask the participant for Doctor's report with diagnosis, prescription for medications for this condition or any confirmatory lab test. In case none of these documents are available but the participant shows medications for this condition, please upload picture of the medication with name of the drug visible. In case the same document serves as proof for multiple conditions, please upload the same image against each condition.	None
CRF 5C	*9*	*Is the participant at risk of being in an immunosuppressive state (treatment for autoimmune disorders like SLE, Rheumatoid Arthritis, Malignancy, Bone marrow recipient, Organ Transplant etc.)*<br>*(Ask and record appropriate response, If necessary, probe)*	0|No<br>1|Yes<br>2|Unknown		If response is "No or Unknown", skip 9.1 and 9.2<br>If response is "Yes", go to q9.1

CRF 5C	*9.1*	*immunosuppressive state: Is there any evidence to support the presence or absence of this condition?*<br>*(Ask the participant for any available medical records)*	1| Medical record with diagnosis available<br>2 |Medical prescription available<br>3|Confirmatory lab report available<br>4|Medications available with participant.<br>5|No proof available	Ask the participant for any available medical records	If response is "No proof available" skip 9.2
CRF 5C	*9.2*	*For immunosuppressive state: Upload a picture of documentary proof to support the diagnosis. (Doctor's report with diagnosis, prescription for medications for this condition or any confirmatory lab test). In case none of these documents are available but the participant shows medications for this condition, please upload picture of the medication with name of the drug visible. In case the same document serves as proof for multiple conditions, please upload the same image against each condition.*	NA	Ask the participant for Doctor's report with diagnosis, prescription for medications for this condition or any confirmatory lab test. In case none of these documents are available but the participant shows medications for this condition, please upload picture of the medication with name of the drug visible. In case the same document serves as proof for multiple conditions, please upload the same image against each condition.	None

CRF 5C	*10*	*Is the participant known to have HIV infection?*<br>*(Ask and record appropriate response, If necessary, probe)*	0|No<br>1|Yes<br>2|Unknown		If response is "No or Unknown", skip 10.1 and 10.2<br>if response is "Yes", go to 10.1
CRF 5C	*10.1*	*HIV infection: Is there any evidence to support the presence or absence of this condition?*<br>*(Ask the participant for any available medical records)*	1| Medical record with diagnosis available<br>2 |Medical prescription available<br>3|Confirmatory lab report available<br>4|Medications available with participant<br>5|No proof available	Ask the participant for any available medical records	If response is "No proof available" skip 10.2

CRF 5C	*10.2*	*For HIV infection: Upload a picture of documentary proof to support the diagnosis. (Doctor's report with diagnosis, prescription for medications for this condition or any confirmatory lab test). In case none of these documents are available but the participant shows medications for this condition, please upload picture of the medication with name of the drug visible. In case the same document serves as proof for multiple conditions, please upload the same image against each condition.*	NA	Ask the participant for Doctor's report with diagnosis, prescription for medications for this condition or any confirmatory lab test. In case none of these documents are available but the participant shows medications for this condition, please upload picture of the medication with name of the drug visible. In case the same document serves as proof for multiple conditions, please upload the same image against each condition.	None
CRF 5C	*11*	*Is the participant known to be suffering from tuberculosis? (Ask and record appropriate response, If necessary, probe)*	0|No<br>1|Yes<br>2|Unknown		If response is "No or Unknown", skip 11.1 and 11.2<br>If response is "Yes", go to 11.1
CRF 5C	*11.1*	*Tuberculosis, is there any evidence to support the presence or absence of this condition?*<br>*(Ask the participant for any available medical records)*	1| Medical record with diagnosis available<br>2 |Medical prescription available<br>3|Confirmatory lab report available<br>4|Medications available with participant<br>5|No proof available	Ask the participant for any available medical records	If response is "No proof available", skip 11.2

CRF 5C	*11.2*	*For Tuberculosis: Upload a picture of documentary proof to support the diagnosis. (Doctor's report with diagnosis, prescription for medications for this condition or any confirmatory lab test). In case none of these documents are available but the participant shows medications for this condition, please upload picture of the medication with name of the drug visible. In case the same document serves as proof for multiple conditions, please upload the same image against each condition.*	NA	Ask the participant for Doctor's report with diagnosis, prescription for medications for this condition or any confirmatory lab test. In case none of these documents are available but the participant shows medications for this condition, please upload picture of the medication with name of the drug visible. In case the same document serves as proof for multiple conditions, please upload the same image against each condition.	None
CRF 5C	*12*	*Other disease: Apart from the conditions mentioned above, has the participant been taking any medications for more than three months prior to the day of interview?*<br>*(Ask and record appropriate response, If necessary, probe)*	0|No<br>1|Yes<br>2|Unknown		If response is "No or Unknown", skip 12.1, 12.2 and 12.3<br>If response is "Yes", go to 12.1
CRF 5C	*12.1*	*Other disease: Mention the diagnosis for which the medications are being taken (Ask for medical records to see the diagnosis, if any / ask the participant /care giver regarding it and record the response as available)*	NA	Ask the participant to mention the diagnosis for which the medications are being taken.	

CRF 5C	*12.2*	*Other disease: Is there any evidence to support the presence or absence of this condition?<br>(Ask the participant for any available medical records)*	1| Medical record with diagnosis available<br>2 |Medical prescription available<br>3|Confirmatory lab report available<br>4|Medications available with participant<br>5|No proof available	Ask the participant for any available medical records	If response is "No proof available" skip 12.3
CRF 5C	*12.3*	*For Other disease: Upload a picture of documentary proof to support the diagnosis. (Doctor's report with diagnosis, prescription for medications for this condition or any confirmatory lab test). In case none of these documents are available but the participant shows medications for this condition, please upload picture of the medication with name of the drug visible. In case the same document serves as proof for multiple conditions, please upload the same image against each condition.*	NA	Ask the participant for Doctor's report with diagnosis, prescription for medications for this condition or any confirmatory lab test. In case none of these documents are available but the participant shows medications for this condition, please upload picture of the medication with name of the drug visible. In case the same document serves as proof for multiple conditions, please upload the same image against each condition.	None

Section 2-Examination					
CRF 5C	*13*	*Was examination complete?*	0|No<br>1|Yes	Field worker have to record the vital measurements of participant includes weight, height, BP, temperature etc.	If response is "No", go to 20 Remarks.<br>If response is "Yes", go to 14
CRF 5C	*14*	*Record the temperature of participant using infrared thermometer*	NA	Record the temperature of participant, if temperature not measured, click unknown option	None
CRF 5C	*15.1*	*Systolic Blood Pressure (Request participant to be seated calmly for at least 5 minutes in a chair, with feet on the floor, and arm supported at heart level. Take two readings 5 minutes apart)*	NA	Record the blood pressure of participant, if t not measured, click unknown option	If the participant is <18yr, this question will be skipped
CRF 5C	*15.2*	*Diastolic Blood Pressure*	NA		If the participant is <18yr, this question will be skipped
CRF 5C	*15.3*	*Systolic Blood Pressure -2nd reading (Request participant to be seated calmly for at least 5 minutes in a chair, with feet on the floor, and arm supported at heart level.)*	NA	Record the blood pressure of participant, if not measured, click unknown option	If the participant is <18yr, this question will be skipped
CRF 5C	*15.4*	*Diastolic Blood Pressure*	NA		if the participant is <18yr, this question will be skipped
CRF 5C	*16*	*SpO2 (Record after placing the pulse-oximeter in the finger for one minute and a steady waveform is visible on the display panel of the pulse oximeter)*	NA	Record the spo2 of participant, if not measured, click unknown option	None

CRF 5C	*17*	*Height. Request participant to take off their shoes, put their feet and heels close together, stand straight and look straight ahead standing with their back, head and heels touching the vertical column of the height rod.*	NA	Record the height of participant, if not measured, click unknown option	None
CRF 5C	*18*	*Weight. Request participant to take their shoes off and step on the scale.*	NA	Record the weight of participant, if not measured, click unknown option	None
CRF 5C	*19*	*Mid Upper Arm Circumference*	NA	Record the Mid upper arm circumference of participant, if not measured, click unknown option	None
CRF 5C	*20*	*Remarks for History and Examination*	NA		None

Form	Q No	Questions	Response options	Instruction to Data collector	Built in software logics
**Sample Collection in Serum Separator Tube**					
CRF5D.1	*1*	*Did the participant agree to provide blood sample?*	1|Agreed<br>2|Refused	Ask the permission of the participant to withdraw blood sample	If Response is "Refused" go to 3<br>If response is "Agreed", go to 2
CRF5D.1	*2*	*Was phlebotomist / nurse able to collect the blood sample in the serum separator tube (SST)?*	0|No<br>1|Yes		If Response "No", go to 2.1<br>If response "Yes" go to 2.2
CRF5D.1	2.1	*If No, state reason*	1|Unable to access vein<br>2|Volume insufficient<br>3|Not cooperative		None
CRF5D.1	2.2	*Volume of sample collected in SST*		Enter the volume of sample collected in SST tube	Range 1 to 6ml
CRF5D.1	2.3	*Sample ID collected in SST (Scan the barcode on the tube)*	NA	Exactly 10 characters long.<br>Last two characters should be "SS".	None
CRF5D.1	2.4	*Date and Time of collection of blood sample*	NA		Date cannot be future
CRF5D.1	3	*Any Remarks*		NA	None

Form	Q No	Questions	Response options	Instruction to Data collector	Built in software logics
**Sample Collection in EDTA tube**					
CRF 5D.2					This form will be skipped for less than 18 years of age
CRF5D.2	1	*Was phlebotomist able to collect the blood sample in the EDTA tube?*	0|No<br>1|Yes		If Response is "No", skip 1.2 and 1.3.<br>If response is "Yes" skip 1.1"
CRF5D.2	1.1	*If No, state reason*	1|Unable to access vein<br>2|Volume insufficient<br>3|Not cooperative		None
CRF5D.2	1.2	*Volume of Sample collected in EDTA tube*	NA	Enter the volume of sample collected in SST tube	Range 0 to 5ml
CRF5D.2	1.3	*Date and Time of collection of blood sample*	NA		Date cannot be future
CRF5D.2	1.4	*Sample ID collected in EDTA tube (in baseline round for HbA1c)- (Scan the barcode on the tube)*	NA	Exactly 10 characters long. Last two characters should be "ED"	None
CRF5D.2	2	*Any Remarks*			None

Form	Q:o	*Questions*	Response options	Instruction to Data collector	Built in software logics
**Point of Care Tests**					
CRF5D.3	1	*Was on spot Hemoglobin level measured?*	0|No<br>1|Yes		If response is "No" skip 1.1.<br>If response is "Yes", go to 1.1
CRF5D.3	1.1	*If "Yes", record the hemoglobin Level*		If the value is below 10% ask the participant to seek medical consultation	Range 1 to 20
CRF5D.3	2	*Was on spot blood glucose measured?*	0|No<br>1|Yes	If the value is below 70mg/dl or above 200 mg/dl, ask the participant to seek medical consultation	This question will be skipped for <18year of age
CRF5D.3	2.1	*If "Yes", record the RBS value*	NA		This question will be skipped for <18year of age.<br>Range:20 to 500
