# Supplementary material for: Sero-Surveillance to Monitor the Trend of SARS-CoV-2 Infection Transmission in India: Study Protocol for a Multi Site, Community Based Longitudinal Cohort Study
Source: Front Public Health. 2022 Mar 24;10:810353. doi: 10.3389/fpubh.2022.810353 (PMC8987192; doi:10.3389/fpubh.2022.810353)
Supplement: Supplementary file 3 [file Data_Sheet_3.PDF]

Establish serial sero-surveillance to monitor the trend of SARS-CoV-2, Dengue and Chikungunya infection transmission in the general population, India

**STUDY TITLE:**	**DRIVEN 2020 STUDY**
**STUDY IDENTIFIER:**	**DRIVEN/2020/01**
**DOCUMENT TITLE:**	**BLOOD SAMPLE MANAGEMENT AND TESTING MANUAL (**Blood sample collection, labeling, storage)
**DOCUMENT VERSION:**	**VERSION - 05; DATED – 8 December 2020**
**FUNDING AGENCY**	**NATIONAL BIOPHARMA MISSION (NBM)**<br><br>Biotechnology Industry Research Assistance Council (BIRAC)<br><br>1st Floor, MTNL Building ,9 CGO Complex, Lodhi Road, New Delhi-110003

**Prepared by:**		
Dr Shikha T Malik	Program Manager	BIRAC-National Biopharma Mission
Dr Rahul Shrivastava	Sr Program Officer	BIRAC-National Biopharma Mission
Dr Reshma Raju	Sr Research Officer	Christian Medical College, Vellore
**Reviewed by:**		
Dr Kavita Singh	Mission Director	BIRAC- National Biopharma Mission
Dr Akhilesh Chandra Mishra	Director	IRSHA, Pune
Dr Vidya Arankalle	Senior Scientist	IRSHA, Pune
Dr Sonali Sanghavi	Senior Microbiologist	KEMHRC, Pune
Ms Meenakshi Bakshi	Consultant - Quality Assurance	CDSA, Faridabad
**Approved by:**		
Dr Yuvaraj Jayaraman	Scientist G Medical	ICMR-NIE, Chennai
Dr Winsley Rose	Professor	CMC, Vellore
Dr Guru Rajesh Jammy	Associate Research Director	SHARE INDIA, Medchal
Dr Sanjay Juvekar	Officer In Charge	KEMHRC (VRHP), Vadu
Dr Shikha Dixit	Sr Program Officer	INCLEN, New Delhi

List of Sites and Details

**Site Details**		
**Sr No.**	**Site Name**	**Postal Address**
1	Society for Health Allied Research & Education India	Mediciti Institute of Medical Sciences campus, Ghanpur Village, Medchal Mandal, Telangana PIN-501401
2	Christian Medical College Vellore Association	Krishna Nagar Office, Door No: 9 and 9/1, Natteri Krishnamachari Street, Krishna Nagar, Vellore, Tamil Nadu 632004
3	ICMR – National Institute of Epidemiology	Model Rural Health Research Unit, Government Primary Health Centre Nadu Kallur Village, Chennai, Tamil Nadu - 600077
4	The INCLEN International Trust	SOMAARTH DDESS, H. Khata No,1460/1582, Village – Mitrol, NH 19, Aurangabad, Delhi Agra Highway, Palwal, Haryana- 121105
5	KEM Hospital Research Centre	Vadu Rural Health Program, Vadu HDSS, KEM Hospital Research Centre Pune, At Post Vadu (Budruk), Taluka Shirur District, Pune 421126
**Central Laboratory(s)**		
1	Interactive Research School of Health Affairs (IRSHA)	Pune-Satara Road, Katraj Dhankawadi, Pune, MAHARASHTRA, India - 411043
2	Translational Health Science and Technology Institute (THSTI)	3rd Milestone, Faridabad-Gurgaon Expressway, PO box no #04, Faridabad- Haryana 121001
**NABL Laboratory Details**		
1	Dr Dang's Laboratory	Aurobindo Market, C2/1, S.D.A. Aurobindo Marg, New Delhi, Delhi 110016
2	Christian Medical College Vellore Association	IgG ELISA: Dept. of Clinical Virology, HbA1C: Dept. of Clinical Biochemistry, Krishna Nagar Office, Door No: 9 and 9/1, Natteri Krishnamachari Street, Krishna Nagar, Vellore, Tamil Nadu 632004
**Bio-repository**		
1	THSTI	NCR Biotech Science Cluster 3rd Milestone, Faridabad - Gurgaon Rd, Expressway, Faridabad, Haryana 121001

Contact detail for Data Management Query:

**Data Management Platform**	SOMAARTH-3 (The INCLEN Trust International, New Delhi)

**Data Management related query**	Mr Neeraj Kashyap<br>Sr. Program Officer<br>neeraj.kashyap@inclentrust.org<br>8860355718	The Inclen Trust International<br>F-1/5, Okhla Industrial Area<br>Phase - 1, New Delhi, Delhi<br>110020

List of Abbreviations

BIRAC	Biotechnology Industry Research Assistance Council
BMW	Bio Medical Waste
CDSA	Clinical Development Services Agency
CMC	Christian Medical College
CRF	Case Report Form
EDC	Electronic Data Collection
ELISA	Enzyme-Linked Immunoassay
Hb	Hemoglobin
HbA1c	Glycated Hemoglobin
ICMR	Indian Council of Medical Research
IgG	Immunoglobulin G
ILI	Influenza Like Illness
IRSHA	Interactive Research School for Health Affairs
KEMHRC	King Edward Memorial Hospital Research Centre
NBM	National Biopharma Mission
NIE	National Institute of Epidemiology
PID	Participant Identification Number
POC	Point of Care
PPE	Personal Protective Equipment
PRNT	Plaque Reduction Neutralization Test
RPM	Revolutions Per Minute
SARI	Severe Acute Respiratory Infections
SARS-CoV2	Severe Acute Respiratory Syndrome Coronavirus 2
SHARE	Society for Health Allied Research Education
THSTI	Translational Health Science and Technology Institute
WHO	World Health Organization

Lab Expert Committee

Name	Designation	Organization
Dr Vidya A Arankalle	Senior Scientist	IRSHA, Pune
Dr Akhilesh Chandra Mishra	Director	IRSHA, Pune
Ms Meenakshi Bakshi	Consultant- Quality Assurance	CDSA, Faridabad
Dr Sonali Sanghavi	Senior Microbiologist	KEMHRC, Pune
Dr Divya Nair	Program Officer	INCLEN, New Delhi
Dr Sucheta Kurundkar	Director Training	THSTI, Faridabad
Dr Pallavi Kshetrapal	Assistant Professor	THSTI, Faridabad
Dr Guruprasad Medigeshi	Professor	THSTI, Faridabad

AMENDMENT RECORD:

Version	Page no.	Revision date	Author (Name, title, affiliations)	Location in Document and Description of Change	Reason for Change

TABLE OF CONTENTS:

1 Introduction ... 13

2 Scope ... 13

3 Biosafety ... 13

4 Serial Sero-Surveys ... 14

5 Overview: Flow Chart, Testing Plan and Volume of Samples Required ... 15

5.1 Key Notes for the Sample Flow: ... 16

5.2 Details Of Investigations/Assays Planned: ... 17

6 Sample Management Through Life Cycle ... 20

6.1 Preparation of sample collection kits ... 17

6.2 Sample Collectiona and Reporting in Field (Home) ... 22

6.2.1 Volume Collection ... 22

6.2.2 Labelling ... 22

6.2.3 Testing and Reporting in Field (Home) ... 23

6.3 Sample Management at Field Office ... 23

6.3.1 Sample Transfer from Field and Receipt at Field Office ... 23

6.3.2 Separation ... 24

6.3.3 Aliquots ... 25

6.3.4 Storage at Field Office Lab ... 27

6.3.5 Cryo-Box Label ... 28

6.3.6 Packaging for Shipment ... 29

6.3.7 Shipment Receipt at Dr Dangs Lab ... 29

6.3.8 Sample Receipt At Thsti Biorepository ... 29

7 Assays for Covid, Dengue and Chikungunya ... 30

7.1 For SARS-COV-2 IgG ... 30

7.2 For Dengue ... 31

7.3 For Chikungunya ... 31

8 General Guidelines For Good Laboratory Practices ... 32

8.1 Sample Collection ... 32

8.1.1 Preparing the Participant: ... 33

8.1.2 Inoculation of Blood into the Haemacue ... 36

8.1.3 Inoculation of Blood into the Glucometer ... 37

9 Bio-Medical Waste Disposal ... 37

10 Standard Precautions For Covid-19 Infection Prevention and Control ... 38

11 Documentation ... 39

12 Logistics 39
13 Reporting of the Test Results 40
14 Quality Control 41
15 Post-Exposure Prophylaxis for Needle Stick Injuries 41
16 Management of Blood Spill 43

LIST OF ANNEXURES:

**Sr. No.**	**Annexure No.**	**Particular**
1	01	Barcoding labelling scheme for Primary tubes. Cryo-vials and Sample Storage boxes

LIST OF FIGURES:

**Particulars**	**Page no.**
Figure 1: Overview: Flow Chart, testing plan and volume of samples required	14
Figure 2: Pictorial representation of Sample Flow in Round 1 and Round 4	14
Figure 3: Pictorial representation of Sample Flow in Round 2 and Round 3	15
Figure 4: Barcode Labelling Scheme	22
Figure 5: Barcode placement on primary tube	22
Figure 6: Vaccine carrier box	23
Figure 7: Cardboard box with a layout	25
Figure 8: Investigation flow for COVID assay	31
Figure 9: Illustration for IgG ELISA for Dengue assays	32
Figure 10: Illustration for IgG ELISA for Chikungunya assays	32
Figure 11: Identification vein to puncture for blood withdrawal	34
Figure 12: Sample collection and storage	35
Figure 13: Steps for blood collection	36

LIST OF TABLES:

**Particulars**	**Page no.**
Table 1: Laboratory assays to be performed in serial serosurvey rounds (All Age Groups)	18
Table 2: Biochemical investigations in serial serosurvey rounds	18
Table 3: Blood volumes required for <18 years age group	19
Table 4: Blood volumes required for ≥18 years age group	19
Table 5: Volume of blood sample required for performing tests in serial serosurvey rounds	22
Table 6: Aliquots and shipment >=18 years age group	27
Table 7: Aliquots and shipment <18 years age group	27
Table 8: List of Consumables	40

1 INTRODUCTION

DRIVEN 2020 study is aimed at measuring the antibodies in population cohorts against 3 diseases (COVID-19, Dengue and Chikungunya) through serial serosurveys.

This community-based serosurvey is being conducted at the following 5 sites across India:

1. Christian Medical College, Vellore, Tamil Nadu
2. ICMR-National Institute of Epidemiology- Model Rural Health Research Unit, Tirunelveli, Tamil Nadu
3. Society for Health Allied Research Education India (SHARE INDIA), Medchal, Telangana
4. SOMAARTH-DDESS, The INCLEN Trust International, Palwal, Haryana
5. Vadu HDSS, KEM Hospital Research Centre (KEMHRC), Pune, Maharashtra

Note: 1-5 indicate site code for the above-mentioned enrolling sites in the study.

2 SCOPE

This lab manual is based on ethics approved **Study Protocol version 5 dated 8 December 2020** and describes the applicable procedures for:

- Collection and labelling of blood samples from the participants
- Sample transport to field office
- Processing, labelling and aliquoting at field office
- Samples storage at field office unless transfer to designated labs/locations
- Sample packaging and shipment/pick up from field office to designation lab locations under appropriate conditions
- Sample receipt at respective lab locations
- Biomedical waste (BMW) segregation and disposal
- Ensure adequate protection of field and lab workers
- Related documentation standards and logs/data capture

Note: In addition to the process described in the manual, each site may adopt their best practices and specific technical processes including quality control tools to ensure study objectives are met.

3 BIOSAFETY

Safety of the participants and the workers is a priority and hence all efforts shall be made by the sites to ensure safe handling of samples to prevent any possible infection due to blood borne pathogens. In this respect:

- Adequate training of the staff on biosafety and Personal Protective Equipment (PPE) shall be imparted throughout the sample collection and transportation so as to ensure compliance.

- The field staff must ensure the safe and responsible disposal of all biomedical waste (BMW). generated through segregation at the point of origin for transfer and disposal at site through authorised service provider.
- Sites shall be responsible to ensure statutory compliance with respect to state specific BMW requirements. Site shall keep a record of BMW as per the norms.

Refer sections 9, 15 and 16 for more information on safety aspects and BMW.

4 SERIAL SERO-SURVEYS

- Administer informed consent process as per ICH-GCP guidelines and as per the ethics approved study protocol. Informed consent document (ICD) comprises of subject information sheet (SIS) and Informed Consent Form (ICF). Obtain written informed consent/assent from the willing participants. Only those individuals who consent shall be eligible for participation.
- A total of 5000 participants are planned to be enrolled at each site. Age eligibility is defined in study protocol (≥ 2 years). All participants enrolled in the study require a blood sample draw at the time of enrolment 0 months (Round -1), 4 months (Round -2), 8 months (Round -3) and 12 months (Round -4). A baseline round of serosurvey i.e. Round-1 will be conducted in all asymptomatic participants within the first two months of enrolment. Age group specific tests are defined in section: Serum samples collected from each participant across four sero-surveys will be tested for IgG antibodies against SARS-CoV-2 infection using lab-based CMIA/ELISA tests.
- Samples will also be tested for IgG antibodies against Dengue and Chikungunya infection by ELISA methodology. In Round 1 & Round 4 of serosurvey for a select subset of participants bases on selection rationale Samples shall be shipped to respective testing laboratory as per the defined plan (refer section 5 for the sample management flow).
- Enrolled participants of the cohort in this study, after completing the baseline serosurvey, will be followed for three more sequential rounds of sero-surveys at 4, 8 and 12 months respectively. The site team shall follow up enrolled participants as per established procedures.
- If the participant refuses to give a blood sample after signing the consent, consider this as:

- **Consent withdrawal:** if it is the first round (R1),
- **Loss to follow up**: if it is any of the subsequent rounds (R2/R3/R4).

The decision on tests to be done in subsequent rounds will be based on the results of the previous round of serosurvey.

5 Overview: Flow Chart, testing plan and volume of samples required

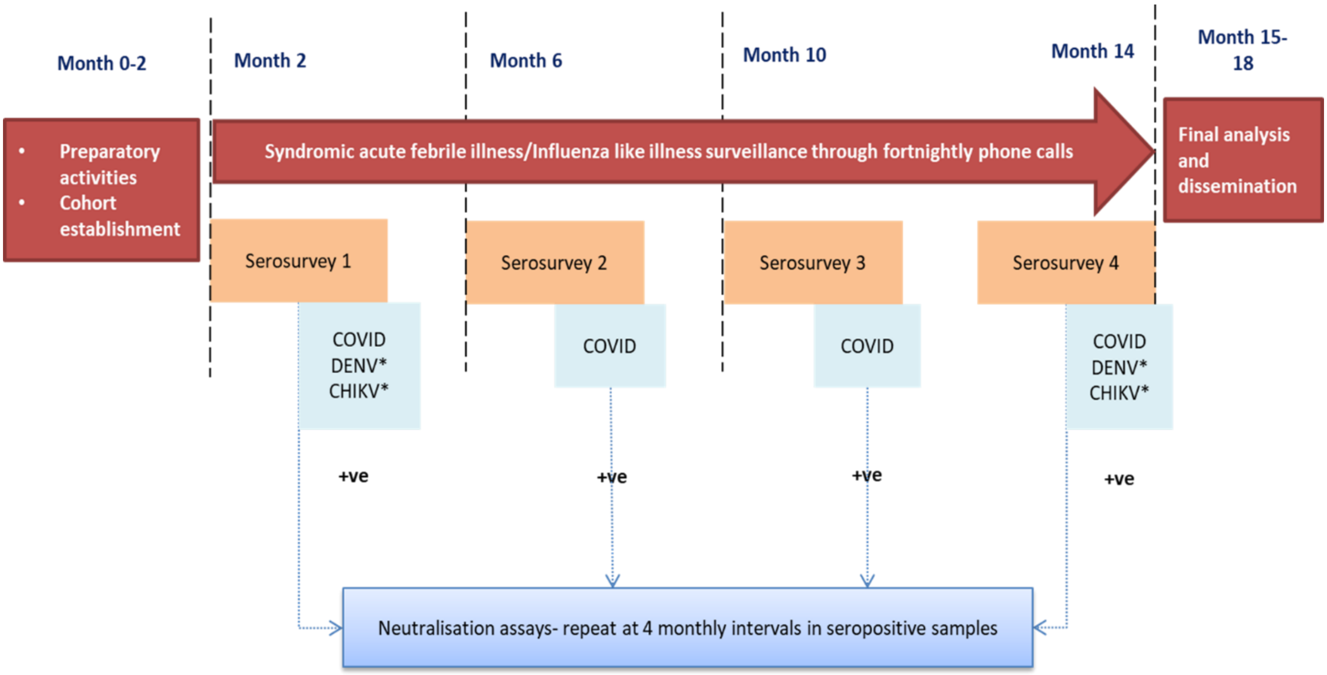

Figure 1: Overview: Flow Chart, testing plan and volume of samples required

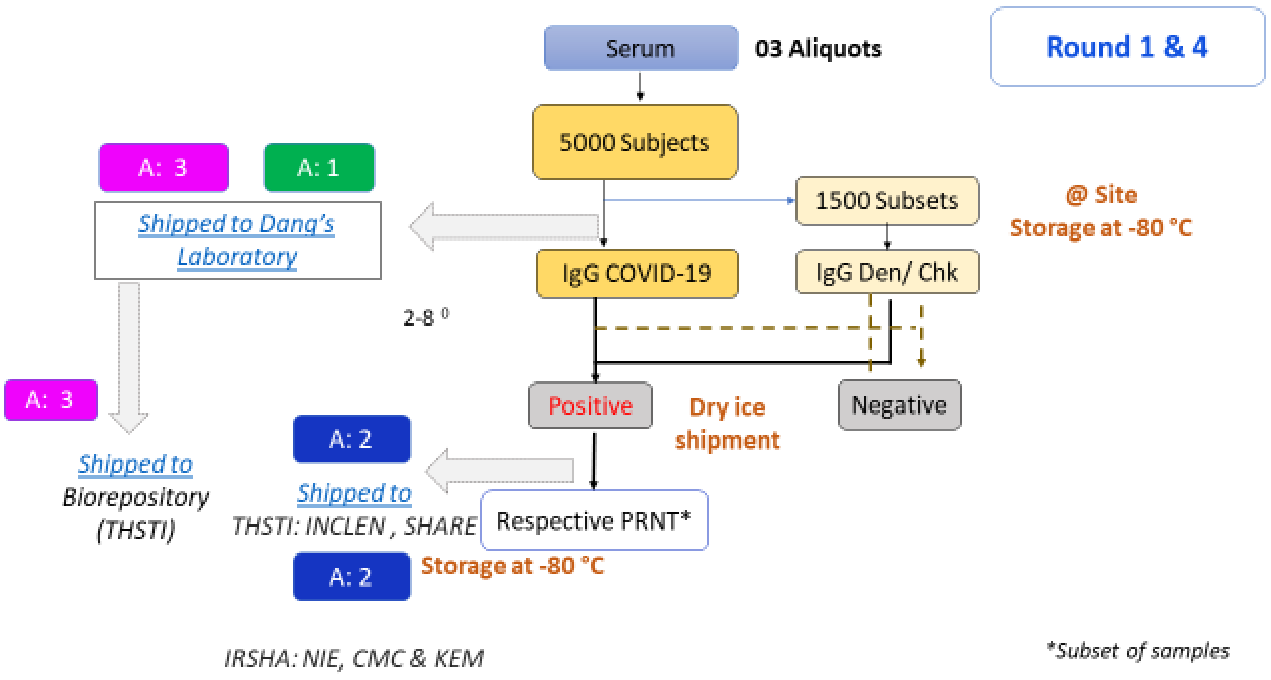

Figure 2: Pictorial representation of Sample Flow in Round 1 and Round 4

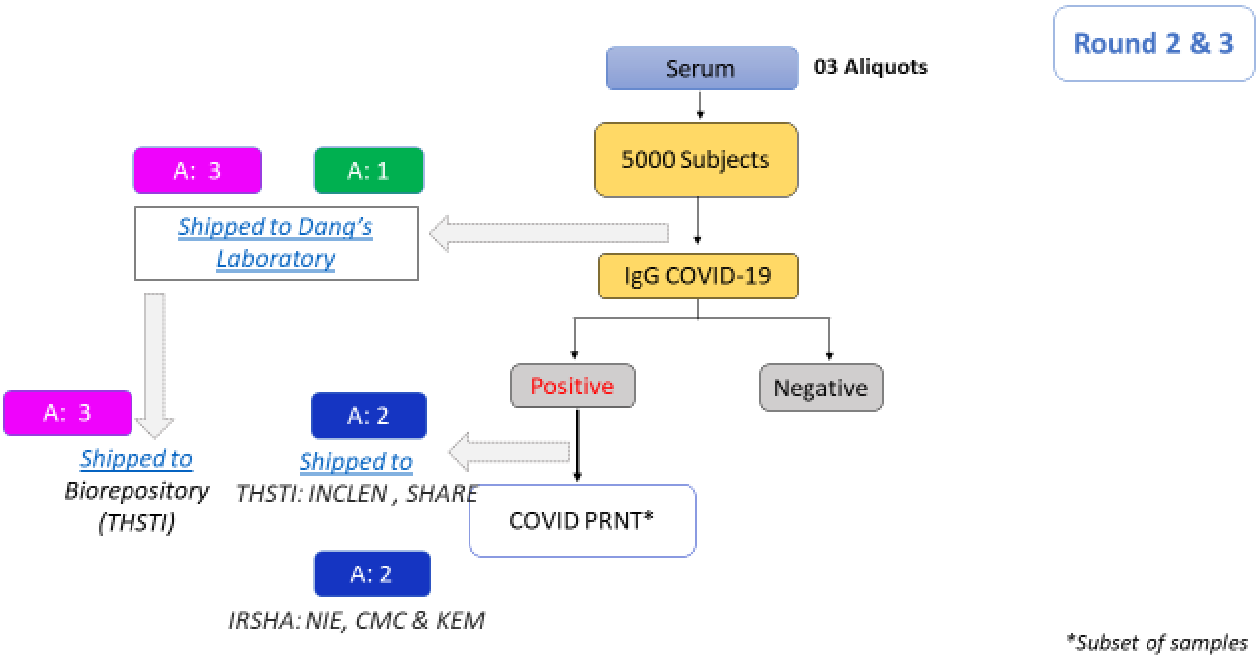

Figure 3: Pictorial representation of Sample Flow in Round 2 and Round 3

5.1 KEY NOTES FOR THE SAMPLE FLOW:

- Primary samples, secondary aliquots prepared and cryo-boxes shall be barcoded as per unique scheme to reflect round number, sample type, aliquot number. Pre-printed barcodes shall be used for accurate identification. *Refer Annexure 01 to understand labelling scheme*.
- Samples collected in the field (each site) shall be transferred to respective site offices except those for CMC site which shall be transferred from field to its respective departments further processing, testing and storage in all the four rounds.
- Blood samples for Serology shall be transferred to respective field offices for necessary processing, packaging and shipping including EDTA tubes for HbA1c testing.
- Three (03): Aliquot 1, Aliquot 2 and Aliquot 3 shall be prepared as per specified volume requirements and shall be stored refrigerated (Aliquot 1 & 3)/frozen (Aliquot 2) at site lab until despatch to respective lab location.
- EDTA tubes (stored in ~4 inches height cardboard box) as well as with Aliquot 1 in corresponding cardboard storage boxes (~3 inches height box) shall be maintained/stored refrigerated at 2-8 ^{0}C at sites and shipped to Dr Dang's Lab at 2-8 degree Celsius, with cold packs every alternate day.
- Aliquot 3 will be stored and shipped in fibre cryoboxes -) as recommended for biorepository.
- These shipments (Aliquot 1 and 3) will be picked by the Dang's Laboratory identified logistic services maintaining the shipment manifest in EDC. However, CMC will process EDTA and aliquot 1 sample at their dedicated facility whereas Aliquot 3 from CMC site will be shipped directly to THSTI on dry ice for biorepository.
- Once shipment is received at Dr Dang's Lab, Aliquot 1 will be processed for IgG ELISA investigations whereas Aliquot 3 will be further despatched (by road) to THSTI at 2-8 ^{0}C for

Biorepository following defined standard operating procedures. Only for Round 1, IgG testing shall be in staggered manner where in SARS CoV2 IgG testing shall be carried out initially and Deng/Chkn IgG testing shall be at a later date. Sera aliquots shall be maintained frozen after initial testing at Dr Dangs Lab unless testing completed. Any further storage required shall be as agreed.

- Aliquot 2 will NOT be shipped to Dangs lab, rather it will be stored at site at -80 ^{0}C in a pre-labelled cryo-box till ELISAs are tested for seropositivity.
- Based on the x% of seropositivity from ELISA, only selected aliquot 2 samples will be retrieved (based on box lay outs and available location in freezer) to be shipped to IRSHA/THSTI (central laboratory) in separate pre-labelled cardboard cryoboxes, on dry ice, for PRNT90 assays. Remaining samples will remain at site in the parent cryo-box for future archival/research studies.
- Necessary checks during entire sample life cycle including collection, processing, aliquoting, transfer, reporting and archival must be defined and implemented by each site to assure the quality.

5.2 DETAILS OF INVESTIGATIONS/ASSAYS PLANNED:

Blood samples shall be collected for testing from the participants as per defined plan in this manual basis ag=e groups and rounds. These shall be processed and each sample will be aliquoted into three (wherever possible) for further testing as below:

- Three (03) aliquots – aliquot1/2/3 will be prepared at site office laboratory for all the 5000 subjects in Round 1.
- SARS-CoV-2 IgG test shall be performed from Aliquot 1 for all the eligible subjects (all age group) in serial serosurvey rounds (R1/R2/R3/R4).
- Out of these 5000 subjects enrolled in Round 1, IgG test for Chikungunya and Dengue shall be performed from aliquot 1 in a subset of 1500 subjects from each site for neutralization assays only in Round 1 and 4.
- For these 1500 subjects, 1500 households (1 per HH) shall be randomly selected and 1500 would have equal representation from 2-5 years, 6-17 years and 18-60 years age groups and also between males and females. The sample size of 1500 will be based on the incidence of fever and required 12000 person years of follow up (all sites combined).
- The contiguous households will be enrolled in 5 clusters to achieve a sample size of 5000.
- All eligible and consenting individuals in a HH will be recruited. Assuming a HH size of 4, expecting 1500 HHs to be enrolled in each site.
- In certain circumstances where HH size is 6-7, 2 individuals shall be selected to achieve the required sample size of 1500 per site.

Testing network:

- For HbA1c - Dr Dang’s Lab (Delhi) shall receive whole blood samples in EDTA tubes for glycated haemoglobin testing from all sites except CMC (performed in-house).
- For IgG ELISAs - Dr Dang’s Lab (Delhi) shall receive sera samples (aliquot 1/400µL) for serology testing from all sites except CMC (performed in-house).

- For PRNT assays - IRSHA (Pune) and THSTI (Faridabad) shall receive only selected samples (aliquot 2/800 µL) based on x% of seropositivity directly from NIE, KEMHRC & CMC and for INCLEN & SHARE India respectively.
- For Biorepository - THSTI, Faridabad shall receive samples (aliquot 3/ leftover volume µL) from Dr Dang's Lab for INCLEN, SHARE India, NIE and KEMHRC. CMC will send directly to THSTI.

Note: *For Bio-repository at THSTI, all the aliquot 3 samples will be picked by Dr Dang's lab along with Aliquots 1 @ 2-8 ^{0}C. The shipment shall be further dispatched from Dr Dangs Lab to THSTI (Faridabad) on the same day, Dr Dangs Lab receives it. This is purely for the ease of dispatch since the two locations are in vicinity. This does not apply to any shipments from CMC where in the shipment for Bio-repository (cryo-box type 03) shall be directly sent to THSTI. The cryo-boxes will be color-coded for ease of identification (blue for Aliquot 2 and white for Aliquot 3 while white cardboard box with cell dividers for Aliquot 1). Shipments shall have corresponding Sample Transfer Form, discussed under Packaging and Transport of Samples.*

- A subset of seropositive samples (SARS- CoV-2/Chikungunya/Dengue IgG) results (seropositive samples) shall be tested for respective neutralization assay (from Aliquot 2 at IRSHA/THSTI lab) basis selection of samples for testing as per defined strategy.

- The order of preference, in case of short samples, shall be aliquot 1 followed by aliquot 2 followed by aliquot 3. Any specific cases may be discussed by site with the agency for clarifications.
- In addition, following biochemical investigations shall be carried out:

  - Haemoglobin: both age groups across all rounds, using machine "Hemacue"
  - Random Blood Sugar (RBS): only for participants ≥18 years of age, in Round 2/3/4. This shall be done using Point of Care (POC) machine "Glucometer"
  - Glycosylated haemoglobin (HbA1C): only for participants ≥18 years of age in Round 1

***Note (s)*:**

- *The POC tests shall be done with the sample collected for laboratory testing. There will be no additional finger/heel pricks for these two tests.*
- *The site shall ensure that the instrument specific operating instructions are available and testing in field is carried out by trained staff.*
- *The instrument readings must be noted down and record for Hb and RBS shall be made available to the participant in a written format. This format with disclaimer on limitations of POC/statement that tests performed by POC method may vary from site to site.*
- *In case of abnormal readings or those clinically relevant values requiring necessary medical intervention, the site PI may advice necessary follow ups/treatments as deemed necessary. Such mechanisms are out of scope of this study and is left for individual site to implement as per organisational policy.*

Refer following tables for details on investigations and sample requirements:

Table 1: Laboratory assays to be performed in serial serosurvey rounds (All Age Groups)
Table 2: Biochemical investigations in serial serosurvey rounds
Table 3: Blood volumes required for <18 years age group
Table 4: Blood volumes required for ≥18 years age group

Table 1: Laboratory assays to be performed in serial serosurvey rounds (All Age Groups)

Rounds	SARS-CoV-2 IgG ELISA	SARS-CoV-2 Neutralization assays	Dengue IgG and (Dengue Nt)	Chikungunya IgG & Neutralization assays
Round 1	✓	✓ (in a subset of SP samples)	For 1500 subjects (In subset of SP)	For 1500 subjects (In subset of SP)
Round 2	✓	✓ (in a subset of sample of SP of R1 and R2)	X	X
Round 3	✓	✓ (in a subset of sample of SP of R1 and R2 and R3)	X	X
Round 4	✓	✓ (in a subset of sample of SP of R1 and R2 and R3 and R4)	For 1500 subjects (In subset of SP)	In SN of Round 1 (In subset of SP)

SP = Seropositive in the current or previous round; SN-Seronegative in the previous round

Biochemical investigations

Table 2: Biochemical investigations in serial serosurvey rounds

Rounds	Age groups (<18)	Age groups (≥18)
Round 1	*Hb	*Hb, **HbA1c
Round 2	*Hb	*Hb #RBS
Round 3	*Hb	*Hb, #RBS
Round 4	*Hb	*Hb, #RBS

**POC by Haemacue; #POC by Glucometer; **Laboratory test*

Table 3: Blood volumes required for <18 years age group

Investigation	Matrix	Laboratory	Round 1	Round 2	Round 3	Round 4
POC-Hb	WB	POC#	1 drop	1 drop	1 drop	1 drop

Serology (COVID IgG & Chkn IgG & Den IgG)	Serum	**NABL Labs:** Dr Dang's lab* / CMC	400µL	400µL	400µL	400µL
Neutralization assay SARS-CoV2/Den /Chkn	Serum	**Central Lab:** IRSHA$^{\$\$}$/ THSTI$^{\$}$	800µL	800µL	800µL	800µL
Biorepository	Serum	THSTI	Leftover serum	Leftover serum	Leftover serum	Leftover serum

**Samples from NIE, KEM, INCLEN and SHARE*

$^{\$\$}$Samples from NIE, KEM, CMC; $^{\$}$Samples from INCLEN, SHARE

Shipments to THSTI Faridabad Bio-repository shall be picked up by Dr Dang's Laboratory (DDL) along with serology shipment, for all sites except from CMC which will ship directly to THSTI

Table 4: Blood volumes required for ≥18 years age group

Investigation	Matrix	Laboratory	Round 1	Round 2	Round 3	Round 4
POC-Hb	WB	POC#	1 drop	1 drop	1 drop	1 drop
HbA1c	WB	Dr Dang's lab* / CMC	2mL	-	-	-
RBS	WB	POC	-	1drop	1drop	1drop
Serology (COVID IgG & Chkn IgG & Den IgG)	Serum	Dr Dang's lab* / CMC	400µL	400µL	400µL	400µL
Neutralization assay SARS-CoV2/Den/Chkn	Serum	**Central Lab:** IRSHA$^{\$}$/ THSTI$^{\$\$}$	800µL	800µL	800µL	800µL
Biorepository	Serum	THSTI	Leftover serum	Leftover serum	Leftover serum	Leftover serum

**Samples from NIE, KEM, INCLEN and SHARE*

$^{\$}$Samples from NIE, KEM, CMC; $^{\$\$}$Samples from INCLEN, SHARE

Note: Shipments to THSTI Faridabad Bio-repository shall be picked up by Dr Dangs Laboratory (DDL) along with serology shipment, for all sites except from CMC which will ship directly to THSTI Faridabad.

6 SAMPLE MANAGEMENT THROUGH LIFE CYCLE:

Collection, Transfer, Barcoding, Processing, Aliquoting, Storage, Packaging and Shipment/ Transfer to respective laboratory

6.1 Preparation of sample collection kits:

Sample collection process is dependent on test required and the accuracy and timeliness of test results begin with a successful sample collection. The field staff must ensure that necessary supplies are available in adequate numbers for each day. The staff will familiarise himself/herself with the schedule of testing as per round and age group.

The table below will guide the staff preparing blood sample collection kits. There will be three types of kits and should be accordingly labelled for easy identification and ready availability:

- Children < 18 years
- Adult ≥ 18 Years; Round 1
- Adult ≥ 18 Years; Round 2-4

In addition to these kits, the field worker should also carry with them color coded bags and sharps proof container/needle cutter (for sharps disposal) to collect the BMW in each round.

	**Label: Children (Age <18 years)**
1	**Needle:** winged steel needle, preferably 22 or 23 gauge, with an extension tube (a butterfly), Safety Lock Butterfly Needle 23G **Catalogue # CAT 367292**
2	**Syringe:** BD 2 Pc 5ml Discardit II™ Syringe (With Needle) Cat# 300852
3	**BD Vacutainer® Plus Plastic Serum Blood Collection Tubes:**<br>4 mL Red Top (BD catalogue # 367812)
4	**Spirit Swab**

	**Label: Adult (Age ≥ 18 Years) R1**
**1**	**Needle:** 21G x 1"
**2**	**Syringe:** BD 2 Pc 10ml Discardit II™ Syringe (With needle) Cat# 300294
**3**	**BD Vacutainer® Plus Plastic Serum Blood Collection Tubes:**<br>5 mL Red Top (BD catalogue # 367814)<br>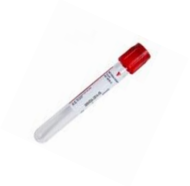
**4**	**Spirit Swab**
**5**	EDTA coated tube: 2mL in purple top<br>**(BD Catalogue# 367841)** 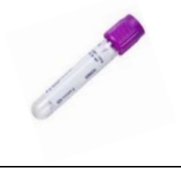

**Round**	**Label: Adult R 2-4**
1	**Needle:** 21G x 1"
2	**Syringe:** BD 2 Pc 10ml Discardit II™ Syringe (With needle) Cat# 300294
3	**BD Vacutainer® Plus Plastic Serum Blood Collection Tubes:**<br>5 ml Red Top (BD catalogue # 367814)
4	**Spirit Swab**

The catalogue numbers specified are the ones to be used during the study. The inventory and supplies should be maintained accordingly. For paediatric phlebotomy, 23g Safety Lock Butterfly needles may be used as necessary or safe phlebotomy; BD Catalogue # 367292.

- *The **BD Vacutainer® Plus Plastic Serum Blood Collection Tubes** contain silica act clot activator. After collection, gently invert the tubes 5-6 times.*
- *BD Vacutainer® spray-coated K2EDTA Tubes are used for whole blood hematology determinations. Gently invert the tubes 7-8 times immediately after collection.*
- *DO NOT SHAKE the tubes*

6.2 Sample collection and reporting in field (Home)

6.2.1 Volume collection

The collection procedure is detailed in section 8.1. Volume of the blood sample to be collected in each serosurvey in each round based on the age of the participant is as follows. The volumes have been rounded off for sake of simplicity.

Table 5: Volume of blood sample required for performing tests in serial serosurvey rounds

Rounds	Age groups (<18)	Age groups (>=18)
R1	4mL	8mL
R2	4mL	6mL
R3	4mL	6mL
R4	4mL	6mL

Note:

- *Minimum volume of blood to be drawn is 2.5mL. If unable to collect the required volume, then prick another site for collecting the blood sample as per requirement. 6/8 ml blood volume is indicated to allow transfer to **BD Vacutainer® Plus Plastic Serum Blood Collection Tubes** /EDTA and for RBS/Hb.*
- *Fill up the tubes to the line indicated on tube (volume mark).*
- *After transferring the blood, recap and secure, place upright to avoid spillage.*

6.2.2 Labelling

Accurately and real time labelling of blood samples is a pre-requisite for generating reliable results. The blood sample tube will be labelled by the phlebotomist using a pre-printed barcode. Refer Annexure 01: Barcode Label for Primary Tubes/Aliquots and Cryo-boxes for scheme of assigning barcodes for vacutainer, three (03) types of aliquots and respective cryoboxes for dispatch to designated laboratory. Phlebotomist shall arrange the sequence of tubes prior to collection at a household and verify identity at time of collection. After collection of samples, the field worker will scan the barcode on the sample tube. This will get automatically linked with the participant identification number (PID) once the data is entered in electronic data collection platform (EDC) – SOMAARTH-3. These barcodes will be 9 digits unique identifiers which will

be generated for each sample linked with the PID. Refer Figure 4 and 5 for printed barcodes and their alignment on tubes.

Figure 4: Barcode Labelling Scheme

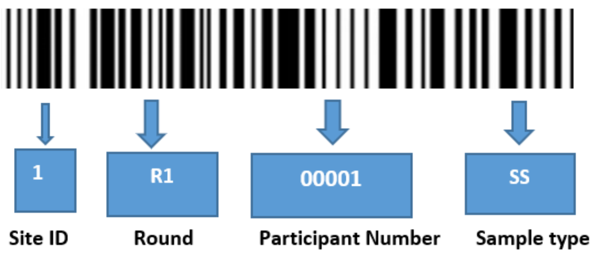

Figure 5: Barcode placement on primary tube

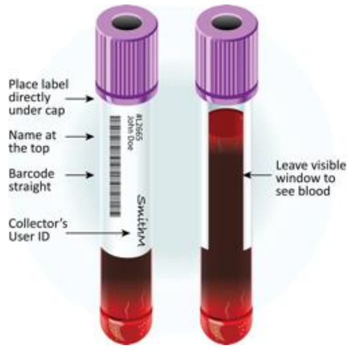

Note*:*

The barcodes shall be pasted vertically and aligned in such a manner to allow seeing the blood. Refer figure 4. Barcodes are software generated. These barcodes will be printed in the office site laboratory as per recommended specifications. All tubes to be placed in the Sample shipment container in the racks so as to avoid any spillage. Refer Annexure 01 for details on Barcode label scheme for Primary Tubes, Aliquots and & Sample types/Cryo boxes**.**

6.2.3 Testing and reporting in field (home)

- **Testing of samples less than 18 years:** Field worker will test the whole blood sample collected at the participant site for Hb using a Haemacue instrument.
- **Testing of samples in (>=18years):** Field worker will test the whole blood sample collected at the participant site for Hb using a Hemacue and RBS using a glucometer instrument.
- **Reporting:** Field worker will fill in the report immediately once the samples are tested for Hb in round 1; and Hb & RBS in rounds 2, 3 & 4 respectively. These measurements will be shared with the participants.

Refer section 5.2.6 and section 8.1.2/8/1.3 for additional notes on Hb testing and reporting using Haemacue and RBS using Glucometer.

6.3 Sample management at Field office

6.3.1 Sample transfer from field and receipt at field office

- After each day of collection completed, samples shall be transferred to field office. It is expected that samples shall reach by 4:30 PM on each day of collection, considering the site strategy and distance of travel, sample to be processed for further aliquoting and storage at 2-8 ^{0}C or at -80 ^{0}C in freezers.

- For each site, the samples from field will be transferred refrigerated in vaccine carrier. Utmost care will be taken to ensure sample integrity. Tubes will be placed in upright position. Red top tubes shall be allowed to clot before being placed in vaccine carrier (usually 30-45 minutes are adequate for blood clotting)
- Samples shall be positioned so as to be spillage proof. Utmost care, as feasible, will be taken to avoid jerks/shocks to samples while in transit.
- No documents/machine/supplies should be placed along with biological samples. Gel packs shall be placed in adequate numbers to ensure temperature is maintained post collection till receipt in field office.
- Collection details and transfer to field will be documented in the software so as to allow complete trail of sample movement. The software will allow the data capture related to each sample collected on that particular day and data shall be uploaded once synced.

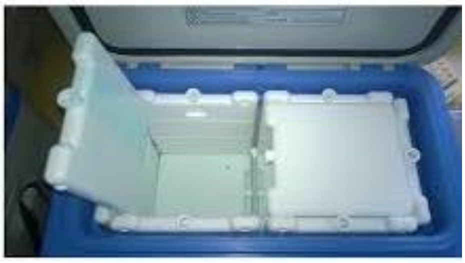

Figure 6: Vaccine carrier box

Note: *No sample shall be kept in vaccine carrier box without transfer and handover to the designated person in field office. Sample accountability is site's staff responsibility.*

6.3.2 Separation –

- Before samples arrive physically, information will be available to the field office staff regarding number of samples collected and the expected time of arrival. Site will develop robust communication systems on information flow.
- The information from EDC software will be utilised to arrange the required number of cryo-vials and arrange in sequential order. A second level check is required to ensure the sequence is followed.
- Each site may adopt their best practices to ensure batch arrangement, accurate aliquot information and pipetting. One suggested way is to arrange three cryo-vials for one corresponding in-coming **BD Vacutainer® Plus Plastic Serum Blood Collection Tubes** followed by an empty position and then three for the next sample in sequence. Other way is to arrange three types of cryo-vials (basis aliquot volume and destination lab in three different racks in same sequential order as the **BD Vacutainer® Plus Plastic Serum Blood Collection Tubes**. It is upto the site to review this and come up with the best arrangement. The objective is to ensure serum sample is aliquoted and stored into corresponding cryo-box.

- Ensure that the refrigerated centrifuge is functional, necessary equipment is assembled, barcodes are available and freezer displays required temperature. Any malfunction should be proactively addressed. Sample integrity should be ensured.
- Once the samples reach the site lab, count the number and type of tubes physically received. Cross check against the numbers reflected in the software and communicate. Acknowledge each incoming sample in the EDC. Raise an alert for a missing sample or issue with sample integrity to allow early redressal. Each site will monitor the set of indicators to allow prevention of such incidences, if any.
- Inspect the tubes (**BD Vacutainer® Plus Plastic Serum Blood Collection Tubes** and EDTA, as applicable in round 1) for any breakage/leakage/missing barcode/soiled or damaged barcode during transit. Visually inspect volumes and placement of barcodes.
- Note down any observation for further discussion, follow ups and corrective/preventive action as may be necessary. Any consistent low volumes or problems encountered in collected samples by a particular phlebotomist at a site should trigger a re-training to ensure the issue resolution. The threshold may be defined by the sites for such activities and monitored as part of quality control.
- Site should develop checklist to ensure the transfer and receipt process is efficient and smooth. Document any problem sample (breakage/leakage/spillage/empty tube etc as **problem sample** for further information and management.
- The collection details shall be available in EDC. Further, receiving, sample processing and aliquoting shall also be captured in EDC. Ensure timely completion and accuracy of data entry into the EDC. Site will have designated senior team members to monitor this activity in live phase to avert any potential errors/issues. Sites may have additional logs to capture information such as necessary that is not available in EDC to allow trail of activities.
- Place the **BD Vacutainer® Plus Plastic Serum Blood Collection Tubes** in refrigerated centrifuge and operate it as per work instructions. Serum has to be separated within 24 hours, at 3000 revolutions per minute (RPM) for 10 minutes @4 ^{0}C.
- Process samples centrifuged together as a batch so that while the next batch is in centrifuge, the processed samples may be aliquoted, verified and logged-in to designated refrigerator (aliquot 1/3/EDTA) or Freezer (Aliquot 2).
- Complete all entries in EDC on a real time basis to allow all samples collected on the same day to be processed, aliquoted and stored.

6.3.3 Aliquots

- Using calibrated micro pipettes, dispense required sera volumes as per defined procedure (refer Table 6 and Table 7) in three labelled cryo-vials for a participant- Aliquot 1 (A1) 400 µL, Aliquot 2 (A2) 800 µL and Aliquot 3 (A3) left over sera. Cryo-vial reference *Tarsons, catalogue# 523020 should be used. Similar*- may be used that is suitable for the testing, shipment and long term storage purpose. Aliquots will be labelled using a pre-printed barcode which will link with the PID. (**See Annexure**

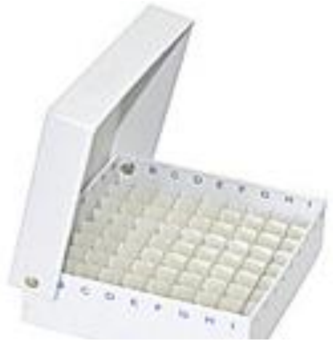

Figure 7: Cardboard box with layout

01). Maintain box lay out for each cryo-box to allow easy retrieval on a later date.

- Record number of aliquots prepared as 03 (Yes/No) and completes the other details in EDC.
- Place each of the 03 Aliquots prepared in a designated corresponding labelled cardboard/cryo-box (see Annexure 01: Barcode Label for Primary tubes/aliquots and Cryo-boxes). Document in real time manner. . The cryo boxes will be colour coded for ease of reference:
  - ➢ Aliquot 1 in White cardboard with cell divider (10x10 or smaller capacity; box height 3 inches) box preferably with layout (see Figure 7).
  - ➢ Aliquot 2 in Blue fibre cryo-box for storage at site and White cardboard box (10x10) for further shipment from to IRSHA/THSTI when samples requested.
  - ➢ Aliquot 3 in white fibre cryo-box
- Count the number of cryo-boxes and record login details of cryo-boxes in refrigerator (Aliquot 1 & 3) and Freezer (Aliquot 2). Login EDTA samples also at 2-8 ^{0}C along with Aliquots 1 & 3 placed

  in cardboard or cryo boxes (respectively) and stored refrigerated. Record signature, time and place assigned in refrigerator/freezer with the equipment ID.

  Each site will complete this for the sample management in EDC and associated site specific logs. Any comments/remarks may be noted. While preparing the aliquots, place the vials in the cryoboxes as recommended. -
- Scenario 1: When cryo-box is fully filled i.e. all 100 slots are occupied (A1 to J10 OR 01 to 100), as information is available in Box Lay Out, store the subsequent cryo-vials in a new cryo-box containing respective aliquot1/2/3. Maintain the logs as recommended with cryo-box no., aliquot no. and slot.
- Scenario 2: When cryo-box is semi-filled i.e. 5-10 slots or more are vacant in a particular batch– keep the aliquots in a dummy cryo-box while separating in the consecutive batch of respective aliquots. Take this dummy cryo-box near to freezer and refill the vacant slots in the cryo-box until it is fully filled. Record the time and temperature during the point of exit and entry of parent cryo-box into the refrigerator/freezer. Any variations should be recorded in the site specific freezer access record logs -
- Follow **Good Documentation Practices**. Do not over write. For error correction, strike with a single line, re-write and provide dated initials. Wherever possible, blue ink is recommended. Site staff should be trained in documentation handling and data capture.
- Maintain relevant logs and records for each day of sample handling.

Three aliquots for each sample collected in **BD Vacutainer® Plus Plastic Serum Blood Collection Tubes** will be prepared. Lesser number of aliquots may be obtained in case of short samples. The preferred order of aliquots is 1, followed by 2 and 3. All efforts shall be made to prepare all three aliquots with volume defined. Although cryo-vial capacity is 1.8 ml, it should not be filled beyond 1.2 ml. Remaining surplus volume may be prepared extra and labelled should the site face such situation. Pipettes of appropriate capacity may be used for aliquot preparation. For instance pipette set at 400 μL may be used to dispense Aliquot 1 and twice for Aliquot 3, thrice for Aliquot 3 etc. This is left to the technical judgement of competence of site. **Sample collected in EDTA vial will not be aliquoted. However, ensure to secure the EDTA**

tubes with a micropore tape to ensure these are spill proof. store the prepared aliquots and EDTA tube in respective boxes under specified temperature conditions unless shipment is scheduled.

**Table 6: Aliquots and shipment >=18 years age group**						
**Aliquot**	**Matrix**	**Laboratory**	**Round 1**	**Round 2**	**Round 3**	**Round 4**
Aliquot 1	Serum	Dr Dang's lab / CMC	400 µL	400 µL	400 µL	400µL
Aliquot 2	Serum	**Central Lab:** IRSHA$/ THSTI$$	800µL	800µL	800µL	800µL
Aliquot 3	Serum	Dr Dang's Lab/ THSTI#	Leftover serum	Leftover serum	Leftover serum	Leftover serum
EDTA	WB	Dr Dang's lab / CMC	Sample collected	-	-	-

#From CMC directly to THSTI while for other sites dispatched to THSTI routed via Dangs Lab
$Samples from NIE, KEM, CMC;
$$Samples from INCLEN, SHARE

**Table 7: Aliquots and shipment <18 years age group**						
**Aliquot**	**Matrix**	**Laboratory**	**Round 1**	**Round 2**	**Round 3**	**Round 4**
Aliquot 1	Serum	Dr Dang's lab / CMC	400µL	400µL	400µL	400µL
Aliquot 2	Serum	**Central Lab:** IRSHA$/ THSTI$$	800µL	800µL	800µL	800µL
Aliquot 3	Serum	Dr Dang's Lab/ THSTI#	Leftover serum	Leftover serum	Leftover serum	Leftover serum

# From CMC directly to THSTI
$Samples from NIE, KEM, CMC
$$Samples from INCLEN, SHARE
Note: Shipments to THSTI Faridabad Bio-repository shall be picked up by Dr Dangs Laboratory (DDL) along with serology shipment, for all sites except from CMC which will ship directly to THSTI Faridabad.

6.3.4 Storage at field office lab –

- Aliquot 1 will be stored in a cryo-vials (*Tarsons, catalogue# 523020 or ABDOS catalogue # P60109/P60110 or Merck – catalogue # BR114841 or similar) and store in* a white cardboard box (box height 3 inches) with layout display containing 100 cryo-vials (10x10) OR similar as per site requirement and feasibility based on recruitment rate. Smaller capacity storage boxes suitable for purpose may be used for best utilisation/efficiency. This information will be captured in EDC . Working checklists may be adopted by site to help decide if all samples could yield desired number of cryo-vials as per defined volumes.
- Aliquot 2 will be stored in a pre-labelled **blue** fibre cryo-box (*Tarsons, catalogue# 524020A or similar)* containing 100 cryo-vials (10x10) at @-80 ^{0}C (ultra -low freezer) at the site deep

freezer. Only selected A2 based on subset of SP will be sent to IRSHA/THSTI from the site in a separate cardboard box for PRNT assays. Remaining aliquots will be retained by the sites in the parent fibre cryo-box maintaining the sample access log. Such records shall be maintained to allow sample movement trail.

- Aliquot 3 will be stored refrigerated in **white** fibre cryo-box with layout display containing 100 cryovials (10x10) OR similar smaller capacity as per site requirement/ feasibility based on recruitment rate. These shipments will be picked up along with Aliquot 1 and EDTA (round 1). These will then be sent to THSTI for biorepository on the same day from Dr Dang's Lab, except CMC, as all A3 will be sent directly from CMC site to THSTI for biorepository.
- EDTA samples shall be stored refrigerated in a cardboard cryo-box (box height 4 inches with internal cell divider). Capacity of box may vary depending on number of samples and despatched under refrigeration with Aliquot 1 box to Dr Dangs Lab (shipment to be arranged by Dr Dangs Lab).
- As soon as the aliquots are prepared from a centrifuged sample, complete corresponding documentation (aliquot prepared, volume, any comments etc) in EDC and scan individual cryo-vial to assign position in the respective cryo-box. This will link each cryo-vial (A1/A2/A3) to a corresponding cryo-box in which it is placed. This information gets captured in EDC and shall help retrieve samples as and when required. The box position in a freezer shall be maintained at site level as per defined procedure. Situations such as sample not received (from field), sample quantity not sufficient, sample lost in transit etc shall be recorded in EDC or maintained by site to allow trail of events.
- Any variation in the recommended temperature has to be recorded in a temperature log.
- Adequacy of aliquot volume will be recorded in the EDC while shipping from the origin (site/ Dangs lab) and destination (Dang's Lab and THSTI) or any such situation that test may not be performed/reported, for instance any sample lost in transit.
- The site will have mechanism to address any contingency.
- The cryo-box (Aliquot 2 & 3) will be colour coded for ease of identification. The ones with hinges on one side and open on orher are preferred to allow correct placement of lid/sample positioning.

6.3.5 Cryo-box Label:

- Ensure to paste the barcode label meant for cryo box on the lower/base box and not the lid to allow accurate identification. Define the order of placement of cryo-vials in a box (left to right) and mark the direction to match box layout.
- Mark the alignment mark on base and lid to allow correct closure/placement**.**
- Aliquot 1 of all participants to be placed/stored in corresponding cardboard cryo-box and barcode label to be affixed. Label on this cryo-box to be **Aliquot 1 (AQ1)**
- Aliquot 2 of all participants to be placed/stored in one cryo-box and label on this cryo-box to be **Aliquot 2 (AQ2)**
- Aliquot 3 of all participants to be stored in one cryo-box and label on this cryo-box to be **Aliquot 3 (AQ3)**
- **Box lay shall be maintained in EDC for each box to allow easy access/retrieval/despatch.**

6.3.6 Packaging for Shipment

The refrigerated shipment is scheduled (Aliquot 1 and Aliquot 3, along with EDTA samples in round 1) every alternate day from site by the Dang's Lab as per information below:

- Aliquot 1: To Dr Dang's Lab/ CMC
- Aliquot 2: To IRSHA/ THSTI
- Aliquot 3: To Dr Dang's lab (except for CMC which is directly shipped to THSTI)

- Once scheduled, The samples in corresponding box shall be retrieved and shipment will be prepared as per despatch locations.
- Complete despatch details in EDC. ***DR DANG'S LAB WILL BE RESPONSIBLE FOR TRANSFER OF SAMPLES OF CRYOBOX LABELLED AS 'A3' ALONG WITH THEIR SHIPMENT FORMS TO THSTI. CLOSE COORDINATION WIL BE EXERCISED BY DANGS LAB AND SITE FOR TIMELY PICK UP OF SHIPMENT AS PER AGREED DAYS/CUTOFFS.***

- Record box despatch in EDC _3along with waybill number. Each site shall ensure despatch as per destination location:

  - Dr Dang's Lab/ CMC; IRSHA/ THSTI; THSTI Bio repository (shipped with Dr Dangs Lab, except CMC which will ship to THSTI directly).

6.3.7 Shipment receipt at Dr Dangs Lab

- Once shipment is despatched from site, information n box and its content is available to the laboratory. The information in form of an excel may be downloaded that will be further used for accessioning into LIMS at Dr Dangs Lab. Shipment may be tracked/traced through waybill number.

  Note: CMC lab shall ensure the samples received from the site locally are handled accordingly.
- Once sample shipment is received at Dr Dangs Lab, it shall acknowledge in EDC about contents and condition at receipt. All sample list with corresponding box shall be checked.
- The shipment with Aliquot 3 received at Dr Dangs Lab shall be as it is forwarded (without opening the box) to THSTI bio repository by local transport to reach same day as received.
- At Dangs Lab, the samples shall be handled, tested and reported as per lab's procedures. Samples shall be tested in a staggered manner in Round 1 where in SARS-Cov2 IgG will be tested initially and aliquot shall be frozen unless testing trigger received for remaining two assays (Deng Chik IgG) to be performed on select participants. Remaining aliquots may be transferred to THSTI bio-repository if decided so. Samples tested will be disposed off as per directives/agreement with laboratory.

6.3.8 Sample Receipt at THSTI Biorepository

- Samples will be received from sites through Dr Dangs Lab except for CMC which shall ship samples directly to THSTI
- Once received, procedures established for sample handling shall be followed.
- Sera aliquots shall be frozen in qualified freezers and temperature monitored for the units
- Sub aliquoting of serum sample (aliquot 3) may be done if mandated. This could be taken up to allow for allocation of smaller volumes and multiple freeze thaw.
- Access committees may be established to direct this activity. Any request for an aliquot will be handled as per institutional procedure/study specific governance.

7 ASSAYS FOR COVID, DENGUE AND CHIKUNGUNYA

7.1 For SARS-CoV-2 IgG

All samples collected from 5000 subjects will be tested for IgG COVID quantitative estimation using Diasorin Ig Kit *LIAISON® SARS-CoV-2 S1/S2 IgG ([REF] 311450)*

A subset of the seropositive samples (seropositive for SARS-CoV-2 infection) during each of the serosurvey rounds will be tested for neutralizing antibodies using PRNT90 or micro neutralization assay at IRSHA or THSTI depending on the site of collection.

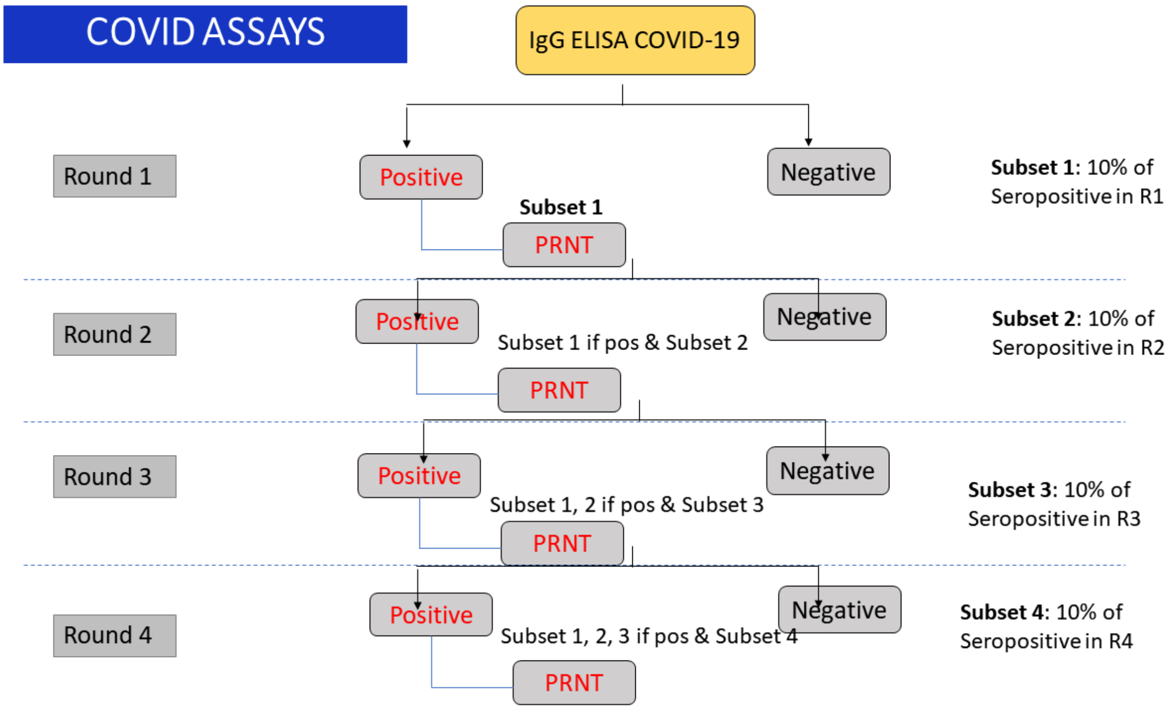

Figure 8: Investigation flow for COVID assay

In each round 10% of the seropositive samples will be tested for neutralizing antibodies. This constitutes a subset. Each new subset created of new seropositive(s) in each round 1, 2, or 3 will be followed till the last round and assessed for neutralizing antibodies.

7.2 For Dengue

The IgG testing will be done using the Panbio® Dengue IgG Indirect ELISA catalogue number 01PE30 kit. A subset of the seropositive samples (seropositive for Dengue infection) during the serosurvey round 1 will be tested for neutralizing antibodies using PRNT90.

In round 1, 10% of the seropositive samples will be tested for neutralizing antibodies. This constitutes subset 1. In round 4, 10% of the new seropositive samples along with those from subset 1 will be assessed for neutralizing antibodies.

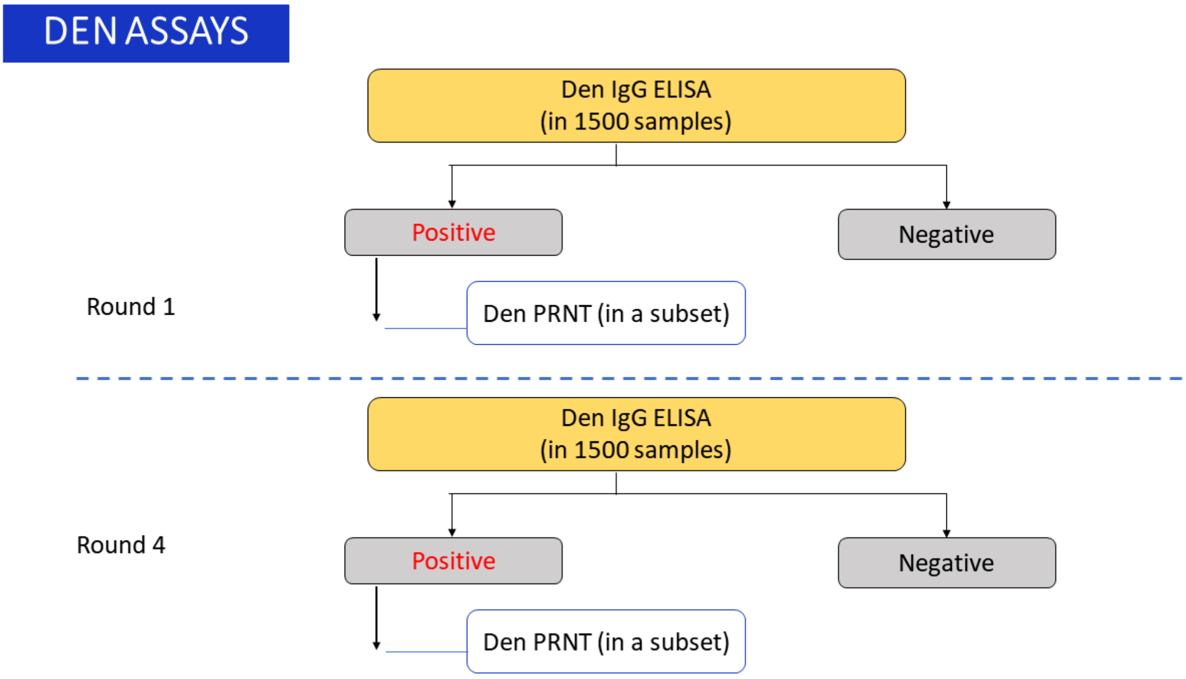

Figure -9: Illustration for IgG ELISA for Dengue assays

7.3 For Chikungunya

The IgG testing will be done the using the using the **InBios International Inc catalogue # as CHKG-C**

A subset of the seropositive samples (seropositive for Chikungunya infection) during the serosurvey round 1 will be tested for neutralizing antibodies using PRNT90.

In round 1, 10% of the seropositive samples will be tested for neutralizing antibodies. This constitutes subset 1. In round 4, 10% of the new seropositive samples and subset 1 will be assessed for neutralizing antibodies.

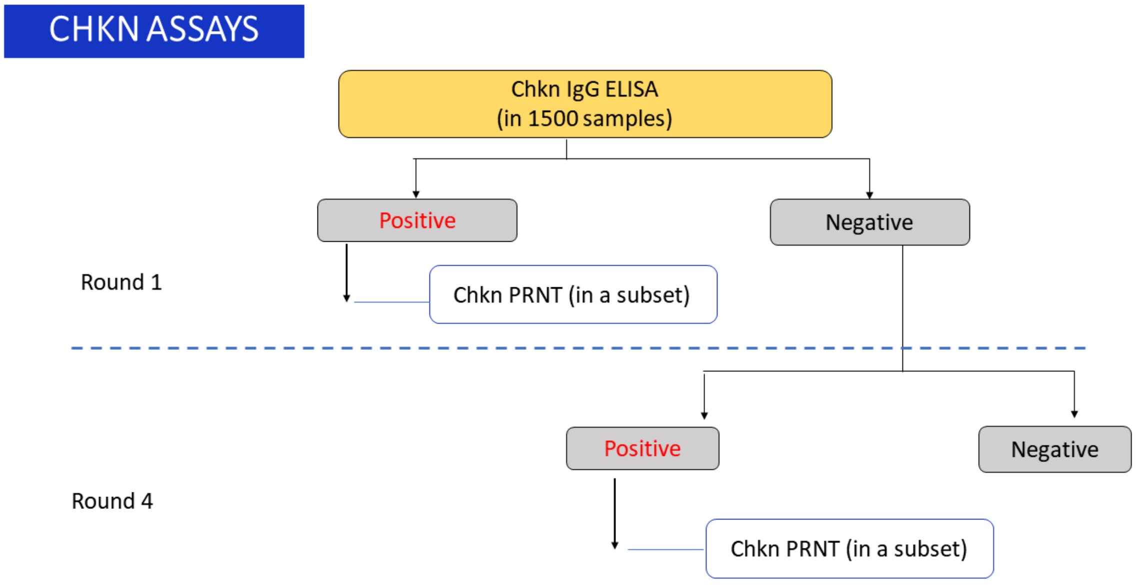

Figure -10: Illustration for IgG ELISA for Chikungunya assays

8 GENERAL GUIDELINES FOR GOOD LABORATORY PRACTICES

8.1 Sample collection

The procedures defined below are based on WHO guidelines on blood collection: best practices, 2010.

i. **Blood sample collection, equipment and collection methodology to be followed as mentioned in section 8**

ii. **Use of PPE:** The phlebotomist and the field workers should wear a surgical mask before entering the participant's house. There is no need to wear the whole PPE kit as we are not taking samples from symptomatic patients. The phlebotomist should perform hand hygiene with alcohol-based hand rub and then wear a plastic apron and gloves before blood draw.

iii. **Place of blood collection:** The blood collection will be performed at the participants' house after obtaining written informed consent. If there is any difficulty, the participant may be brought to the study office/clinic for blood collection. Ensure the following:

- Ask permission from a responsible member of the household to allow the team to use an appropriate room or space with adequate privacy for blood draw.

- Keep supplies ready and assembled before they are called into the room for sampling.
- If children are around, child proof the area so that no supplies are in the reach of the children.
- Pack and dispose all disposable supplies after use. Do not leave any equipment or supplies on the table or room of the participant. Decontaminate equipment as necessary.

8.1.1 Preparing the participant:

Make the participant comfortable. Ensure that the participant does not have anything in their mouth during the procedure to avoid choking hazard. Do not attempt blood draw on empty stomach. If the participant did not have his/her meal, please wait till he finishes. Explain the procedure to each participant, both adults and children. If the participant is a child, please allow one parent or a relative to be with the child during blood (if required). Treat each participant with respect and reassure them to avoid panic. Be always willing to clear any doubt they have regarding the procedure. Be gentle and kind with all participants, especially children. Keep in mind that participant safety is of utmost importance in the study and follow ethical principles.

i. **Position of the participants during blood draw** - Always have the participant lying or seated in a safe place. Most children below 6 years of age may need someone to hold their arm still. Firm support from a co-worker while anchoring the vein is recommended. Children can be either lying down or sitting on someone’s (preferably a parent) lap.

ii. **Identifying the place to puncture:** The recommended site of venepuncture is the ante cubital fossa. Tie tourniquet well above the elbow joint. Make sure that the knot of the tourniquet is not coming over the field. The best vein to use is the median cubital vein or the cephalic vein in the anterior cubital fossa. If you are using the basilic vein, please be careful not to injure the arteries and nerves around it. Dorsal veins of the hand could also be used but do not use the veins in the underside of the wrist. Once the vein is identified well, the field research assistant should hold the hand in correct position (for children).

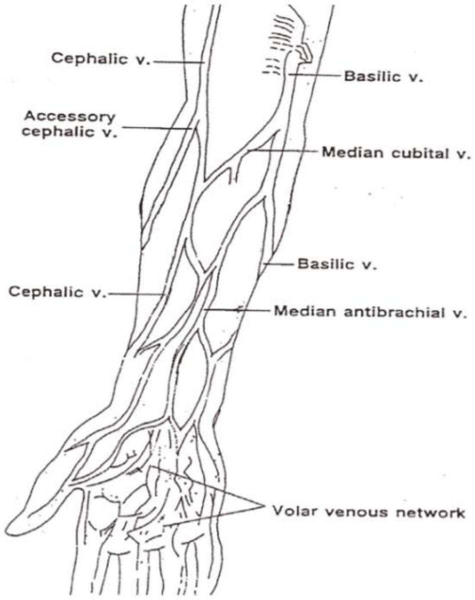

Figure 11: Identification vein to puncture for blood withdrawal

iii. **Cleaning of the skin:** Clean the area to puncture with cotton swab soaked with 70% alcohol (spirit). Cleaning should be done from centre to periphery at least 3 times. Make sure that the patient is relaxed.

iv. **Collection of blood:** Follow best phlebotomy practices including positive identification of participant, safe handling of blood, good labelling practices and appropriate disposal of bio waste. Following are key steps:

- Using a syringe and needle (as per specifications of tubes and recommended gauge needles), withdraw/collect the required amount of blood depending on the time of blood draw and age group as mentioned earlier in section 6.2.1.
- Transfer the collected blood into (leaving a drop each for Hb and RBS POC tests) respective **BD Vacutainer® Plus Plastic Serum Blood Collection Tubes** and / or EDTA tube. Label/barcode the primary tubes and gently invert to allow blood mix up with additives (5-6 times for **BD Vacutainer® Plus Plastic Serum Blood Collection Tubes** red top) and 7-8 times for EDTA purple top). Keep blood filled tubes upright and complete documentation. Accurate and timely data capture is critical.

Below pictures are only for reference and visual understanding:

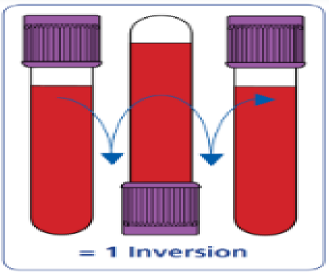

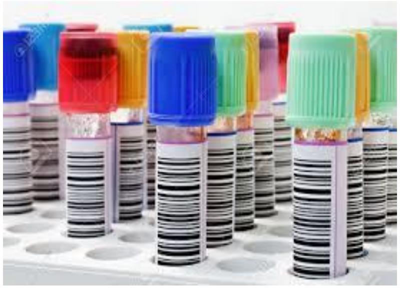

Figure 12: Sample collection and storage

- From the remaining amount, perform Hb and RBS, as the case may be, to obtain valid results. Note down the results for later sharing the report with participant.

Note:

- If the desired amount of blood is not available at a time, a second attempt can be made in another site after obtaining the participant's or parent's permission if the participant is a child. The cleaning procedure has to be repeated at the new site.
- Not more than 2 attempts will be made on the same day in case of a failed blood draw. If the participant is willing another attempt can be done after a gap of 48 hours with the help of experienced phlebotomist. If the attempt on the second day is also a failure, please do not poke the patient again until the next round of serosurvey.
- Ensure to note down the display results as the display will disappear after stipulated time. Ensure equipment is in functional status and results are reliable.

Please refer Figure 13 below for steps involved in blood withdrawal procedure.

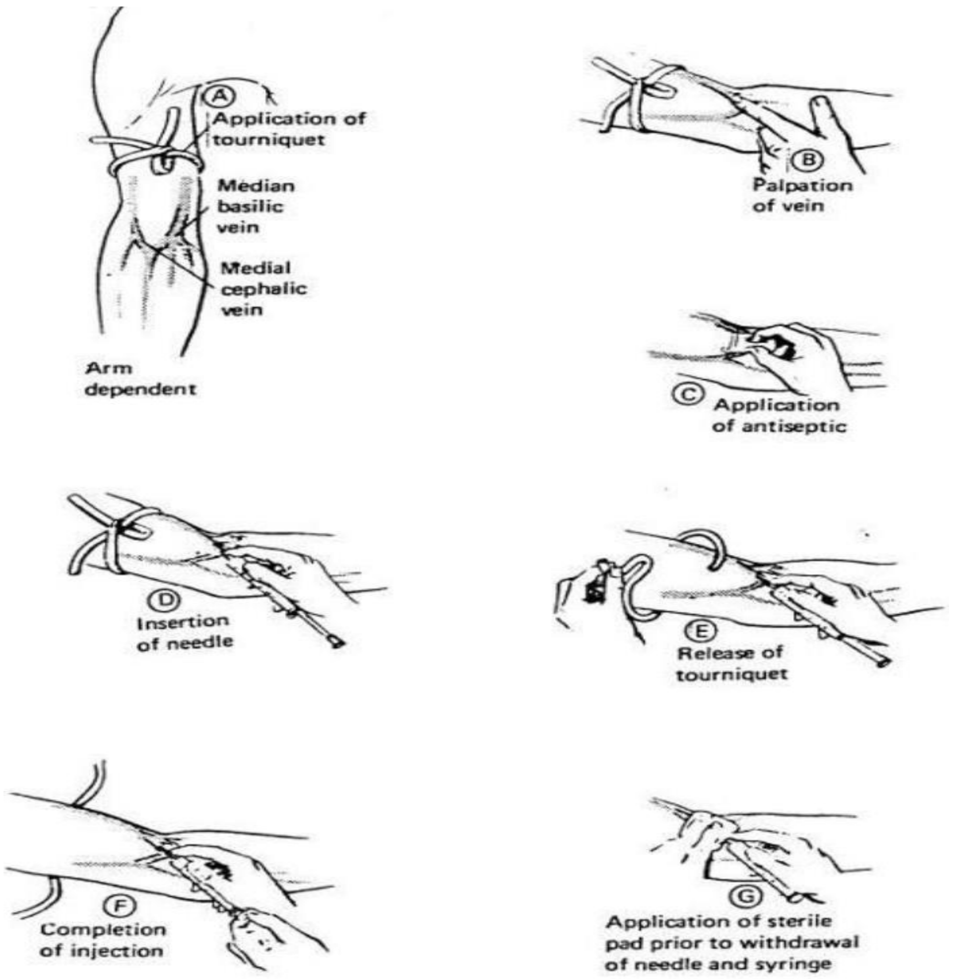

Figure 13: Steps for blood collection

8.1.2 Inoculation of blood into the Haemacue: Operate the machine as per manual/instructions

- The phlebotomist should first place a drop of blood from the syringe on the Haemacue micro cuvette and make sure that the drop of blood is large enough to fill it completely. Do not refill the micro cuvette.
- Wipe off the extra blood on the outside of the micro cuvette. Make a visual inspection of the micro cuvette.
- If the microcuvette has bubbles or if it is not completely filled, discard the microcuvette and fill another fresh microcuvette.
- Before analysing the sample make sure that the analyser is in ready state. Then insert the micro cuvette appropriately.
- Once the result is displayed, remove and discard the microcuvette in the red bag. The result will be displayed for 10 seconds.
- Please note down the results for the participant for reference and follow ups, as needed. The report will be given to the participant as agreed by the sites.
- In -case of any invalid result obtained, consult the medical office on site/site PI for necessary directives.

8.1.3 Inoculation of blood into the glucometer: Operate the glucometer as per its make/model manual and work instructions. General guidelines are as below:

- Insert the glucometer strip into the instrument and the display will indicate the blood drop symbol.
- Place a drop of blood from the syringe onto the strip and insert the strip on to the glucometer and a beep sound can be heard.
- After the beep sound, count down will start for showing the results. Please note down the results for reporting purpose and follow ups, as per site's plan of action. Discard the strip in the red bag. In -case of any invalid result obtained, consult the medical office on site/site PI for necessary directives.

9 BIO-MEDICAL WASTE DISPOSAL

a. **Bio medical waste management:** Bio medical waste is an –potential source of infection. Both participants and the study staff are at risk of getting infection from the biomedical waste. The essential components of BMW management include proper collection, storage and disposal of the waste. Study coordinator should make sure that the staff comply with the biomedical waste management protocol. The statutory compliance to state specific requirements shall be site's responsibility.

b. **Collection -**

i. **Colour coding for waste containers:** Biomedical and general waste should be collected and segregated at source and placed in colour coded plastic bags and containers of defined specification prior to collection and disposal.

ii. All staff should adopt the following colour coding-

- ***Yellow bag***: For infectious non plastic waste
- ***Red bag***: For infectious plastic waste.
- ***Green bag***: Non-infectious materials.
- ***Sharp containers***: Syringes, needles and other sharps should be disposed in the sharp containers.

iii. Each phlebotomy team should carry their own colour coded bags and a sharps container with them when they go to participants houses for phlebotomy.

iv. All the waste except sharps should be disposed daily. The sharps can be disposed once the container is filled up by a maximum of 3/4th of its volume.

v. **Storage:** Once blood collection is over for a day, the BMW disposal bags should be tied and kept in specified area. These BMW disposal bags should be removed in the next working day. Storage of infectious material longer than this period is not recommended in the community settings.

vi. **Disposal:** The BMW bags should be disposed off as per protocol in the respective sites through their authorised biomedical waste management system as per written agreement. Records shall be maintained.

vii. **Needle-prick injuries:** In case of any injuries due to sharps, immediate soap water washing under running water is recommended. The respective staff should inform their appointing authority for subsequent testing and prophylactic medicines.

To prevent injuries due to sharps, recapping is strictly prohibited. The phlebotomists should discard all the wastes according to the recommendations after finishing blood collection procedure. Detailed precautionary measures have been given under '**Key components of Standard Precautions'.**

10 STANDARD PRECAUTIONS FOR COVID-19 INFECTION PREVENTION AND CONTROL

Corona virus disease (COVID 19) is caused by a new strain which was discovered in 2019 and has not been previously identified in humans. Standard precautions should be adopted by all staff during the study period. Standard precautions are guidelines and procedures designed to reduce the risk of transmission of microorganisms from both recognized and unrecognized sources of infection. Key components of Standard Precautions are given below:

a) **Perform Hand Hygiene -** Hand rub with alcohol based antiseptic solution should be done by all the health personnel during:
- Before contact with the participant
- Before blood draw
- After blood draw
- After patient contact
- After completing data collection in the household

b) **Gloves** - Use gloves if there is a possibility of:
- Contact with blood, body fluids, secretions and contaminated items: use disposable gloves.
- Discard glove after each procedure in the biomedical waste disposal bag according to the colour coding

c) Face masks and Visor:
- Both the phlebotomists and the field worker, who is holding the hand, should put on a surgical mask and visor
- The mask should cover nose and mouth of the user.
- Clean the visor with soapy water on a rag at least every half day (once at lunch break and once at the end of the day's work)

- Dispose face mask at the end of the day in the assigned BMW disposal bag

d) Disposable apron:

- The phlebotomist should wear a disposable apron to protect the skin from blood and also to prevent soiling of clothes during the procedure
- If the apron is soiled with blood or body fluids, then dispose it in the assigned BMW bag (or else dispose it at the end of the shift i.e. at lunch time and at the end of a day's work)

e) Sharps disposal

- Avoid recapping used needles.
- Avoid removing used needles from disposable syringes with hands.
- Avoid bending, breaking or manipulating used needles by hand.
- Place used sharps in designated puncture-resistant containers.

11 DOCUMENTATION

Documentation will be the responsibility of different people at different stages. Sites shall ensure a mechanism to identify risk and mitigate them. Real time accurate data capture is a must to ensure accuracy and reliability of data generated. The electronic data capture software shall be a tool to provide necessary transcription free record that is auditable. In life witnessing of activities by another team member will provide assurance that activities were done as required. Errors in the key processes including collection, labelling and aliquoting may result in rejection of data and study objectives being jeopardized. Appropriate site level checks will be implemented by site based on key operational activities:

Following is the checklist for documentation and labeling to be developed and made available at sites-

- Preparation of sample collection kits
- Labelling vacutainer tubes at time of collection
- Labelling the aliquots
- Labelling of Cryo boxes
- Completing transcript and shipment forms

12 LOGISTICS

Maintenance of logistics:

All the logistics should be made available regularly for smooth functioning of the programme. A dedicated register will be maintained to keep records of the inflow and outflow of logistics. Study coordinator will monitor the register with the assistance of the administrative assistant and quality assessor. There will be a pre-defined critical stock level for each item. Whenever the level touches this point, the administrative assistant will coordinate with the study coordinator to place an order for that item by the next working day. Cut off levels for different items required for blood collection, have been given below.

Study coordinator will submit a weekly indent report to the PI even in case of zero indents.

Threshold for different items required for blood collection. This list may not be exhaustive and additional items may be maintained in inventory as needed

Sl. no	Name of the items	Critical stock level
1	Surgical gloves (6”)	3 boxes (75)
2	Surgical gloves (6.5”)	3 boxes (75)
3	Surgical gloves (7”)	3 boxes (75)
4	Surgical gloves (7.5”)	3 boxes (75)
5	BD 2 Pc 5ml Discardit II™ Syringe (with Needle) Cat# 300852	250
6	BD 2 Pc 10ml Discardit II™ Syringe (with needle) Cat# 300294	250
7	Disposable needle 23G	250
8	Disposable needle 21G	250
9	Safety Lock Butterfly Needle 23G Catalogue # CAT 367292	250
10	Sterilium	6 bottles
11	Spirit	6 bottles
12	Cotton	6 boxes
13	Micropore	6
14	Surgical mask	4 boxes
15	Liquid soap	6 litres
16	Vacutainer tubes<br>**BD Vacutainer® Plus Plastic Serum Blood Collection Tubes:**<br>4 ml Red Top (BD catalogue # 367812)<br>**BD Vacutainer® Plus Plastic Serum Blood Collection Tubes:**<br>5 ml Red Top (BD catalogue # 367814)<br>• Purple Top EDTA tube 2 ml BD Catalogue# 367841)	300
17	Plastic aprons	50

- Kindly maintain catalogue numbers mentioned for BD vacutainers, needles and syringes as specified in the section 6.
- Unused items: All the slow-moving items should be returned back to the laboratory at least 3 months prior to expiry date. Clinic coordinator should maintain this list. These items should be returned with a letter signed by the study coordinator.

13 REPORTING OF THE TEST RESULTS

- The tests performed in the field at participant’s home by POC device shall be made available to them. Haemoglobin values obtained by the Hemacue as well as Glucometer reading performed on a glucometer will be informed to the participant. Printed reports shall be shared with the

participant by filed staff as per defined format. Any clinically relevant readings for follow ups or referral to health centres shall be as per site SOP.

- HbA1C results will be informed to the participants within a week's time. The results from the clinical laboratory shall be shared electronically with the site as individual results to be handed over to participants.
- Serology test reports will not be available in real time and will be informed to the participants when available.
- The results released from the laboratories shall be captured as per the agreed plan on to platform as per data management plan.

14 QUALITY CONTROL

Safety of the subjects and the staffs, documentation and biomedical waste management has been described earlier in the other sections of this –manual. Following areas/activities must be monitored by site to ensure that the study objectives are met:

- **Quantity of the blood collected:** It is feasible to obtain 6-7 ml or 8-9 ml of blood in adults and 3-4 or 4-5 ml in children in most instances. If there is inadequate volume in > 2% of the blood draws, the field worker will be retrained. The site coordinator or senior team member shall monitor this to use as quality indicator or trigger for re-training.

- **Centrifugation within 24 hours:** A designated person/team is responsible to make sure that the serum is separated at the nearest health facility/field site lab within 24 hours of collection and should be checked by the quality assessor

- **Sample transport and storage:** A designated person is responsible to make sure that the samples are transported to the designated labs maintain the cold-chain and should be checked by the quality assessor.

- **Sample separation and aliquoting:** It is essential that accurate aliquoting is done so that there is no mismatch. To avoid potential errors, while aliquoting, site will define written procedures and ensure double checks for separation, aliquoting and packing in cryoboxes. The site team will design a mechanism where in the samples to be aliquoted are sequentially arranged and total numbers/labelling is matched for the day's activities. Placement of aliquots (A1/A2/A3) into respective cryo-boxes/storage boxes is required to be checked by a second person so as to ensure correct despatch.

Note: there can be check mechanisms over and above those specified above to ensure accurate sample handing and sites are encouraged to adopt best practices in each operational activity.

15 POST-EXPOSURE PROPHYLAXIS FOR NEEDLE STICK INJURIES

Sites need to ensure that all the phlebotomists should be immunized with HBV vaccine before the start of the study. Screening for blood-borne viruses of the phlebotomist has to be performed before the start of the study.

All needle stick injuries should be immediately reported to the coordinator and the PI. Following Needle stick injury, a protocol is followed by which the staff are screened for blood-borne viruses and then followed up. Immediate care of Needle Stick injury is as follows:

- Wash for 10 minutes with soap and water/ disinfectant
- For mucosal exposure, irrigate copiously by running normal saline

Index or the source patient is tested by rapid test for HIV, HbsAg and HCV as follows:

1. **HIV**

   Rapid testing within 45 minutes for HIV for index (source)

   a. If Index is HIV negative
      HIV antibody tested for staff at 0, 6, 12, 24 weeks
   b. Index HIV positive: For Indian setting all HIV positive indexes to be considered highly infectious, Chemoprophylaxis to be started within 1-2 hours following exposure. (Cut off period is 72 hours) Investigations to be ordered for staff while starting: Hb, Platelets, Retics, TC, DC, Creatinine, LFT.

The categorization of exposure with recommended Chemoprophylaxis is as follows:

A) If the exposure is in the following category:

- All percutaneous injuries with contaminated sharps
- Mucous membrane/non-intact skin exposure with a large volume of body fluids and give,
  - Tentide em (tenofovir 300mg + emtricitabine 200mg)
  - Ritovaz (atazanavir 300mg+ ritonavir 100mg).
  - **Each tablet once a day for 28 days.**

B) If the exposure is in the following category:

- For mucous membrane /non-intact skin with a small volume of body fluid then give,

  - Tentide em (tenofovir 300mg + emtricitabine 200mg)
  - **One tablet once a day for 28** days (in any case, if there is a doubt, start on both the tablets)

  Follow up of HCW: The HCW should be tested for HIV antibodies after 6 weeks, 12 weeks, and 24weeks following the exposure, irrespective of the HIV status of the index patient

2. **Hepatitis B**

   If the index case is HBsAg positive:

Health worker	Action
Antibodies >100MIU	Reassure
Antibodies negative or <10MIU	First dose of HBV vaccine and HBV immunoglobulin (0.6ml/kg - I.M.)
Antibody between 10-100MIU	Booster dose of vaccine
HBsAg positive	Counselling

If the index case is HBsAg Negative:

Health worker	Action
HBsAg negative	-
Antibodies >100 MIU	Reassure
Antibody negative or <I00MIU	Vaccination (full or booster as required
HBsAg positive	Counselling

3. Hepatitis C

If the index (source) is positive, staff tested for HCV antibody and LFT on day 0, 3 months and 6 months. No vaccine or prophylaxis is available

16 MANAGEMENT OF BLOOD SPILL

a. Large spills:
- Wear a pair of gloves
- Place a dry mop cloth over the spillage area, to allow the excess of blood and debris to get absorbed.
- Pick this up with gloved hands and discard it in the yellow bag
- Prepare 1% sodium hypochlorite solution (Dakin's) fresh. Pour it over the spillage area Cover this with a rag piece
- Leave it for 10-15 minutes (contact time)
- Remove the rag piece with gloved hands and discard it in the yellow bag Mop the area
- Discard the gloves in a red bag
- Wash hands with soap and water

b. Small spills:
- Wear a pair of gloves
- Cover the area with rag piece soaked with 1% sodium hypochlorite solution freshly prepared. Leave it for 10-15 minutes (contact time)
- Clean the area with mop cloth and discard it in the yellow bag
- Wash hands with soap and water

End of Document
